# Evaluating the impact of integrated development: are we asking the right questions? A systematic review

**DOI:** 10.12688/gatesopenres.12755.2

**Published:** 2018-05-29

**Authors:** Tessa W Ahner-McHaffie, Greg Guest, Tricia Petruney, Alexandra Eterno, Brian Dooley

**Affiliations:** 1 FHI 360, 1825 Connecticut Avenue, NW; Washington, DC, USA; 2 FHI 360, 359 Blackwell St Suite 200; Durham, NC, USA

**Keywords:** integrated, development, multi-disciplinary, multi-sector, evaluation, synergy, interaction effects, SDGs

## Abstract

**Background:** Emerging global transformations - including a new Sustainable Development Agenda - are revealing increasingly interrelated goals and challenges, poised to be addressed by similarly integrated, multi-faceted solutions. Research to date has focused on determining the effectiveness of these approaches, yet a key question remains: are synergistic effects produced by integrating two or more sectors?  We systematically reviewed impact evaluations on integrated development interventions to assess whether synergistic, amplified impacts are being measured and evaluated.

**Methods:** The International Initiative for Impact Evaluation’s (3ie) Impact Evaluation Repository comprised our sampling frame (n = 4,339). Following PRISMA guidelines, we employed a three-stage screening and review process.

**Results:** We identified 601 journal articles that evaluated integrated interventions. Seventy percent used a randomized design to assess impact with regard to whether the intervention achieved its desired outcomes. Only 26 of these evaluations, however, used a full factorial design to statistically detect any synergistic effects produced by integrating sectors. Of those, seven showed synergistic effects.

**Conclusions:** To date, evaluations of integrated development approaches have demonstrated positive impacts in numerous contexts, but gaps remain with regard to documenting whether integrated programming produces synergistic, amplified outcomes. Research on these program models needs to extend beyond impact only, and more explicitly examine and measure the synergies and efficiencies associated with linking two or more sectors. Doing so will be critical for identifying effective integrated development strategies that will help achieve the multi-sector SDG agenda.

## Introduction

### Rationale

Twenty-first century global trends such as rapid urbanization and dramatic climate change are forcing the international community to rethink solutions to challenges that are increasingly multi-faceted and interrelated. Indeed, the Sustainable Development Goals (SDGs) – an ambitious framework of 17 goals to end extreme poverty, fight inequality and injustice, and reverse climate change over the next 15 years – emphasize the integration of previously distinct development aims. The agenda states that “[t]he goals and targets we have decided on are integrated and indivisible and balance the three crucial dimensions of sustainable development: the economic, social and environmental” ( United Nations, 2015). This evolution in thinking indicates a firm shift away from narrowly isolated sectors of development toward what the authors refer to as “win-win cooperation.” An analysis of how each of the 169 SDG targets is related to others reveals a web of closely interrelated objectives, yet also points out that any policy and program integration founded on these underlying linkages would need to rest on evidence with regard to their means of implementation ( Le Blanc, 2015).

Thus, decisions about when and how to most effectively implement the integrated, multi-sector SDG agenda need to be driven by evidence, rather than by assumptions about the amplified results of ‘doing more together’. Importantly, a large volume of research carried out to assess various types of integrated programs – in this case meaning programs that intentionally link their design and delivery (i.e., implementation) across more than one core development sector – suggests that in many cases these cross-sector approaches are successful in achieving positive impacts. But are program evaluators addressing a critical question: is 1 + 1 > 2? For example, does adding a nutritional component to an agricultural program reach more people than doing the same work separately? Does integrating family planning into an environmental conversation program improve the targeted impacts of one or more components? Are these interventions generating amplified impacts that go beyond the sum of single sector interventions? To identify and apply effective integrated program approaches in low- and middle-income countries (LMIC), it behooves us to first understand which programs are being evaluated and how. In this paper we present the results of a systematic review designed to identify to what degree impact evaluations have measured synergies and interaction effects, and if evaluations have, then, which methodologies were used to do so.

### Objectives

The objectives of this systematic review were threefold: to identify impact evaluations of integrated development programs; to assess whether these evaluations seek to statistically measure synergistic effects or efficiencies associated with integrated interventions; and, for those that do, to document if synergies are detected.

## Methods

**Figure 1.**
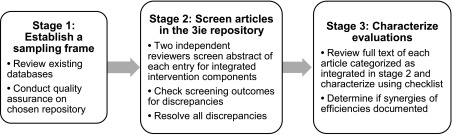
Review process for systematic review on integrated, multi-sector programs.

Our review consisted of a three-stage process ( Figure 1), based on the PRISMA guidelines ( Moher *et al*., 2009), as well as recommendations from Waddington *et al*. (2012). We first established a sampling frame from which to screen and review published articles. Given our objectives, we required a large, relatively exhaustive development database, or combination of databases, that included a broad range of evaluations not limited to any particular development sector. The first stage in our process, therefore, entailed establishing a sampling frame from which to identify and review the highest number of relevant publications and is therefore a modified step to a conventional systematic review. Once the sampling frame was established, we screened each article’s abstract and identified evaluations of development programs that we defined as “integrated development.” We then reviewed the full text of each article in the integrated development subset and documented characteristics essential to the objectives of our review.

### Stage 1: Establish a sampling frame

The universe of development literature and evidence is extensive. Published articles from this broad field are scattered throughout numerous databases, span a multitude of sectors, and use a diverse range of keywords and terminology (or no indicative terminology at all). We explored many of these specialized databases and possible combinations of databases from which we could specifically identify the most evaluations of programs that integrated two or more traditional development sectors (described below). Fortunately, we found a high-quality database that was not specific to any particular sector that would serve well as a single sampling frame for our review. Moreover, while we were initially interested in a broad range of research designs we felt comfortable with using only impact evaluations as it helped to ensure a baseline level of rigor in study design.

The International Initiative for Impact Evaluation (3ie) Impact Evaluation Repository is an index of all published impact evaluations of development interventions. To be included in the repository, an impact evaluation must be published (as a journal article, book chapter, report, or working paper), take place in a developing country, examine the effectiveness of a specific development intervention, and use a specifically defined experimental or quasi-experimental estimation strategy.

**Figure 2.**
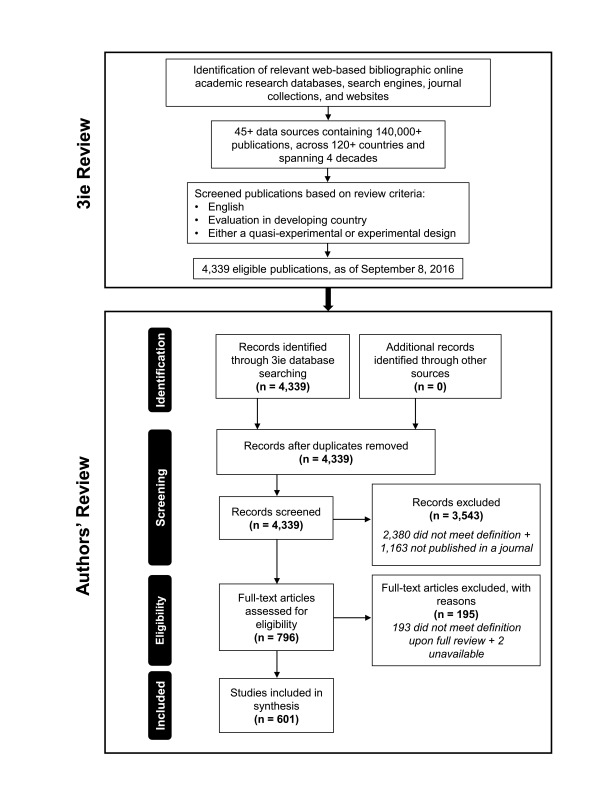
PRISMA flow diagram for systematic review with 3ie impact evaluation repository review process.

During the creation of the repository, 3ie systematically searched more than 45 databases, search engines, journal collections, and websites with an aim to identify all published development impact evaluations ( Figure 2) ( Mishra & Cameron, 2014; Jorge Miranda, 2017, personal communication). At the time of our analysis (September 8, 2016), 3ie had reviewed more than 140,000 potential studies, rendering an index of 4,339 eligible studies ( Mishra & Cameron, 2014; Jorge Miranda, 2017, personal communication). The repository, including a full description of its inclusion criteria and review methodology, is available here. The search was limited to English language articles that evaluate a development program, project, or policy; to evaluations that occur in LMICs; and articles that use rigorous techniques to identify a counterfactual. Grey literature is included in the database, and the 45 sources include everything from EconLit to Google Scholar (full list can be found here). Key terms to identify impact evaluations included terms like ‘impact’, ‘effect’, or ‘random’, but differ slightly by database. The last 3ie update was completed in July 2016.

As part of our due diligence, we sought to confirm that 3ie’s repository was a thorough and sufficient sole-sampling source. A library science specialist audited the methodology 3ie staff used to create the repository. Her objective was to assess whether the searches used were both sensitive (i.e., broad) and specific (i.e., focused) enough to ensure that the vast majority of relevant and eligible impact evaluations were included in the final repository. She reviewed the databases that were used and how they were searched with regard to subject scope, time frame limits, and geographic coverage. She concluded that although some lesser-known and regional databases were excluded from the 3ie repository the likelihood that any significant number of new references would be found was negligible. The databases that the 3ie repository was sampling from were searching many of these smaller databases, so she could find no evidence that the larger databases missed anything the smaller databases held. She concluded that the overall methodology design was strong and its implementation consistent. We therefore feel confident that using the 3ie repository as our sampling frame provided us with a sufficient index of development evaluations.

### Stage 2: Screen articles in the repository for integrated development approaches

The purpose of this stage of the review was to identify all of the publications in the 3ie repository that evaluated integrated programs. No terms to denote integrated development have universal agreement. The concept of integrated or multi-sector development in published papers is described by many different terms (e.g., cross-sector, linked, combined, blended). Moreover, authors rarely self-identify their interventions in this way within an article, let alone an abstract. We could not, therefore, rely on key search terms to identify evaluations of programs that were integrated in nature. Instead, we manually reviewed the abstracts of every study in the 3ie repository (as of September 8, 2016) against FHI 360’s working definition of integrated development:

*“Integrated development approaches intentionally link the design and delivery (i.e., implementation) of programs across more than one core sector.”*

Note that our definition of integrated development encompasses studies that would be classified as “multi-sector” or “multi-disciplinary” by others. More precisely, our definition focuses on the integrated nature of the intervention itself and excludes programs that only integrate different subsectors within a core sector (e.g., health programs that link family planning and HIV/AIDS). Our definition also excludes programs that measure outcomes in multiple sectors stemming from a single-sector program (e.g., measuring both education and nutrition outcomes from an education-only project). Finally, explicit intentionality did not factor in the actual application of the definition, since very few evaluations were detailed enough to illustrate motivation or thought process.

Other definitions of integrated programs imply collaboration between the public and private sectors, integration of services between different established levels in a system (e.g., government ministries and local service providers), include different core development sectors, or require clear implementation processes to support integration (e.g., co-location of services, cross-training staff). While these approaches are often grouped together and utilize some of the same approaches and techniques, we broke out multi-sector integration here from public and private sector (or from multi-level systems integration) to create a group of evaluations that are similar enough to be evaluated together meaningfully.

There are no universal or definitive lists of development ‘sectors’. Global bodies and implementing organizations characterize thematic areas in fluid ways, at times bundling some fields (e.g., health and nutrition), and at other times ensuring they are distinctly separate. For this review, we used core sector categories and illustrative interventions and outcomes found in Table 1. This was shaped by what FHI 360 classifies as core sectors, as well as what 3ie and other development databases classify as core sectors. We used these sector categories to classify interventions as well as outcome measures.

**Table 1.** Sector intervention and outcome examples.

Sector	Intervention examples	Outcome examples
Agriculture and food security	Farming, food supply chains, famine prevention	Food security, agricultural productivity, agricultural production, access to extension services
Economic development	Livelihoods, cash transfers, microfinance	Income, savings, household assets
Education	Early education, primary school, secondary school	Enrollment, grade completion, attendance
Environment	Environmental/land management, conservation, climate change	Deforestation rate, erosion, environmental knowledge and perceptions
Governance	Peace building, conflict management, election monitoring, democracy	Participation in government, inclusive government, effective management scores, corruption
Health	HIV, tuberculosis, maternal and child health, sexual and reproductive health, non-communicable disease, malaria, vaccination	HIV prevalence, vaccination rates, under- five mortality
Nutrition	Micronutrient provision, food fortification, feeding programs, diet diversification	Anemia, malnutrition, stunting, wasting, dietary diversity
Water, sanitation, and hygiene (WASH)	Water quality, management, supply	Access to water sources, access to sanitation, hand washing behaviors

All of the interventions in the studies reviewed fell within these sector categories. We added an “other” category to describe outcomes measured, to capture more amorphous, non-sector specific measures, such as “child labor.” For our review, cross-cutting topics such as gender, youth, civil society, and technology were considered aspects of, and relevant to, the interventions and outcomes in each sector, but not sectors in and of themselves. During the review we had also initially included ‘humanitarian’ as a sector. With further discussion and analysis, it was clear that this sampling frame was not inclusive of the humanitarian sector, nor is humanitarian work represented at the same level of the conventional development sectors included above. Therefore, the final analysis was completed without the humanitarian sector category in either intervention sector or outcome sector measured.

To enhance reliability, two individuals independently reviewed all of the abstracts and identified the sectors represented in the interventions being evaluated. If more than one sector was identified, the study was categorized as “yes” for integrated development; all other studies were marked as “no”. These two reviewers met at predetermined intervals to compare their results, and had an average of 89% agreement.

All discrepancies in coding were resolved at each comparison point after the team discussed the interpretation of the integrated development definition (resulting in 100% agreement). In the few cases in which the two reviewers could not agree on a study’s categorization, a third-party reviewed the abstract and made the final decision. Any study that members of the review team both categorized as integrated development was included in the second round of review. For cases in which the abstract alone did not contain enough information to make a determination, studies were advanced to the next round of review so that a final determination could be made during review of the full text.

Importantly, although the repository includes impact evaluations published in any form, our inclusion criteria for this review required a study to be published in a scientific journal. We excluded grey literature to manage the high volume of eligible papers but acknowledge that there is value in their examination for future reviews. Therefore, only those publications that were peer reviewed moved on to Stage 3.

### Stage 3: Characterize evaluations of interventions identified as integrated

Full text articles of the subset of studies on integrated programs that were published in scientific journals were reviewed by two individuals. Each article was compared against a checklist, to ascertain the study’s scope and methodology (our checklist is presented with the corresponding results in Table 2).

**Table 2.** Summary of integrated development impact evaluation characteristics.

Characteristic (N = 601)	Frequency	
Self-identified term for the nature of intervention being evaluated (by study author, in the title or abstract)	Integrated Combined Multi-component Multi-faceted Other Did not self-identify	44 (7%) 25 (4%) 9 (1%) 2 (<1%) 19 (3%) 507 (84%)
General study design	Experimental Quasi-experimental Both	419 (70%) 174 (29%) 8 (1%)
Study arm combinations included in design	Integrated arm(s) + control arm Integrated arms(s) only Single-sector arm(s) + Integrated arm(s) Full factorial Other	366 (61%) 68 (11%) 132 (22%) 26 (4%) 9 (1%)
Partial factorial design (Randomized study that includes at least 1 single-sector arm — but not ALL single-sector arms — and at least 1 integrated arm and at least 1 control arm)	12 (2%)	
Full factorial design (Randomized study that include ALL single-sector arms, at least 1 integrated arm, and at least 1 control arm)	26 (4%)	
Qualitative component included in evaluation	60 (10%)	
Sectors included in study intervention	Agriculture & food security Economic development Education Environment Governance Health Nutrition Water, sanitation, and hygiene	65 (11%) 238 (40%) 433 (72%) 25 (4%) 12 (2%) 456 (76%) 266 (44%) 44 (7%)
Number of intervention sectors included in design	2 3 4 5 or more	373 (62%) 132 (22%) 87 (14%) 9 (1%)
Sectors in which outcomes measured	Agriculture & food security Economic development Education Environment Governance Health Nutrition Water, sanitation, and hygiene Integrated outcome (bespoke) Other	40 (7%) 102 (17%) 149 (25%) 13 (2%) 8 (1%) 373 (62%) 174 (29%) 23 (4%) 1 (<1%) 54 (9%)
Number of sectors in which outcomes were measured	1 2 3 4 or more	347 (58%) 185 (31%) 60 (10%) 9 (1%)
Geographic area of study	Middle East/North Africa Sub-Saharan Africa Asia Latin American & the Caribbean Europe Oceania Number of countries represented	21 (3%) 213 (35%) 177 (29%) 188 (31%) 4 (<1%) 2 (<1%) 70
Cost analysis conducted	43 (7%)	
Implementation/process evaluation conducted	41 (7%)	

In particular, we noted the number of control, single-sector treatment, and integrated sector treatment arms in each evaluation. We further identified those evaluations which employed either a partial factorial or full factorial experimental design. For the purposes of our review, partial factorial designs included at least one single-sector arm (but not all single-sector arms), at least one integrated arm, and at least one control (no intervention) arm. Full factorial designs included all possible single-sector arms, at least one integrated arm, and at least one control arm. Factorial designs are exceptionally rigorous and permit evaluators to determine the effects of multiple interventions on an outcome. Since they include all possible combinations of intervention arms, full factorial designs are able to reveal differential effects of single-sector and multi-sector interventions and measure potential synergistic effects associated with integrated approaches. 

Therefore, we specifically reviewed each full factorial evaluation to determine if the authors measured or detected synergy associated with the integrated study arm. For our review, we defined synergy as a statistically significant (p < 0.05) interaction effect in the integrated arm (between two or more intervention sectors), or as instances in which the effect size of the integrated arm of a program was greater than the sum of the effect sizes among the single-sector arms.

Given the extreme heterogeneity of the types of programs evaluated and outcomes assessed, we did not seek to collectively synthesize their substantive findings. Instead, the primary objective was to determine if and how impact evaluations of integrated programs are designed to measure or systematically document the synergy and efficiency assumed in multi-sector development.

## Results

We reviewed 4,339 abstracts, comprising the entire 3ie repository as of September 8, 2016. After a two-step screening process, 601 articles were included in our final dataset for characterization ( Figure 2, Supplementary File 2). From the initial set of 4,339 articles from the 3ie repository, 3,543 were excluded (2,380 did not meet the definition of integrated and 1,163 were not published in a scientific journal). The full text of the remaining 796 were assessed for eligibility. One hundred and ninety-five were excluded with full text review (193 did not meet the definition of integrated and two were not available to reviewers). This left 601 studies included in the analysis. The list of articles is included here as a supplement ( Supplementary File 3), and each article may also be found in a searchable online database.

The majority of evaluations (70%) employed a randomized controlled design to assess the effectiveness of their interventions. However, only 26 (4%) of the 601 studies reviewed used a full factorial design and only 12 (2%) employed a partial factorial design. The majority of evaluations (61%) assessed the effectiveness of an integrated intervention by comparing one or more integrated arms to a no-treatment control only. A minority of evaluations included a comparison of integrated arms only (11%), or contained single-sector arms and integrated arms but no control arm (22%). Few evaluations included qualitative (10%) or cost analyses (7%) components. Most articles (84%) did not identify the interventions being evaluated as “integrated”, or any other related term ( Table 2).

With regard to what types of interventions and desired outcomes were being assessed, the three sectors most often represented in the intervention design — in order of highest to lowest frequency — were health, education, and nutrition. The same three sectors were also most common in terms of outcomes measured, with nutrition slightly outpacing education for second most common. The top three most common sectors that each sector was integrated with is also illustrative (see Table 3). For example, agriculture and food security is most commonly integrated with economic development, followed by nutrition and health. The proportion of studies that measured outcomes within each sector are displayed as well ( Table 4). For example, 29.5% of studies that include WASH as an intervention sector include an education outcome measure. Seventy-six percent of studies that include an environmental intervention include an economic development outcome measure.

**Table 3.** Most commonly integrated sectors and sector combinations.

Sector	Most Commonly Integrated with…	Second most commonly integrated with…	Third most commonly integrated with…
**Agriculture & food** **security (n=65)**	Economic development (n=57)	Nutrition (n=20)	Health (n=19)
**Economic development** **(n=238)**	Health (n=171)	Education (n=137)	Nutrition (n=103)
**Education (n=433)**	Health (n=344)	Nutrition (n=193)	Economic development (n=137)
**Environment (n=25)**	Economic development (n=20)	Agriculture & food security (n=12)	Governance (n=5) Health (n=5)
**Governance (n=12)**	Economic development (n=9)	Agriculture & food security (n=5) Environment (n=5)	Education (n=4)
**Health (n=456)**	Education (n=344)	Nutrition (n=192)	Economic Development (n=171)
**Nutrition (n=266)**	Education (n=193)	Health (n=192)	Economic development (n=103)
**Water, sanitation, and** **hygiene (n=44)**	Education (n=29)	Health (n=27)	Nutrition (n=11)

**Table 4.** Proportion of outcomes by sector intervention.

		Outcomes							
		Agriculture & food security (n=40)	Economic development (n=102)	Education (n=149)	Environment (n=13)	Governance (n=8)	Health (n=373)	Nutrition (n=174)	Water, sanitation, and hygiene (n=23)
**Interventions**	Agriculture & food security (n=65)	32/65 **49.2%**	40/65 **61.5%**	6/65 **9.2%**	7/65 **10.8%**	0/65 **0%**	10/65 **15.4%**	15/65 **23.1%**	1/65 **1.5%**
	Economic development (n=238)	38/238 **16.0%**	100/238 **42.0%**	65/238 **27.3%**	13/238 **5.5%**	8/238 **3.4%**	87/238 **36.6%**	32/238 **13.4%**	2/238 **0.8%**
	Education (n=433)	11/433 **2.5%**	40/433 **9.2%**	142/433 **32.8%**	2/433 **0.5%**	4/433 **0.9%**	279/433 **64.4%**	116/433 **26.8%**	16/433 **3.7%**
	Environment (n=25)	8/25 **32.0%**	19/25 **76.0%**	4/25 **16.0%**	10/25 **40.0%**	1/25 **4.0%**	6/25 **24.0%**	1/25 **4.0%**	1/25 **4.0%**
	Governance (n=12)	4/12 **33.3%**	9/12 **75.0%**	4/12 **33.3%**	1/12 **8.3%**	2/12 **16.7%**	3/12 **25.0%**	1/12 **8.3%**	1/12 **8.3%**
	Health (n=456)	15/456 **3.3%**	57/456 **12.5%**	100/456 **21.9%**	2/456 **0.4%**	4/456 **0.9%**	346/456 **75.9%**	122/456 **26.8%**	12/456 **2.6%**
	Nutrition (n=266)	12/266 **4.5%**	27/266 **10.2%**	87/266 **32.7%**	3/266 **1.1%**	3/266 **1.1%**	142/266 **53.4%**	156/266 **5.9%**	5/266 **1.9%**
	Water, sanitation, and hygiene (n=44)	4/44 **9.1%**	4/44 **9.1%**	13/44 **29.5%**	1/44 **2.3%**	0/44 **0%**	29/44 **65.9%**	9/44 **20.5%**	19/44 **43.2%**

For the 38 studies that represented either a partial or a full factorial design, we assessed whether the effectiveness of an integrated intervention — in terms of study outcomes — was evaluated. Of the 26 that were full factorial, seven reported findings that showed that the integrated arm was most effective ( De Brauw *et al*., 2015; Haque *et al*., 2010; Leventhal *et al*., 2016; Nga *et al*., 2009; Nga *et al*., 2011; Olsen *et al*., 2003; Widen *et al*., 2015). Eight demonstrated mixed findings, or did not report the effectiveness of the integrated arm as compared to the other arms ( Awasthi *et al*., 2013; Duflo *et al*., 2015; Gilgen & Mascie-Taylor, 2001; Halliday *et al*., 2014; Jinabhai *et al*., 2001; Kim *et al*., 2015; Leventhal *et al*., 2015; Tahlil *et al*., 2015). In some cases, the added value of integration was reported in another study, or it was stated that the combination was not intended to have effects on the outcomes of the separate sectors, so the data was not fully analyzed or reported. In terms of mixed findings, some studies demonstrated tradeoffs, where integration added value to certain outcomes, but was deleterious for others. Finally, 11 evaluations found no added value of integration ( Attanasio *et al*., 2014; Dangour *et al*., 2011; Desai & Tarozzi, 2011; Fenn *et al*., 2012; Friis *et al*., 2003; Gilgen *et al*., 2001; Gowani *et al*., 2014; Mwaniki *et al*., 2002; Nahar *et al*., 2012; Rohner *et al*., 2010; Walker *et al*., 2006).

The seven studies that found amplified impacts included four studies that examined the combination of nutritional supplementation and deworming ( Haque *et al*., 2010; Nga *et al*., 2009; Nga *et al*., 2011; Olsen *et al*., 2003), one study that examined the effect of nutritional supplementation and antiretroviral prophylaxis use in women and infants ( Widen *et al*., 2015), one study examined a school-based psychosocial adolescent health intervention for girls ( Leventhal *et al*., 2016), and one study examined the promotion of orange-flesh sweet potato through integrated agricultural and nutritional activities ( De Brauw *et al*., 2015). Four interventions took place in Asia and three in sub-Saharan Africa. All seven found improved outcomes in the integrated arm as compared to single sector and control arms.

Only three of the full factorial studies incorporated cost analysis, and two of the three found that the integrated arm was cost-effective. The third did not perform a cost analysis on the integrated arm.

We also reviewed the findings reported in the 60 studies that included a qualitative component. We found only 1 study that intentionally documented synergy via the qualitative inquiry – the others used the method to investigate other aspects of the intervention.

## Discussion

Our screening of 4,339 records in the 3ie Impact Evaluation Repository identified 601 journal articles that describe studies of programs we defined as integrated. Our full text review of these 601 articles revealed several interesting trends. First, researchers do not use standardized terms for describing integrated development programs. In fact, the majority of authors did not use any term at all to indicate the integrated or multi-sector nature of the interventions they were evaluating. This finding validates our manual screening methodology. Had we used a key term search strategy, we likely would have missed many relevant studies. Interestingly, 46% of the full factorial evaluations addressed integration or synergy in their abstracts, as compared to only 16% of all studies identified as integrated.

Next, only 26 evaluations employed a full factorial design. Though randomized controlled designs are sufficient to confidently detect the impact of these types of programs, a full factorial design is the only design that enables researchers to statistically measure whether the impact is related to the synergy presumed to result from integrated, multi-sector programming. With so few full or partial factorial studies completed, and such diversity in the outcomes found, we are not able to evaluate effectiveness. We can, however, see trends in where research is being done, and the need for research that does consider integration as a component of the program to be evaluated.

We recognize that full factorial designs are often costly and time-consuming, and may not be feasible or even advisable in many scenarios. Having such strong counterfactuals does offer credible information for internal validity. Like many RCTs, however, they have a limited ability to suggest external validity. Factorial studies may not be necessary for the types of integrated approaches that have been confirmed in the past to deliver synergy. Given their expense, they may best be used in cases where proof of concept is needed, but not necessary for understanding how to apply a promising or proven model in other contexts. Methods used in implementation science and adaptive programming can similarly shed light into the effectiveness of integrated approaches in new settings. Factorial or not, using a mixed method approach to include examination of cost efficiencies and qualitatively assessing synergies offer value for determining how integration factors in to the program findings. While this review was not focused on these types of evaluations, we found that few impact evaluations on integrated approaches included these components. 

### Limitations

Creating and applying a definition of integrated development was a subjective process. To address this, we utilized two independent coders and employed inter-coder agreement procedures to enhance reliability in our screening process. Assigning core sectors to studies was also a subjective process, and in some cases assigning sectors to an intervention was difficult (e.g., depending on its particular aim, aid to small-scale farmers could conceivably be an economic development, agriculture, or nutrition intervention). We attempted to mitigate this by providing definitions and examples of core sectors to both reviewers, and once a type of intervention was categorized in one way it remained consistent across studies.

Another potential limitation is that the 3ie repository may not be exhaustive. The 3ie database limits by language (English) and the last update at the time of our searches was completed in July 2016. Limiting to English could systematically exclude publications from certain regions, or exclude certain groups of evaluations. Eligible publications in regional or small databases not included in the search strategy could have been overlooked. Given the size of the repository, however — more than 4,300 publications — including a small number of studies that may be absent in the 3ie repository would not have changed the substance of our findings.

We also recognize that in the past 4–5 years an increasing proportion of impact evaluations are being written up as working papers ( Cameron *et al*., 2016) and may not be published in peer-reviewed journals. Although our judgment is that it would not be significant, we do acknowledge that reviewing to include grey literature could conceivably shift some of the trends we highlight here. Our entire database of impact evaluations for integrated development found within the 3ie repository – peer-reviewed and grey literature – can be found here.

Finally, a large portion of the studies in the impact evaluation repository (and therefore this review) focus on the health sector. This is more a representation of the volume of research conducted in the health sector in general rather than an indication that health-focused approaches have a higher importance or more effectiveness than other sectors. Due to different evaluation cultures within different sectoral communities, and the ease to which some interventions lend themselves to certain types of evaluations, health is almost certainly overrepresented here than if this review had a broader methodological sampling frame.

### Further research

There are many avenues which we could not pursue during this research that would further support our understanding of how integrated projects are being studied, and the results of those studies. A review of observational studies, or a systematic review of qualitative or cost analyses studies could also illustrate meaningful trends. There have been targeted reviews on various combinations of sectors that have included deeper examination of the results (since the subject is narrower) (e.g., Lukas, 2008; Ruel *et al.*, 2018). This review can be used to highlight areas that could be further explored with a finer comb. Further reviews of different types of integration – including private and public sector, and multi-sector outcomes for example, mentioned above – could also produce interesting results.

## Conclusions

Our systematic review is not intended to determine whether or not integrated development approaches work. We know from the high number of randomized evaluations included here that report positive findings that in many contexts integrated, multi-sector interventions have produced impact. What our systematic review does indicate, however, is that very few impact evaluations to date were designed to specifically examine the synergistic and interaction effects that are potentially associated with integrated programming. In other words, to what extent is the integration itself producing the results versus other factors? And if so, why and how? Impact evaluations of new or yet-to-be-proven integrated programs need to be better designed to intentionally assess not only their overall impact but the explicit value added of linking two or more development sectors, in terms of service delivery outcomes, participant perspectives, and cost.

Addressing these gaps is essential as the international community pivots toward a more cross-cutting global development agenda. Implementing this agenda will likely deploy more promising and innovative silo-breaking programs. We must ensure that our research designs and measurement strategies keep pace accordingly.

## Supplementary material

Supplementary File 1: PRISMA checklist.

Supplementary File 2: PRISMA flowchart, showing the number of records identified, included and excluded.

Supplementary File 3: Full list of 601 references included in the review.
