# Supplementary material for: Evaluating the impact of integrated development: are we asking the right questions? A systematic review
Source: Gates Open Res. 2018 May 29;1:6. Originally published 2017 Nov 6. [Version 2] doi: 10.12688/gatesopenres.12755.2 (PMC6034098; doi:10.12688/gatesopenres.12755.2)
Supplement: Supplementary file 2 [file gatesopenres-1-13898-s0001.tgz › 8fa438f3-9a5b-4206-a728-0a3056e1b4d5.pdf]

PRISMA Flow Diagram: “Evaluating integrated development: are we asking the right questions? A systematic review”

(Ahner-Mchaffie *et al*., 2017)

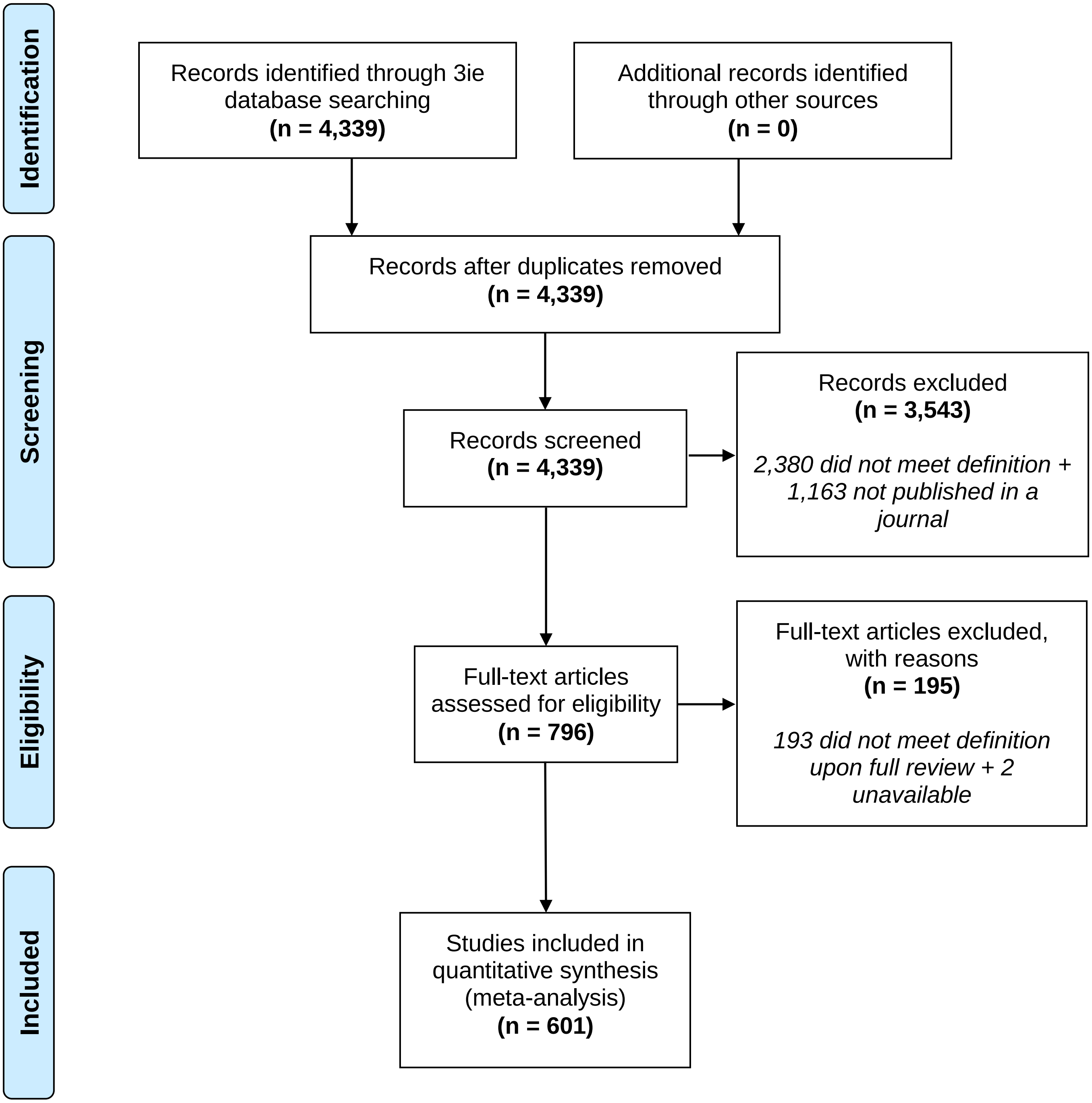

From: Moher D, Liberati A, Tetzlaff J, Altman DG, The PRISMA Group (2009). *P*referred *R*eporting *I*tems for *S*ystematic Reviews and *M*eta-*A*nalyses: The PRISMA Statement. PLoS Med 6(7): e1000097. doi:10.1371/journal.pmed1000097

For more information, visit www.prisma-statement.org.
