# Supplementary material for: Evaluating the impact of integrated development: are we asking the right questions? A systematic review
Source: Gates Open Res. 2018 May 29;1:6. Originally published 2017 Nov 6. [Version 2] doi: 10.12688/gatesopenres.12755.2 (PMC6034098; doi:10.12688/gatesopenres.12755.2)
Supplement: Supplementary file 3 [file gatesopenres-1-13898-s0002.tgz › 6885d92b-66db-44fd-9a77-5bfd35cdc5fb.pdf]

Supplementary File 3: Full list of 601 references included in the review: "Evaluating integrated development: are we asking the right questions? A systematic review." (Ahner-Mchaffie *et al*., 2017)

Searchable citations can also be found at the Integrated Development Evidence Map at http://fhi360integrationevidence.com/

Abdou, A., Munoz, B. E., Nassirou, B., Kadri, B., Moussa, F., Baare, I., Riverson, J., Opong, E., & West, S. K. (2010). How much is not enough? A community randomized trial of a Water and Health Education programme for Trachoma and Ocular C. trachomatis infection in Niger. *Trop Med Int Health*, 15(1), 98-104.

Abebaw, D., Fentie, Y., & Kassa, B. (2010). The impact of a food security program on household food consumption in Northwestern Ethiopia: A matching estimator approach. *Food Policy*, 35(4), 286-293.

Aboud, F. E., & Akhter, S. (2011). A cluster-randomized evaluation of a responsive stimulation and feeding intervention in bangladesh. *Pediatrics*, 127(5), e1191-1197.

Aboud, F. E., Singla, D. R., Nahil, M. I., & Borisova, I. (2013). Effectiveness of a parenting program in Bangladesh to address early childhood health, growth and development. *Social Science & Medicine*, 97(Supplement C), 250-258.

Aburto, N. J., Fulton, J. E., Safdie, M., Duque, T., Bonvecchio, A., & Rivera, J. A. (2011). Effect of a school-based intervention on physical activity: cluster-randomized trial. *Med Sci Sports Exerc*, 43(10), 1898-1906.

Adejumo, A. (2012). Influence of psycho-demographic factors and effectiveness of psycho-behavioural interventions on sexual risk behaviour of in-school adolescents in Ibadan City. *IFE PsychologIA: An International Journal*, 20(1), 1-18.

Adimassu, Z., & Kessler, A. (2015). Impact of the productive safety net program on farmers' investments in sustainable land management in the Central Rift Valley of Ethiopia. *Environmental Development*, 16(Supplement C), 54-62.

Adrogue, C., & Orlicki, M. E. (2013). Do in-school feeding programs have an impact on academic performance and dropouts? The case of public schools in Argentina. *Archivos Analíticos de Políticas Educativas*, 21, 1-20.

Afridi, F. (2010). Child welfare programs and child nutrition: Evidence from a mandated school meal program in India. *Journal of Development Economics*, 92(2), 152-165.

Afridi, F. (2011). The impact of school meals on school participation: evidence from rural India. *Journal of Development Studies*, 47(11), 1636-1656.

Ager, A., Akesson, B., Stark, L., Flouri, E., Okot, B., McCollister, F., & Boothby, N. (2011). The impact of the school-based Psychosocial Structured Activities (PSSA) program on conflict-affected children in Northern Uganda. *J Child Psychol Psychiatry*, 52(11), 1124-1133.

Aggarwal, A. (2010). Impact evaluation of India's 'Yeshasvini' community-based health insurance programme. *Health Econ*, 19 Suppl, 5-35.

Agha, S. (2002). An Evaluation Of The Effectiveness Of A Peer Sexual Health Intervention Among Secondary-School Students In Zambia. *AIDS Education and Prevention*, 14(4), 269-281.

Agha, S., & Van Rossem, R. (2004). Impact of a school-based peer sexual health intervention on normative beliefs, risk perceptions, and sexual behavior of Zambian adolescents. *J Adolesc Health*, 34(5), 441-452.

Agrawal, N., & Pushpanjali, K. (2011). Feasibility of including APF gel application in a school oral health promotion program as a caries-preventive agent: a community intervention trial. *J Oral Sci*, 53(2), 185-191.

Ahmed, F., Khan, M. R., Akhtaruzzaman, M., Karim, R., Williams, G., Torlesse, H., Darnton-Hill, I., Dalmiya, N., Banu, C. P., & Nahar, B. (2010). Long-Term Intermittent Multiple Micronutrient Supplementation Enhances Hemoglobin and Micronutrient Status More Than Iron + Folic Acid Supplementation in Bangladeshi Rural Adolescent Girls with Nutritional Anemia. *The Journal of Nutrition*, 140(10), 1879-1886.

Aiken, A. M., Davey, C., Hargreaves, J. R., & Hayes, R. J. (2015). Re-analysis of health and educational impacts of a school-based deworming programme in western Kenya: a pure replication. *International Journal of Epidemiology*, 44(5), 1572-1580.

Akinlade R. J., Yusuf S. A., Omonona B. T., & Oyekale A. S. (2011). Impact of Fadama II on income and inequality of rural households in Nigeria. *Journal of Agricultural and Biological Science*, 6(7), 39-52.

Akter, S., Roy, S., Thakur, S., Sultana, M., Khatun, W., Rahman, R., Saliheen, S., & Alam, N. (2012). Effects of third trimester counseling on pregnancy weight gain, birthweight, and breastfeeding among urban poor women in Bangladesh. *Food & Nutrition Bulletin*, 33(3), 194-201.

Albonico, M., Stoltzfus, R. J., Savioli, L., Chwaya, H. M., d'Harcourt, E., & Tielsch, J. M. (1999). A controlled evaluation of two school-based anthelminthic chemotherapy regimens on intensity of intestinal helminth infections. *Int J Epidemiol*, 28(3), 591-596.

Alderman, H. (2007). Improving nutrition through community growth promotion: Longitudinal study of the nutrition and early child development program in Uganda. *World Development*, 35(8), 1376-1389.

Alderman, H., Gilligan, D. O., & Lehrer, K. (2013). The impact of food for education programs on school participation in northern Uganda. *Economic Development and Cultural Change*, 61(1), 187-218.

Alderman, H., Ndiaye, B., Linnemayr, S., Ka, A., Rokx, C., Dieng, K., & Mulder-Sibanda, M. (2009). Effectiveness of a community-based intervention to improve nutrition in young children in Senegal: a difference in difference analysis. *Public Health Nutr*, 12(5), 667-673.

Alexander, K. T., Dreibelbis, R., Freeman, M. C., Ojeny, B., & Rheingans, R. (2013). Improving service delivery of water, sanitation, and hygiene in primary schools: a cluster-randomized trial in western Kenya. *Journal of Water and Health*, 11(3), 507-519.

Alix-Garcia, J., McIntosh, C., Sims, K. R., & Welch, J. R. (2013). The ecological footprint of poverty alleviation: evidence from Mexico's Oportunidades program. *Review of Economics and Statistics*, 95(2), 417-435.

Alzúa, M.L., Cruces, G. & Ripani, L. Welfare Programs and Labor Supply in Developing Countries: Experimental Evidence from Latin America. *J Popul Econ*, 26(4), 1255.

Al-sheyab, N., Gallagher, R., Crisp, J., & Shah, S. (2012). Peer-led education for adolescents with asthma in Jordan: a cluster-randomized controlled trial. *Pediatrics*, 129(1), e106-112.

Amarante, V., Ferrando, M., & Vigorito, A. (2013). Teenage School Attendance and Cash Transfers: An Impact Evaluation of PANES. *Economia*, 14(1), 61-96.

Amare, M., & Asfaw, S. (2012). Poverty reduction impact of food aid in rural Ethiopia. *Journal of Development Effectiveness*, 4(2), 235-256.

Andang'o, P. E. A., Osendarp, S. J. M., Ayah, R., West, C. E., Mwaniki, D. L., De Wolf, C. A., Kraaijenhagen, R., Kok, F. J., & Verhoef, H. Efficacy of iron-fortified whole maize flour on iron status of schoolchildren in Kenya: a randomised controlled trial. *The Lancet*, 369(9575), 1799-1806.

Andersen, C. T., Reynolds, S. A., Behrman, J. R., Crookston, B. T., Dearden, K. A., Escobal, J., Mani, S., Sánchez, A., Stein, A. D., & Fernald, L. C. (2015). Participation in the Juntos Conditional Cash Transfer Program in Peru Is Associated with Changes in Child Anthropometric Status but Not Language Development or School Achievement. *The Journal of Nutrition*, 145(10), 2396-2405.

Andersson, C., Mekonnen, A., & Stage, J. (2011). Impacts of the Productive Safety Net Program in Ethiopia on livestock and tree holdings of rural households. *Journal of Development Economics*, 94(1), 119-126.

Andrade, S., Lachat, C., Ochoa-Aviles, A., Verstraeten, R., Huybregts, L., Roberfroid, D., Andrade, D., Camp, J. V., Rojas, R., Donoso, S., Cardon, G., & Kolsteren, P. (2014). A school-based intervention improves physical fitness in Ecuadorian adolescents: a

cluster-randomized controlled trial. International *Journal of Behavioral Nutrition and Physical Activity*, 11(1), 153.

Andrade, S., Verloigne, M., Cardon, G., Kolsteren, P., Ochoa-Avilés, A., Verstraeten, R., Donoso, S., & Lachat, C. (2015). School-based intervention on healthy behaviour among Ecuadorian adolescents: effect of a cluster-randomized controlled trial on screen-time. *BMC Public Health*, 15(1), 942.

Angelucci, M. (2008). Love on the rocks: Domestic violence and alcohol abuse in rural Mexico. *The BE Journal of Economic Analysis & Policy*, 8(1).

Angelucci, M., Attanasio, O., & Di Maro, V. (2012). The Impact of Oportunidades on Consumption, Savings and Transfers. *Fiscal Studies*, 33(3), 305-334.

Angelucci, M., & Attanasio, O. (2009). *Oportunidades*: program effect on consumption, low participation, and methodological issues. *Economic Development and Cultural Change*, 57(3), 479-506.

Aninanya, G. A., Debpuur, C. Y., Awine, T., Williams, J. E., Hodgson, A., & Howard, N. (2015). Effects of an Adolescent Sexual and Reproductive Health Intervention on Health Service Usage by Young People in Northern Ghana: A Community-Randomised Trial. *PLOS ONE*, 10(4), e0125267.

Antunes, M. C., Stall, R. D., Paiva, V., Peres, C. A., Paul, J., Hudes, M., & Hearst, N. (1997). Evaluating an AIDS sexual risk reduction program for young adults in public night schools in Sao Paulo, Brazil. *AIDS*, 11 Suppl 1, S121-127.

Aplasca, M. R., Siegel, D., Mandel, J. S., Santana-Arciaga, R. T., Paul, J., Hudes, E. S., Monzon, O. T., & Hearst, N. (1995). Results of a model AIDS prevention program for high school students in the Philippines. *AIDS*, 9 Suppl 1, S7-13.

Arcand, J. L., & Wouabe, E. D. (2010). Teacher training and HIV/AIDS prevention in West Africa: regression discontinuity design evidence from the Cameroon. *Health Econ*, 19 Suppl, 36-54.

Arcanjo, F. P., Arcanjo, C. C., Amancio, O. M. S., Braga, J. A. P., & Leite, Á. J. M. (2011). Weekly Iron Supplementation for the Prevention of Anemia in Pre-school Children: A Randomized, Double-blind, Placebo-controlled Trial. *Journal of Tropical Pediatrics*, 57(6), 433-438.

Arcanjo, F. P., Pinto, V. P., Coelho, M. R., Amancio, O. M., & Magalhaes, S. M. (2008). Anemia reduction in preschool children with the addition of low doses of iron to school meals. *J Trop Pediatr*, 54(4), 243-247.

Arifeen, S. E., Hoque, D. M., Akter, T., Rahman, M., Hoque, M. E., Begum, K., Chowdhury, E. K., Khan, R., Blum, L. S., Ahmed, S., Hossain, M. A., Siddik, A., Begum, N., Sadeq-ur Rahman, Q., Haque, T. M., Billah, S. M., Islam, M., Rumi, R. A., Law, E., Al-Helal, Z. A., Baqui, A. H., Schellenberg, J., Adam, T., Moulton, L. H., Habicht, J. P.,

Scherpbier, R. W., Victora, C. G., Bryce, J., & Black, R. E. (2009). Effect of the Integrated Management of Childhood Illness strategy on childhood mortality and nutrition in a rural area in Bangladesh: a cluster randomised trial. *The Lancet*, 374(9687), 393-403.

Arnold, R., Maticka-Tyndale, E., Tenkorang, E., Holland, D., Gaspard, A., Luginaah, I., & Team, H. R. (2012). Evaluation of school- and community-based HIV prevention interventions with junior secondary school students in Edo State, Nigeria. *Afr J Reprod Health*, 16(2), 103-125.

Arriagada, R. A., Ferraro, P. J., Sills, E. O., Pattanayak, S. K., & Cordero-Sancho, S. (2012). Do payments for environmental services affect forest cover? A farm-level evaluation from Costa Rica. *Land Economics*, 88(2), 382-399.

Arunachalam, N., Tyagi, B. K., Samuel, M., Krishnamoorthi, R., Manavalan, R., Tewari, S. C., Ashokkumar, V., Kroeger, A., Sommerfeld, J., & Petzold, M. (2012). Community-based control of Aedes aegypti by adoption of eco-health methods in Chennai City, India. *Pathog Glob Health*, 106(8), 488-496.

Asadullah, M. N., & Ara, J. (2016). Evaluating the long-run impact of an innovative anti-poverty programme: evidence using household panel data. *Applied Economics*, 48(2), 107-120.

Ashraf, N., Giné, X., & Karlan, D. (2009). Finding missing markets (and a disturbing epilogue): Evidence from an export crop adoption and marketing intervention in Kenya. *American Journal of Agricultural Economics*, 91(4), 973-990.

Assaré, R. K., Tian-Bi, Y. N. T., Yao, P. K., N'Guessan, N. A., Ouattara, M., Yapi, A., Coulibaly, J. T., Meïté, A., Hürlimann, E., Knopp, S., Utzinger, J., & N'Goran, E. K. (2016). Sustaining Control of Schistosomiasis Mansoni in Western Côte d'Ivoire: Results from a SCORE Study, One Year after Initial Praziquantel Administration. *PLOS Neglected Tropical Diseases*, 10(1), e0004329.

Attanasio, O., Fernández, C., Fitzsimons, E. O. A., Grantham-McGregor, S. M., Meghir, C., & Rubio-Codina, M. (2014). Using the infrastructure of a conditional cash transfer program to deliver a scalable integrated early child development program in Colombia: cluster randomized controlled trial. *BMJ*, 349.

Attanasio, O., Fitzsimons, E., Gomez, A., Gutierrez, M. I., Meghir, C., & Mesnard, A. (2010). Children's schooling and work in the presence of a conditional cash transfer program in rural Colombia. *Economic Development and Cultural Change*, 58(2), 181-210.

Attanasio, O. P., Meghir, C., & Santiago, A. (2012). Education choices in Mexico: using a structural model and a randomized experiment to evaluate Progresa. *The Review of Economic Studies*, 79(1), 37-66.

Attanasio, O., & Mesnard, A. (2006). The impact of a conditional cash transfer programme on consumption in Colombia. *Fiscal Studies*, 27(4), 421-442.

Attanasio, O., Pellerano, L., & Reyes, S. P. (2009). Building Trust? Conditional Cash Transfer Programmes and Social Capital. *Fiscal Studies*, 30(2), 139-177.

Attanasio, O., Polania-Reyes, S., & Pellerano, L. (2015). Building social capital: Conditional cash transfers and cooperation. *Journal of Economic Behavior & Organization*, 118(Supplement C), 22-39.

Atwood, K. A., Kennedy, S. B., Shamblen, S., Tegli, J., Garber, S., Fahnbulleh, P. W., Korvah, P. M., Kolubah, M., Mulbah-Kamara, C., & Fulton, S. (2012). Impact of school-based HIV prevention program in post-conflict Liberia. *AIDS Educ Prev*, 24(1), 68-77.

Augsburg, B. (2009). The impact of a dairy intervention in rural India: evidence from realised outcomes and expected returns to investment. *Journal of Development Effectiveness*, 1(2), 147-170.

Austrian, K., & Muthengi, E. (2014). Can economic assets increase girls' risk of sexual harassment? Evaluation results from a social, health and economic asset-building intervention for vulnerable adolescent girls in Uganda. *Children and Youth Services Review*, 47(2), 168-175.

Avitabile, C. (2012). Does Information Improve the Health Behavior of Adults Targeted by a Conditional Transfer Program? *Journal of Human Resources*, 47(3), 785-825.

Avula, R., Frongillo, E. A., Arabi, M., Sharma, S., & Schultink, W. (2011). Enhancements to nutrition program in Indian integrated child development services increased growth and energy intake of children. *The Journal of Nutrition*, 141(4), 680-684.

Awasthi, S., Peto, R., Pande, V. K., Fletcher, R. H., Read, S., & Bundy, D. A. (2008). Effects of deworming on malnourished preschool children in India: an open-labelled, cluster-randomized trial. *PLoS Negl Trop Dis*, 2(4), e223.

Awasthi, S., Peto, R., Read, S., Richards, S. M., Pande, V., Bundy, D., & the DEVTA team. (2013). Population deworming every 6 months with albendazole in 1 million pre-school children in north India: DEVTA, a cluster-randomised trial. *The Lancet*, 381(9876), 1478-1486.

Baird, S. J., Garfein, R. S., McIntosh, C. T., & Ozler, B. (2012). Effect of a cash transfer programme for schooling on prevalence of HIV and herpes simplex type 2 in Malawi: a cluster randomised trial. *The Lancet*, 379(9823), 1320-1329.

Baird, S., Chirwa, E., McIntosh, C., & Ozler, B. (2010). The short-term impacts of a schooling conditional cash transfer program on the sexual behavior of young women. *Health Econ*, 19, 55-68.

Baird, S., De Hoop, J., & Özler, B. (2013). Income shocks and adolescent mental health. *Journal of Human Resources*, 48(2), 370-403.

Baird, S., McIntosh, C., & Özler, B. (2011). Cash or condition? Evidence from a cash transfer experiment. *The Quarterly Journal of Economics*, qjr032.

Baker-Henningham, H., Powell, C., Walker, S., & Grantham-McGregor, S. (2005). The effect of early stimulation on maternal depression: a cluster randomised controlled trial. *Archives of Disease in Childhood*, 90(12), 1230–1234.

Balaji, M., Andrews, T., Andrew, G., & Patel, V. (2011). The acceptability, feasibility, and effectiveness of a population-based intervention to promote youth health: an exploratory study in Goa, India. *J Adolesc Health*, 48(5), 453-460.

Bali Swain, R., & Varghese, A. (2014). Evaluating the Impact of Training in Self-Help Groups in India. *The European Journal of Development Research*, 26(5), 870-885.

Bandyopadhyay, S., & Tembo, G. (2010). Household consumption and natural resource management around national parks in Zambia. *Journal of Natural Resources Policy Research*, 2(1), 39-55.

Bandyopadhyay, S., Humavindu, M., Shyamsundar, P., & Wang, L. (2009). Benefits to local communities from community conservancies in Namibia: an assessment. *Development Southern Africa*, 26(5), 733-754.

Banerjee, A., Duflo, E., & Hornbeck, R. (2014). Bundling Health Insurance and Microfinance in India: There Cannot be Adverse Selection if There is No Demand. *Am Econ Rev*, 104(5), 291-297.

Baqui, A., Williams, E. K., Rosecrans, A. M., Agrawal, P. K., Ahmed, S., Darmstadt, G. L., Kumar, V., Kiran, U., Panwar, D., Ahuja, R. C., Srivastava, V. K., Black, R. E., & Santosham, M. (2008). Impact of an integrated nutrition and health programme on neonatal mortality in rural northern India. *Bull World Health Organ*, 86(10), 796-804, A.

Barber, S. L. (2010). Mexico's conditional cash transfer programme increases cesarean section rates among the rural poor. *Eur J Public Health*, 20(4), 383-388.

Barber, S. L., & Gertler, P. J. (2008). The impact of Mexico's conditional cash transfer programme, Oportunidades, on birthweight. *Trop Med Int Health*, 13(11), 1405-1414.

Barber, S. L., & Gertler, P. J. (2009). Empowering women to obtain high quality care: evidence from an evaluation of Mexico's conditional cash transfer programme. *Health Policy Plan*, 24(1), 18-25.

Barber, S. L., & Gertler, P. J. (2010). Empowering women: how Mexico's conditional cash transfer programme raised prenatal care quality and birth weight. *Journal of Development Effectiveness*, 2(1), 51-73.

Barbosa, A. L. N. d. H., & Corseuil, C. H. L. (2014). Conditional cash transfer and informality in Brazil. *IZA Journal of Labor & Development*, 3(1), 37.

Barham, T. (2011). A healthier start: the effect of conditional cash transfers on neonatal and infant mortality in rural Mexico. *Journal of Development Economics*, 94(1), 74-85.

Barham, T., & Maluccio, J. A. (2009). Eradicating diseases: The effect of conditional cash transfers on vaccination coverage in rural Nicaragua. *J Health Econ*, 28(3), 611-621.

Barham, T., Macours, K., & Maluccio, J. A. (2013). Boys' Cognitive Skill Formation and Physical Growth: Long-Term Experimental Evidence on Critical Ages for Early Childhood Interventions. *Am Econ Rev*, 103(3), 467-471.

Barrera-Osorio, F., Bertrand, M., Linden, L. L., & Perez-Calle, F. (2011). Improving the design of conditional transfer programs: Evidence from a randomized education experiment in Colombia. *American Economic Journal: Applied Economics*, 167-195.

Barrientos, A., & Villa, J. M. (2015). Antipoverty Transfers and Labour Market Outcomes: Regression Discontinuity Design Findings. *The Journal of Development Studies*, 51(9), 1224-1240.

Barron, I. G., Abdallah, G., & Smith, P. (2013). Randomized control trial of a CBT trauma recovery program in Palestinian schools. *Journal of Loss and Trauma*, 18(4), 306-321.

Bauch, S. C., Sills, E. O., & Pattanayak, S. K. (2014). Have We Managed to Integrate Conservation and Development? ICDP Impacts in the Brazilian Amazon. *World Development*, 64(1), S135-S148.

Bauchet, J., Morduch, J., & Ravi, S. (2015). Failure vs. displacement: Why an innovative anti-poverty program showed no net impact in South India. *Journal of Development Economics*, 116(SC), 1-16.

Beath, A., Fotini, C., & Enikolopov, R. (2013). Empowering Women through Development Aid: Evidence from a Field Experiment in Afghanistan. *American Political Science Review*, 107(3), 540-557.

Beath, A., Fontini, C., & Enikolopov, R. (2015). The National Solidarity Programme: Assessing the Effects of Community-Driven Development in Afghanistan. *International Peacekeeping*, 22(4), 302-320.

Behrman, J. R., & Hoddinott, J. (2005). Programme evaluation with unobserved heterogeneity and selective implementation: The Mexican PROGRESA impact on child nutrition. *Oxford bulletin of economics and statistics*, 67(4), 547-569.

Behrman, J. R., Cheng, Y., & Todd, P. E. (2004). Evaluating preschool programs when length of exposure to the program varies: A nonparametric approach. *Review of Economics and Statistics*, 86(1), 108-132.

Behrman, J. R., Parker, S. W., & Todd, P. E. (2009). Schooling Impacts of Conditional Cash Transfers on Young Children: Evidence from Mexico. *Econ Dev Cult Change*, 57(3), 439-477.

Behrman, J. R., Parker, S. W., & Todd, P. E. (2011). Do conditional cash transfers for schooling generate lasting benefits? A five-year followup of PROGRESA/Oportunidades. *Journal of human resources*, 46(1), 93-122.

Behrman, J. R., Sengupta, P., & Todd, P. (2005). Progressing through PROGRESA: an impact assessment of a school subsidy experiment in rural Mexico. *Economic Development and Cultural Change*, 54(1), 237-275.

Benedetti, F., Ibarrarán, P., & McEwan, P. J. (2016). Do Education and Health Conditions Matter in a Large Cash Transfer? Evidence from a Honduran Experiment. *Economic Development and Cultural Change*, 64(4), 759-793.

Benin, S., Nkonya, E., Okecho, G., Randriamamonjy, J., Kato, E., Lubade, G., & Kyotalimye, M. (2011). Returns to spending on agricultural extension: the case of the National Agricultural Advisory Services (NAADS) program of Uganda. *Agricultural Economics*, 42(2), 249-267.

Berhane, G., O.Gilligan, D., JohnHoddinott, NehaKumar, & SeyoumTaffesse, A. (2014). Can Social Protection Work in Africa? The Impact of Ethiopia’s Productive Safety Net Programme. *Economic Development and Cultural Change*, 63(1), 1-26.

Bezner Kerr, R., Berti, P., & Shumba, L. (2011). Effects of a participatory agriculture and nutrition education project on child growth in northern Malawi. *Public Health Nutrition*, 14(8), 1466-1472.

Bhandari, N., Mazumder, S., Bahl, R., Martines, J., Black, R. E., & Bhan, M. K. (2005). Use of multiple opportunities for improving feeding practices in under-twos within child health programmes. *Health Policy and Planning*, 20(5), 328-336.

Bhandari, N., Mazumder, S., Taneja, S., Sommerfelt, H., Strand, T. A., & Group, I. E. S. (2012). Effect of implementation of Integrated Management of Neonatal and Childhood Illness (IMNCI) programme on neonatal and infant mortality: cluster randomised controlled trial. *BMJ*, 344, e1634.

Bieri, F. A., Gray, D. J., Williams, G. M., Raso, G., Li, Y. S., Yuan, L., He, Y., Li, R. S., Guo, F. Y., Li, S. M., & McManus, D. P. (2013). Health-Education Package to Prevent Worm Infections in Chinese Schoolchildren. *New England Journal of Medicine*, 368(17), 1603-1612.

Biosca, O., Lenton, P., & Mosley, P. (2014). Where is the ‘Plus’ in ‘Credit-Plus’? The Case of Chiapas, Mexico. *The Journal of Development Studies*, 50(12), 1700-1716.

Bobonis, G. J. (2011). The impact of conditional cash transfers on marriage and divorce. *Econ Dev Cult Change*, 59(2), 281-312.

Bobonis, G. J. (2009). Is the Allocation of Resources within the Household Efficient? New Evidence from a Randomized Experiment. *Journal of Political Economy*, 117(3), 453-503.

Bobonis, G. J., Miguel, E., & Puri-Sharma, C. (2006). Anemia and school participation. *Journal of Human Resources*, 41(4), 692-721.

Borraz, F., & González, N. (2009). Impact of the Uruguayan conditional cash transfer program. *Cuadernos de Economía*, 46(134), 243-271.

Borzekowski, D. L., & Henry, H. K. (2010). The impact of *Jalan Sesama* on the educational and healthy development of Indonesian preschool children: An experimental study. *International Journal of Behavioral Development*, 32(2), 169-179.

Bottaro, S. M., & Giugliani, E. R. (2009). Effectiveness of an intervention to improve breastfeeding knowledge and attitudes among fifth-grade children in Brazil. *J Hum Lact*, 25(3), 325-332.

Bowen, A., Ma, H., Ou, J., Billhimer, W., Long, T., Mintz, E., Hoekstra, R. M., & Luby, S. (2007). A cluster-randomized controlled trial evaluating the effect of a handwashing-promotion program in Chinese primary schools. *Am J Trop Med Hyg*, 76(6), 1166-1173.

Braido, L. H., Olinto, P., & Perrone, H. (2012). Gender bias in intrahousehold allocation: Evidence from an unintentional experiment. *Review of Economics and Statistics*, 94(2), 552-565.

Bravo-Ureta, B. E., Almeida, A. N., Solís, D., & Inestroza, A. (2011). The economic impact of MARENA's investments on sustainable agricultural systems in Honduras. *Journal of Agricultural Economics*, 62(2), 429-448.

Burnett, S. M., Weaver, M. R., Mody-Pan, P. N., Thomas, L. A., & Mar, C. M. (2011). Evaluation of an intervention to increase human immunodeficiency virus testing among youth in Manzini, Swaziland: a randomized control trial. *J Adolesc Health*, 48(5), 507-513.

Buttenheim, A. M., Alderman, H., & Friedman, J. (2013). Impact evaluation of school feeding programs in Lao PDR. *Journal of Development Effectiveness*, 3(4), 520-542.

Cabalda, A. B., Tengco, L. W., Solon, J. A., Sarol, J. N., Jr., Rayco-Solon, P., & Solon, F. S. (2009). Efficacy of pandesal baked from wheat flour fortified with iron and vitamin a in improving the iron and anthropometric status of anemic schoolchildren in the Philippines. *J Am Coll Nutr*, 28(5), 591-600.

Cabezon, C., Vigil, P., Rojas, I., Leiva, M. E., Riquelme, R., Aranda, W., & Garcia, C. (2005). Adolescent pregnancy prevention: An abstinence-centered randomized controlled intervention in a Chilean public high school. *J Adolesc Health*, 36(1), 64-69.

Cáceres, C. F., Rosasco, A. M., Mandel, J. S., & Hearst, N. (1994). Evaluating a school-based intervention for STD/AIDS prevention in Peru. *Journal of Adolescent Health*, 15(7), 582-591.

Cai, Y., Hong, H., Shi, R., Ye, X., Xu, G., Li, S., & Shen, L. (2008). Long-term follow-up study on peer-led school-based HIV/AIDS prevention among youths in Shanghai. *Int J STD AIDS*, 19(12), 848-850.

Cao, Z. J., Wang, S. M., & Chen, Y. (2015). A Randomized Trial of Multiple Interventions for Childhood Obesity in China. *American Journal of Preventive Medicine*, 48(5), 552-560.

Caprara, A., De Oliveira Lima, J. W., Rocha Peixoto, A. C., Vasconcelos Motta, C. M., Soares Nobre, J. M., Sommerfeld, J., & Kroeger, A. (2015). Entomological impact and social participation in dengue control: a cluster randomized trial in Fortaleza, Brazil. *Transactions of The Royal Society of Tropical Medicine and Hygiene*, 109(2), 99-105.

Caruso, B. A., Freeman, M. C., Garn, J. V., Dreibelbis, R., Saboori, S., Muga, R., & Rheingans, R. (2014). Assessing the impact of a school-based latrine cleaning and handwashing program on pupil absence in Nyanza Province, Kenya: a cluster-randomized trial. *Tropical Medicine & International Health*, 19(10), 1185-1197.

Castro, M., Sanchez, L., Perez, D., Carbonell, N., Lefevre, P., Vanlerberghe, V., & Van der Stuyft, P. (2012). A community empowerment strategy embedded in a routine dengue vector control programme: a cluster randomised controlled trial. *Trans R Soc Trop Med Hyg*, 106(5), 315-321.

Ceesay, S. M., Prentice, A. M., Cole, T. J., Foord, F., Weaver, L. T., Poskitt, E. M., & Whitehead, R. G. (1997). Effects on birth weight and perinatal mortality of maternal dietary supplements in rural Gambia: 5 year randomised controlled trial. *BMJ*, 315(7111), 786-790.

Cespedes, J., Briceno, G., Farkouh, M. E., Vedanthan, R., Baxter, J., Leal, M., Boffetta, P., Woodward, M., Hunn, M., Dennis, R., & Fuster, V. (2013). Targeting preschool children to promote cardiovascular health: cluster randomized trial. *Am J Med*, 126(1), 27-35 e23.

Chandramohan, D., Owusu-Agyei, S., Carneiro, I., Awine, T., Amponsa-Achiano, K., Mensah, N., Jaffar, S., Baiden, R., Hodgson, A., Binka, F., & Greenwood, B. (2005). Cluster randomised trial of intermittent preventive treatment for malaria in infants in area of high, seasonal transmission in Ghana. *BMJ*, 331(7519), 727-733.

Chatterji, M., Hutchinson, P., Buek, K., Murray, N., Mulenga, Y., & Ventimiglia, T. (2010). Evaluating the impact of community-based interventions on schooling outcomes among orphans and vulnerable children in Lusaka, Zambia. *Vulnerable Children and Youth Studies*, 5(2), 130-141.

Chaudhury, N., & Parajuli, D. (2010). Conditional cash transfers and female schooling: the impact of the female school stipend program on public school enrollments in Punjab, Pakistan. *Applied Economics*, 42(28), 3565-3583.

Chaya, M. S., Nagendra, H., Selvam, S., Kurpad, A., & Srinivasan, K. (2012). Effect of yoga on cognitive abilities in schoolchildren from a socioeconomically disadvantaged background: a randomized controlled study. *J Altern Complement Med*, 18(12), 1161-1167.

Chen, K., Liu, T. Y., Chen, L., Qu, P., & Liu, Y. X. (2008). Effects of vitamin A, vitamin A plus iron and multiple micronutrient-fortified seasoning powder on preschool children in a suburb of Chongqing, *China. J Nutr Sci Vitaminol*, 54(6), 440-447.

Chen, S., Mu, R., & Ravallion, M. (2009). Are there lasting impacts of aid to poor areas? *Journal of Public Economics*, 93(3), 512-528.

Cheung, M., & Perrotta Berlin, M. (2015). The Impact of a Food For Education Program on Schooling in Cambodia. A*sia and the Pacific Studies*, 2(1), 44-57.

Chhabra, R., Springer, C., Leu, C. S., Ghosh, S., Sharma, S. K., & Rapkin, B. (2010). Adaptation of an alcohol and HIV school-based prevention program for teens. *AIDS Behav*, 14 Suppl 1, S177-184.

Chhabra, R., Springer, C., Rapkin, B., & Merchant, Y. (2007). Differences among male/female adolescents participating in a School-based Teenage Education Program (STEP) focusing on HIV prevention in India. *Ethnicity & Disease*, 18(2 Suppl 2), S2-123-127.

Chou, C. P., Li, Y., Unger, J. B., Xia, J., Sun, P., Guo, Q., Shakib, S., Gong, J., Xie, B., Liu, C., Azen, S., Shan, J., Ma, H., Palmer, P., Gallaher, P., & Johnson, C. A. (2006). A randomized intervention of smoking for adolescents in urban Wuhan, China. *Prev Med*, 42(4), 280-285.

Chowa, G. A. N., & Elliott, W. (2011). An asset approach to increasing perceived household economic stability among families in Uganda. *The Journal of Socio-Economics*, 40(1), 81-87.

Clark, N. M., Gong, M., Kaciroti, N., Yu, J., Wu, G., Zeng, Z., & Wu, Z. (2005). A trial of asthma self-management in Beijing schools. *Chronic Illn*, 1(1), 31-38.

Clarke, S. E., Jukes, M. C., Njagi, J. K., Khasakhala, L., Cundill, B., Otido, J., Crudder, C., Estambale, B. B., & Brooker, S. (2008). Effect of intermittent preventive treatment of malaria on health and education in schoolchildren: a cluster-randomised, double-blind, placebo-controlled trial. *The Lancet*, 372(9633), 127-138.

Clements, T., & Milner-Gulland, E. J. (2015). Impact of payments for environmental services and protected areas on local livelihoods and forest conservation in northern Cambodia. *Conservation Biology*, 29(1), 78–87.

Coady, D. P., & Parker, S. W. (2004). Cost-effectiveness analysis of demand-and supply-side education interventions: the case of PROGRESA in Mexico. *Review of Development Economics*, 8(3), 440-451.

Coffman, D. L., Smith, E. A., Flisher, A. J., & Caldwell, L. L. (2011). Effects of HealthWise South Africa on Condom Use Self-efficacy. *Prevention Science*, 12(2), 162-172.

Colin-Ramirez, E., Castillo-Martinez, L., Orea-Tejeda, A., Vergara-Castaneda, A., Keirns-Davis, C., & Villa-Romero, A. (2010). Outcomes of a school-based intervention (RESCATE) to improve physical activity patterns in Mexican children aged 8-10 years. *Health Educ Res*, 25(6), 1042-1049.

Congdon, N., Li, L., Zhang, M., Yang, A., Gao, Y., Griffiths, S., Wu, J., Sharma, A., & Lam, D. S. (2011). Randomized, controlled trial of an educational intervention to promote spectacle use in rural China: the see well to learn well study. *Ophthalmology*, 118(12), 2343-2350.

Cooper, P. J., Chico, M. E., Vaca, M. G., Moncayo, A. L., Bland, J. M., Mafla, E., Sanchez, F., Rodrigues, L. C., Strachan, D. P., & Griffin, G. E. (2006). Effect of albendazole treatments on the prevalence of atopy in children living in communities endemic for geohelminth parasites: a cluster-randomised trial. *The Lancet*, 367(9522), 1598-1603.

Cord, L., & Wodon, Q. (2001). Do agricultural programs in Mexico alleviate poverty?: Evidence from the ejido sector. *Cuadernos de Economía*, 38(114), 239-256.

Correa, J. C., Pinto, D., Salas, L. A., Camacho, J. C., Rondon, M., & Quintero, J. (2012). A cluster-randomized controlled trial of handrubs for prevention of infectious diseases among children in Colombia. *Rev Panam Salud Publica*, 31(6), 476-484.

Covarrubias, K., Davis, B., & Winters, P. (2012). From protection to production: productive impacts of the Malawi social cash transfer scheme. *Journal of Development Effectiveness*, 4(1), 50-77.

Cuetoa, S., & Chinenb, M. (2008). Educational Impact of a School Breakfast Programme in Rural Peru. *International Journal of Educational Development*, 28(2), 132-148.

Cui, Z., Shah, S., Yan, L., Pan, Y., Gao, A., Shi, X., Wu, Y., & Dibley, M. J. (2012). Effect of a school-based peer education intervention on physical activity and sedentary behaviour in Chinese adolescents: a pilot study. *BMJ Open*, 2(3).

Cunha, D. B., Souza, B. d. S. N. d., Pereira, R. A., & Sichieri, R. (2013). Effectiveness of a Randomized School-Based Intervention Involving Families and Teachers to Prevent Excessive Weight Gain among Adolescents in Brazil. *PLOS ONE*, 8(2), e57498.

Cupp, P. K., Zimmerman, R. S., Bhana, A., Feist-Price, S., Dekhtyar, O., Karnell, A., & Ramsoomar, L. (2008). Combining and adapting American school-based alcohol and HIV prevention programmes in South Africa: The HAPS project. *Vulnerable Children and Youth Studies*, 3(2), 134-142.

D’Elia, V. V., & Navarro, A. I. (2013). Universal Child Allowance and School Delay of Children In Argentina. *Revista de Análisis Económico – Economic Analysis Review*, 28(2), 63-90.

Dalton, A., Wolmarans, P., Witthuhn, R. C., van Stuijvenberg, M. E., Swanevelder, S. A., & Smuts, C. M. (2009). A randomised control trial in schoolchildren showed improvement in cognitive function after consuming a bread spread, containing fish flour from a marine source. *Prostaglandins Leukot Essent Fatty Acids*, 80(2-3), 143-149.

Dangour, A. D., Albala, C., Allen, E., Grundy, E., Walker, D., Aedo, C., Sanchez, H., Fletcher, O., Elbourne, D., & Uauy, R. (2011). Effect of a Nutrition Supplement and Physical Activity Program on Pneumonia and Walking Capacity in Chilean Older People: A Factorial Cluster Randomized Trial. *PLOS Med*, 8(4), e1001023.

Darney, B. G., Weaver, M. R., Sosa-Rubi, S. G., Walker, D., Servan-Mori, E., Prager, S., & Gakidou, E. (2013). The Oportunidades Conditional Cash Transfer Program: Effects on Pregnancy and Contraceptive Use among Young Rural Women in Mexico. *International Perspectives on Sexual and Reproductive Health*, 39(4), 205–214.

Davey, C., Aiken, A. M., Hayes, R. J., & Hargreaves, J. R. (2015). Re-analysis of health and educational impacts of a school-based deworming programme in western Kenya: a statistical replication of a cluster quasi-randomized stepped-wedge trial. *International Journal of Epidemiology*, 44(5), 1581-1592.

de Barros, M. V., Nahas, M. V., Hallal, P. C., de Farias Junior, J. C., Florindo, A. A., & Honda de Barros, S. S. (2009). Effectiveness of a school-based intervention on physical activity for high school students in Brazil: the Saude na Boa project. *J Phys Act Health*, 6(2), 163-169.

de Brauw, A., Eozenou, P., & Moursi, M. (2015). Programme Participation Intensity and Children’s Nutritional Status: Evidence from a Randomised Control Trial in Mozambique. *The Journal of Development Studies*, 51(8), 996-1015.

de Brauw, A., Gilligan, D. O., Hoddinott, J., & Roy, S. (2014). The impact of Bolsa Família on women’s decision-making power. *World Development*, 59, 487-504.

de Brauw, A., Gilligan, D., Hoddinott, J., & Roy, S. (2015). Bolsa Família and Household Labor Supply. *Economic Development and Cultural Change*, 63(3), 423-457.

de Brauw, A., Gilligan, D. O., Hoddinott, J., & Roy, S. (2015). The Impact of Bolsa Familia on Schooling. *World Development*, 70, 303-316.

de Brauw, A., & Hoddinott, J. (2011). Must conditional cash transfer programs be conditioned to be effective? The impact of conditioning transfers on school enrollment in Mexico. *Journal of Development Economics*, 96(2), 359-370.

de Farias, I. A., de Araujo Souza, G. C., & Ferreira, M. A. (2009). A health education program for Brazilian public schoolchildren: the effects on dental health practice and oral health awareness. *J Public Health Dent*, 69(4), 225-230.

de Janvry, A, Finan, F., & Sadoulet, E. (2012). Local Electoral Incentives and Decentralized Program Performance. *The Review of Economics and Statistics*, 94(3), 672-685.

de Janvry, A., Finan, F., Sadoulet, E., & Vakis, R. (2006). Can conditional cash transfer programs serve as safety nets in keeping children at school and from working when exposed to shocks? *Journal of Development Economics*, 79(2), 349-373.

de la Cruz, N., Crookston, B., Gray, B., Alder, S., & Dearden, K. (2009). Microfinance against malaria: impact of Freedom from Hunger's malaria education when delivered by rural banks in Ghana. *Trans R Soc Trop Med Hyg*, 103(12), 1229-1236.

De La O, A. L. (2013). Do conditional cash transfers affect electoral behavior? Evidence from a randomized experiment in Mexico. *American Journal of Political Science*, 57(1), 1-14.

de Villiers, M., & van den Berg, H. (2012). The implementation and evaluation of a resiliency programme for children*. South African Journal of Psychology*, 42(1), 93-102.

de Walque, D., Dow, W. H., Nathan, R., Abdul, R., Abilahi, F., Gong, E., Isdahl, Z., Jamison, J., Jullu, B., Krishnan, S., Majura, A., Miguel, E., Moncada, J., Mtenga, S., Mwanyangala, M. A., Packel, L., Schachter, J., Shirima, K., & Medlin, C. A. (2012). Incentivising safe sex: a randomised trial of conditional cash transfers for HIV and sexually transmitted infection prevention in rural Tanzania. *BMJ Open*, 2(1), e000747.

Dehdari, T., Rahimi, T., Aryaeian, N., & Gohari, M. (2014). Effect of nutrition education intervention based on Pender's Health Promotion Model in improving the frequency and nutrient intake of breakfast consumption among female Iranian students. *Public Health Nutrition*, 17(3), 657-666.

Del Carpio, X. V., Loayza, N. V., & Wada, T. (2016). The Impact of Conditional Cash Transfers on the Amount and Type of Child Labor. *World Development*, 80, 33-47.

Démurger, S., & Wan, H. (2012). Payments for ecological restoration and internal migration in China: the sloping land conversion program in Ningxia. *IZA Journal of Migration*, 1(1), 10.

Denison, J. A., Tsui, S., Bratt, J., Torpey, K., Weaver, M. A., & Kabaso, M. (2012). Do peer educators make a difference? An evaluation of a youth-led HIV prevention model in Zambian Schools. *Health Education Research*, 27(2), 237-247.

Desai, J., & Tarozzi, A. (2011). Microcredit, family planning programs, and contraceptive behavior: evidence from a field experiment in Ethiopia. *Demography*, 48(2), 749-782.

Diab, M., Punamäki, R. L., Palosaari, E., & Qouta, S. R. (2014). Can Psychosocial Intervention Improve Peer and Sibling Relations Among War-affected Children? Impact and Mediating Analyses in a Randomized Controlled Trial. *Social Development*, 23(2), 215-231.

Dick, J., Clarke, M., van Zyl, H., & Daniels, K. (2007). Primary health care nurses implement and evaluate a community outreach approach to health care in the South African agricultural sector. *Int Nurs Rev*, 54(4), 383-390.

Djebbari, H., & Smith, J. (2008). Heterogeneous impacts in PROGRESA. *Journal of Econometrics*, 145(1), 64-80.

Donegan, S., Maluccio, J. A., Myers, C. K., Menon, P., Ruel, M. T., & Habicht, J. P. (2010). Two food-assisted maternal and child health nutrition programs helped mitigate the impact of economic hardship on child stunting in Haiti. *J Nutr*, 140(6) 1139-1145.

Doyle, A. M., Ross, D. A., Maganja, K., Baisley, K., Masesa, C., Andreasen, A., Plummer, M. L., Obasi, A. I., Weiss, H. A., Kapiga, S., Watson-Jones, D., Changalucha, J., Hayes, R. J., & Group, M. k. V. T. S. (2010). Long-term biological and behavioural impact of an adolescent sexual health intervention in Tanzania: follow-up survey of the community-based MEMA kwa Vijana Trial. *PLoS Med*, 7(6), e1000287.

Doyle, A. M., Weiss, H. A., Maganja, K., Kapiga, S., McCormack, S., Watson-Jones, D., Changalucha, J., Hayes, R. J., & Ross, D. A. (2011). The long-term impact of the MEMA kwa Vijana adolescent sexual and reproductive health intervention: effect of dose and time since intervention exposure. *PLoS One*, 6(9), e24866.

Dreibelbis, R., Freeman, M. C., Greene, L. E., Saboori, S., & Rheingans, R. (2014). The Impact of School Water, Sanitation, and Hygiene Interventions on the Health of Younger Siblings of Pupils: a Cluster-Randomized Trial in Kenya. *American Journal of Public Health*, 104(1), e91–e97.

Duarte, G. B., Sampaio, B., & Sampaio, Y. (2009). Programa Bolsa Família: impacto das transferências sobre os gastos com alimentos em famílias rurais. *Revista de Economia e Sociologia Rural*, 47(4), 903-918.

Dubois, P., & Rubio-Codina, M. (2012). Child care provision: Semiparametric evidence from a randomized experiment in Mexico. *Annals of Economics and Statistics*, 105/106 155-184.

Dubois, P., de Janvry, A., & Sadoulet, E. (2012). Effects on school enrollment and performance of a conditional transfers program in Mexico. *Journal of Labor Economics*, 30(3), 555-589.

Duflo, E., Dupas, P., & Kremer, M. (2015). Education, HIV and Early Fertility: Experimental Evidence from Kenya. *American Economic Review*, 105(9), 2757-2797.

Dunbar, M. S., Kang Dufour, M. S., Lambdin, B., Mudekunye-Mahaka, I., Nhamo, D., & Padian, N. S. (2014). The SHAZ! project: results from a pilot randomized trial of a structural intervention to prevent HIV among adolescent women in Zimbabwe. *PLoS One*, 9(11), e113621.

Dupas, P. (2011). Do teenagers respond to HIV risk information? Evidence from a field experiment in Kenya, *American Economic Journal: Applied Economics*, 3(1), 1-34.

Ebenezer, R., Gunawardena, K., Kumarendran, B., Pathmeswaran, A., Jukes, M. C. H., Drake, L. J., & de Silva, N. (2013). Cluster-randomised trial of the impact of school-based deworming and iron supplementation on the cognitive abilities of schoolchildren in Sri Lanka's plantation sector. *Trop Med Int Health*, 18, 942–951.

Edmonds, E. V. & Maheshwor, S. (2014). You get what you pay for: Schooling incentives and child labor. *Journal of Development Economics*, 111, 196-211.

Egbi, G., Ayi, I., Saalia, F. K., Zotor, F., Adom, T., Harrison, E., Ahorlu, C. K., & Steiner-Asiedu, M. (2015). Impact of Cowpea-Based Food Containing Fish Meal Served With Vitamin C–Rich Drink on Iron Stores and Hemoglobin Concentrations in Ghanaian Schoolchildren in a Malaria Endemic Area. *Food and Nutrition Bulletin*, 36(3), 264-275.

El Ansari, W., El Ashker, S., & Moseley, L. (2010). Associations between physical activity and health parameters in adolescent pupils in Egypt. *International Journal of Environmental Research and Public Health*, 7(4), 1649-1669.

Emran, M. S., Robano, V., & Smith, S. C. (2014). Assessing the Frontiers of Ultrapoverty Reduction: Evidence from Challenging the Frontiers of Poverty Reduction/Targeting the Ultra-poor, an Innovative Program in Bangladesh. *Economic Development and Cultural Change*, 62(2), 339-380.

Esere, M. O. (2008). Effect of Sex Education Programme on at-risk sexual behaviour of school-going adolescents in Ilorin, Nigeria. *Afr Health Sci*, 8(2), 120-125.

Falavigna, A., Teles, A. R., Velho, M. C., Medeiros, G. S., Canabarro, C. T., de Braga, G. L., Barazzetti, D. O., Vedana, V. M., & Kleber, F. D. (2012). Impact of an injury prevention program on teenagers' knowledge and attitudes: results of the Pense Bem-Caxias do Sul Project. *J Neurosurg Pediatr*, 9(5), 562-568.

Fearon, J., Humphreys, M., & Weinstein, J. (2009). Can development aid contribute to social cohesion after civil war? Evidence from a field experiment in post-conflict Liberia. *American Economic Review*, 99(2), 287-291.

Feldman, B. S., Zaslavsky, A. M., Ezzati, M., Peterson, K. E., & Mitchell, M. (2009). Contraceptive use, birth spacing, and autonomy: an analysis of the Oportunidades program in rural Mexico. *Stud Fam Plann*, 40(1), 51-62.

Fenn, B., Bulti, A. T., Nduna, T., Duffield, A., & Watson, F. (2012). An evaluation of an operations research project to reduce childhood stunting in a food-insecure area in Ethiopia. *Public Health Nutrition*, 15(9), 1746-1754.

Fernald, L. C., & Gunnar, M. R. (2009). Poverty-alleviation program participation and salivary cortisol in very low-income children. *Soc Sci Med*, 68(12), 2180-2189.

Fernald, L. C., Gertler, P. J., & Neufeld, L. M. (2008). Role of cash in conditional cash transfer programmes for child health, growth, and development: an analysis of Mexico's *Oportunidades*. *The Lancet*, 371(9615), 828-837.

Fernald, L. C., Gertler, P. J., & Neufeld, L. M. (2009). 10-year effect of Oportunidades, Mexico's conditional cash transfer programme, on child growth, cognition, language, and behaviour: a longitudinal follow-up study. *The Lancet*, 374(9706), 1997-2005.

Fernald, L. C., Hou, X., & Gertler, P. J. (2008). *Oportunidades* program participation and body mass index, blood pressure, and self-reported health in Mexican adults. *Prev Chronic Dis*, 5(3), A81.

Fernando, D., de Silva, D., Carter, R., Mendis, K. N., & Wickremasinghe, R. (2006). A randomized, double-blind, placebo-controlled, clinical trial of the impact of malaria prevention on the educational attainment of school children. *Am J Trop Med Hyg*, 74(3), 386-393.

Ferrario, M. N. (2014). The impacts on family consumption of the Bolsa família subsidy programme. *Cepal Review*, 112.

Filmer, D., & Schady, N. (2008). Getting girls into school: evidence from a scholarship program in Cambodia. *Economic Development and Cultural Change*, 56(3), 581-617.

Filmer, D., & Schady, N. (2011). Does more cash in conditional cash transfer programs always lead to larger impacts on school attendance? *Journal of Development Economics*, 96(1), 150-157.

Filteau, S., PrayGod, G., Kasonka, L., Woodd, S., Rehman, A. M., Chisenga, M., Siame, J., Koethe, J. R., Changalucha, J., Michael, D., Kidola, J., Manno, D., Larke, N., Yilma, D., Heimburger, D. C., Friis, H., & Kelly, P. (2015). Effects on mortality of a nutritional intervention for malnourished HIV-infected adults referred for antiretroviral therapy: a randomised controlled trial. *BMC Medicine*, 13(1), 17.

Fink, G., & Masiye, F. (2012). Assessing the impact of scaling-up bednet coverage through agricultural loan programmes: evidence from a cluster randomised controlled trial in Katete, Zambia. *Trans R Soc Trop Med Hyg*, 106(11), 660-667.

Flax, V. L., Negerie, M., Ibrahim, A. U., Leatherman, S., Daza, E. J., & Bentley, M. E. (2014). Integrating Group Counseling, Cell Phone Messaging, and Participant-Generated Songs and Dramas into a Microcredit Program Increases Nigerian Women’s Adherence

to International Breastfeeding Recommendations. *The Journal of Nutrition*, 144(7), 1120-1124.

Fotu, K. F., Millar, L., Mavoa, H., Kremer, P., Moodie, M., Snowdon, W., Utter, J., Vivili, P., Schultz, J. T., Malakellis, M., McCabe, M. P., Roberts, G., & Swinburn, B. A. (2011). Outcome results for the Ma'alahi Youth Project, a Tongan community-based obesity prevention programme for adolescents. *Obesity Reviews*, 12, 41-50.

Francis, M., Nichols, S. S., & Dalrymple, N. (2010). The effects of a school-based intervention programme on dietary intakes and physical activity among primary-school children in Trinidad and Tobago. *Public Health Nutr*, 13(5), 738-747.

Frazao, P. (2011). Effectiveness of the bucco-lingual technique within a school-based supervised toothbrushing program on preventing caries: a randomized controlled trial. *BMC Oral Health*, 11, 11.

Freeman, M. C., & Clasen, T. (2011). Assessing the Impact of a School-based Safe Water Intervention on Household Adoption of Point-of-Use Water Treatment Practices in Southern India. *The American Journal of Tropical Medicine and Hygiene*, 84(3), 370–378.

Freeman, M. C., Clasen, T., Brooker, S. J., Akoko, D. O., & Rheingans, R. (2013). The Impact of a School-Based Hygiene, Water Quality and Sanitation Intervention on Soil-Transmitted Helminth Reinfection: A Cluster-Randomized Trial. *The American Journal of Tropical Medicine and Hygiene*, 89(5), 875–883.

Freeman, M. C., Clasen, T., Dreibelbis, R., Saboori, S., Greene, L. E., Brumback, B., Muga, R., & Rheingans, R. (2014). The impact of a school-based water supply and treatment, hygiene, and sanitation programme on pupil diarrhoea: a cluster-randomized trial. *Epidemiol Infect*, 142(2), 340-351.

Freeman, M. C., Greene, L. E., Dreibelbis, R., Saboori, S., Muga, R., Brumback, B., & Rheingans, R. (2012). Assessing the impact of a school-based water treatment, hygiene and sanitation programme on pupil absence in Nyanza Province, Kenya: a cluster-randomized trial. *Trop Med Int Health*, 17(3), 380-391.

Friis, H., Mwaniki, D., Omondi, B., Muniu, E., Thiong'o, F., Ouma, J., Magnussen, P., Geissler, P. W., & Fleischer Michaelsen, K. (2003). Effects on haemoglobin of multi-micronutrient supplementation and multi-helminth chemotherapy: a randomized, controlled trial in Kenyan school children. *European Journal of Clinical Nutrition*, 57, 573-579.

Friis, H., Ndhlovu, P., Mduluza, T., Kaondera, K., Sandstrom, B., Michaelsen, K. F., Vennervald, B. J., & Christensen, N. O. (1997). The impact of zinc supplementation on Schistosoma mansoni reinfection rate and intensities: a randomized, controlled trial among rural Zimbabwean schoolchildren. *Eur J Clin Nutr*, 51(1), 33-37.

Galasso, E. (2011). Alleviating extreme poverty in Chile: the short term effects of Chile Solidario. *Estudios de Economía*, 38(1), 101-127.

Galiani, S., Gertler, P., Ajzenman, N., & Orsola-Vidal, A. (2016). Promoting Handwashing Behavior: The Effects of Large-scale Community and School-level Interventions. *Health Econ*, 25, 1545–1559.

Galiani, S., & McEwan, P. J. (2013). The heterogeneous impact of conditional cash transfers. *Journal of Public Economics*, 103, 85-96.

Garcia, S., & Hill, J. (2010). Impact of conditional cash transfers on children's school achievement: evidence from Colombia. *Journal of Development Effectiveness*, 2(1), 117-137.

Garn, J. V., Greene, L. E., Dreibelbis, R., Saboori, S., Rheingans, R. D., & Freeman, M. C. (2013). A cluster-randomized trial assessing the impact of school water, sanitation and hygiene improvements on pupil enrolment and gender parity in enrolment. *Journal of Water, Sanitation and Hygiene for Development*, 3(4), 592-601.

Gaurav, S., Cole, S., & Tobacman, J. (2011). Marketing complex financial products in emerging markets: Evidence from rainfall insurance in India. *Journal of Marketing Research*, 48(SPL), S150-S162.

Gebre, T., Ayele, B., Zerihun, M., House, J. I., Stoller, N. E., Zhou, Z., Ray, K. J., Gaynor, B. D., Porco, T. C., Emerson, P. M., Lietman, T. M., & Keenan, J. D. (2011). Latrine promotion for trachoma: assessment of mortality from a cluster-randomized trial in Ethiopia. *Am J Trop Med Hyg*, 85(3), 518-523.

Gebreheiwot, T., & van der Veen, A. (2015). Estimating the impact of a food security program by propensity-score matching. *Journal of Development and Agricultural Economics*, 7, 38-47.

Gee, K. A. (2010). Reducing child labour through conditional cash transfers: evidence from Nicaragua's Red de Protección social. *Development Policy Review*, 28(6), 711-732.

Gelo, D., & Koch, S. F. (2014). The Impact of Common Property Right Forestry: Evidence from Ethiopian Villages. *World Development*, 64, 395-406.

Gertler, P. (2004). Do Conditional Cash Transfers Improve Child Health? Evidence from PROGRESA's Control Randomized Experiment. *The American Economic Review*, 94(2), 336-341.

Gertler, P. J., Martinez, S. W., & Rubio-Codina, M. (2012). Investing cash transfers to raise long-term living standards. *American Economic Journal: Applied Economics*, 4(1), 164-192.

Gilgen, D. D., Mascie-Taylor, C. G., & Rosetta, L. L. (2001). Intestinal helminth infections, anaemia and labour productivity of female tea pluckers in Bangladesh. *Trop Med Int Health*, 6(6), 449-457.

Gilgen, D., & Mascie-Taylor, C. (2001). The effect of anthelmintic treatment on helminth infection and anaemia. *Parasitology*, 122(01), 105-110.

Gilligan, D. O., & Hoddinott, J. (2007), Is There Persistence in the Impact of Emergency Food Aid? Evidence on Consumption, Food Security, and Assets in Rural Ethiopia. *American Journal of Agricultural Economics*, 89(2), 225-242.

Gilligan, D. O., Hoddinott, J., & Taffesse, A. S. (2009). The impact of Ethiopia's Productive Safety Net Programme and its linkages. *The Journal of Development Studies*, 45(10), 1684-1706.

Giné, X., & Yang, D. (2009). Insurance, credit, and technology adoption: Field experimental evidence from Malawi. *Journal of Development Economics*, 89(1), 1-11.

Giné, X., Karlan, D., & Zinman, J. (2010). Put your money where your butt is: a commitment contract for smoking cessation. *American Economic Journal: Applied Economics*, 2, 213-235.

Gitter, S. R., & Barham, B. L. (2008). Women's power, conditional cash transfers, and schooling in Nicaragua. *World Bank Econ Rev*, 22(2), 271-290.

Gitter, S. R., & Barham, B. L. (2009). Conditional cash transfers, shocks, and school enrolment in Nicaragua. *The Journal of Development Studies*, 45(10), 1747-1767.

Givaudan, M., Leenen, I., Van De Vijver, F. J., Poortinga, Y. H., & Pick, S. (2008). Longitudinal study of a school based HIV/AIDS early prevention program for Mexican adolescents. *Psychology, Health and Medicine*, 13(1), 98-110.

Glewwe, P., & Kassouf, A. L. (2012). The impact of the Bolsa Escola/Familia conditional cash transfer program on enrollment, dropout rates and grade promotion in Brazil. *Journal of Development Economics*, 97(2), 505-517.

Glewwe, P., Park, A., & Zhao, M. (2016). A better vision for development: Eyeglasses and academic performance in rural primary schools in China. *Journal of Development Economics*, 122(Supplement C), 170-182.

Goldhaber-Fiebert, J. D., Goldhaber-Fiebert, S. N., Tristan, M. L., & Nathan, D. M. (2003). Randomized controlled community-based nutrition and exercise intervention improves glycemia and cardiovascular risk factors in type 2 diabetic patients in rural Costa Rica. *Diabetes Care*, 26(1), 24-29.

Gordon, J. S., Staples, J. K., Blyta, A., & Bytyqi, M. (2004). Treatment of posttraumatic stress disorder in postwar Kosovo high school students using mind-body skills groups: a pilot study. *J Trauma Stress*, 17(2), 143-147.

Gordoncillo, P. U. (2012). The economic effects of the comprehensive agrarian reform program in the Philippines. *Journal of ISSAAS (International Society for Southeast Asian Agricultural Sciences)*, 18(1), 76-86.

Gowani, S., Yousafzai, A. K., Armstrong, R., & Bhutta, Z. A. (2014). Cost effectiveness of responsive stimulation and nutrition interventions on early child development outcomes in Pakistan. *Annals of the New York Academy of Sciences*, 1308(1), 149-161.

Graves, J. M., Daniell, W. E., Harris, J. R., Obure, A. F. X. O., & Quick, R. (2012). Enhancing a Safe Water Intervention with Student-Created Visual AIDS to Promote Handwashing Behavior in Kenyan Primary Schools. *International Quarterly of Community Health Education*, 32(4), 307-323.

Greene, L. E., Freeman, M. C., Akoko, D., Saboori, S., Moe, C., & Rheingans, R. (2012). Impact of a school-based hygiene promotion and sanitation intervention on pupil hand contamination in Western Kenya: a cluster randomized trial. *Am J Trop Med Hyg*, 87(3), 385-393.

Grigorenko, E. L., Jarvin, L., Kaani, B., Kapungulya, P. P., Kwiatkowski, J., & Sternberg, R. J. (2007). Risk factors and resilience in the developing world: One of many lessons to learn. *Development and Psychopathology*, 19(3), 747-765.

Gultiano, S. A., & King, E. M. (2006). A Better Start in Life: Evaluation Results from an Early Childhood Development Program. *Philippine Journal of Development*, 33(1and2), 101-128.

Guo, R., He, Q., Shi, J., Gong, J., Wang, H., & Wang, Z. (2010). Short-term impact of cognition-motivation-emotional intelligence-resistance skills program on drug use prevention for school students in Wuhan, China. *J Huazhong Univ Sci Technolog Med Sci*, 30(6), 720-725.

Habib-Mourad, C., Ghandour, L. A., Moore, H. J., Nabhani-Zeidan, M., Adetayo, K., Hwalla, N., & Summerbell, C. (2014). Promoting healthy eating and physical activity among school children: findings from Health-E-PALS, the first pilot intervention from Lebanon. *BMC Public Health*, 14, 940.

Haldar, D., Chatterjee, T., Sarkar, A. P., Das, S. K., & Mallik, S. (2011). A study on the role of parental involvement in control of nutritional anemia among children of free primary schools in a rural area of West Bengal. *Indian J Public Health*, 55(4), 332-335.

Hallfors, D., Cho, H., Rusakaniko, S., Iritani, B., Mapfumo, J., & Halpern, C. (2011). Supporting adolescent orphan girls to stay in school as HIV risk prevention: evidence from a randomized controlled trial in Zimbabwe. *Am J Public Health*, 101(6), 1082-1088.

Halliday, K. E., Okello, G., Turner, E. L., Njagi, K., McHaro, C., Kengo, J., Allen, E., Dubeck, M. M., Jukes, M. C. H., & Brooker, S. J. (2014). Impact of Intermittent Screening and Treatment for Malaria among School Children in Kenya: A Cluster Randomised Trial. *PLOS Medicine*, 11(1), e1001594.

Hamad, R., Fernald, L., & Karlan, D. S. (2011). Health education for microcredit clients in Peru: a randomized controlled trial. *BMC Public Health*, 11(1), 51.

Hamadani, J. D., Huda, S. N., Khatun, F., & Grantham-McGregor, S. M. (2006). Psychosocial Stimulation Improves the Development of Undernourished Children in Rural Bangladesh. *The Journal of Nutrition*, 136(10), 2645-2652.

Hamazaki, K., Syafruddin, D., Tunru, I. S., Azwir, M. F., Asih, P. B., Sawazaki, S., & Hamazaki, T. (2008). The effects of docosahexaenoic acid-rich fish oil on behavior, school attendance rate and malaria infection in school children--a double-blind, randomized, placebo-controlled trial in Lampung, Indonesia. *Asia Pac J Clin Nutr*, 17(2), 258-263.

Haque, R., Ahmed, T., Wahed, M. A., Mondal, D., Rahman, A. S. M. H., & Albert, M. J. (2010). Low-dose β-carotene Supplementation and Deworming Improve Serum Vitamin A and β-carotene Concentrations in Preschool Children of Bangladesh. *Journal of Health, Population, and Nutrition*, 28(3), 230–237.

Harris, J. R., Patel, M. K., Juliao, P., Suchdev, P. S., Ruth, L. J., Were, V., Ochieng, C., Faith, S. H., Kola, S., & Otieno, R. (2012). Addressing inequities in access to health products through the use of social marketing, community mobilization, and local entrepreneurs in rural Western Kenya. *International Journal of Population Research*, 2012.

Harvey, B., Stuart, J., & Swan, T. (2000). Evaluation of a drama-in-education programme to increase AIDS awareness in South African high schools: a randomized community intervention trial. *Int J STD AIDS*, 11(2), 105-111.

He, F. J., Wu, Y., Feng, X. X., Ma, J., Ma, Y., Wang, H., Zhang, J., Yuan, J., Lin, C. P., Nowson, C., & MacGregor, G. A. (2015). School based education programme to reduce salt intake in children and their families (School-EduSalt): cluster randomised controlled trial. *BMJ: British Medical Journal*, 350.

Hebbal, M., & Nagarajappa, R. (2005). Does school-based dental screening for children increase follow-up treatment at dental school clinics? *J Dent Educ*, 69(3), 382-386.

Hegde, R., & Bull, G. Q. (2011). Performance of an agro-forestry based Payments-for-Environmental-Services project in Mozambique: A household level analysis. *Ecological Economics*, 71(Supplement C), 122-130.

Heinrich, C. J. (2007). Demand and supply-side determinants of conditional cash transfer program effectiveness. *World Development*, 35(1), 121-143.

Hidrobo, M., & Fernald, L. (2013). Cash transfers and domestic violence. *Journal of Health Economics*, 32(1), 304-319.

Hidrobo, M., Hoddinott, J., Peterman, A., Margolies, A., & Moreira, V. (2014). Cash, food, or vouchers? Evidence from a randomized experiment in northern Ecuador. *Journal of Development Economics*, 107, 144-156.

Hochstetter, A. S., Lombardo, M. J., D'Eramo, L., Piovano, S., & Bordoni, N. (2007). Effectiveness of a preventive educational programme on the oral health of preschool children. *Promot Educ*, 14(3), 155-158.

Hoddinott, J., Berhane, G., Gilligan, D. O., Kumar, N., & Taffesse, A. S. (2012). The Impact of Ethiopia's Productive Safety Net Programme and Related Transfers on Agricultural Productivity. *Journal of African Economies*, 21(5), 761-786.

Hoddinott, J, & Skoufias, E. (2004). The Impact of PROGRESA on Food Consumption. *Economic Development and Cultural Change*, 53(1), 37-61.

Hou, X. (2010). Can drought increase total calorie availability? The impact of drought on food consumption and the mitigating effects of a conditional cash transfer program. *Economic Development and Cultural Change*, 58(4), 713-737.

Hu, C., Ye, D., Li, Y., Huang, Y., Li, L., Gao, Y., & Wang, S. (2010). Evaluation of a kindergarten-based nutrition education intervention for pre-school children in China. *Public Health Nutr*, 13(2), 253-260.

Huang, H., Ye, X., Cai, Y., Shen, L., Xu, G., Shi, R., & Jin, X. (2008). Study on peer-led school-based HIV/AIDS prevention among youths in a medium-sized city in China. *Int J STD AIDS*, 19(5), 342-346.

Huda, S. N., Grantham-McGregor, S. M., & Tomkins, A. (2001). Cognitive and motor functions of iodine-deficient but euthyroid children in Bangladesh do not benefit from iodized poppy seed oil (Lipiodol). *J Nutr*, 131(1), 72-77.

Huerta, M. C. (2006). Child Health in Rural Mexico: Has Progresa Reduced Children's Morbidity Risks? *Social Policy & Administration*, 40(6), 652-677.

Hulett, J. L., Weiss, R. E., Bwibo, N. O., Galal, O. M., Drorbaugh, N., & Neumann, C. G. (2013). Animal source foods have a positive impact on the primary school test scores of Kenyan schoolchildren in a cluster-randomised, controlled feeding intervention trial. *British Journal of Nutrition*, 111(5), 875-886.

Huque, R., Dogar, O., Cameron, I., Thomson, H., Amos, A., & Siddiqi, K. (2015). Children Learning About Second-Hand Smoking: A Feasibility Cluster Randomized Controlled Trial. *Nicotine & Tobacco Research*, 17(12), 1465-1472.

Ibrahim, N., Rampal, L., Jamil, Z., & Zain, A. M. (2012). Effectiveness of peer-led education on knowledge, attitude and risk behavior practices related to HIV among students at a Malaysian public university--a randomized controlled trial. *Prev Med*, 55(5), 505-510.

Iritani, B. J., Cho, H., Rusakaniko, S., Mapfumo, J., Hartman, S., & Hallfors, D. D. (2016). Educational Outcomes for Orphan Girls in Rural Zimbabwe: Effects of a School Support Intervention. *Health Care for Women International*, 37(3), 303-324.

Jacoby, E., Cueto, S., & Pollitt, E. (1996). Benefits of a school breakfast programme among Andean children in Huaraz, Peru. *Food and Nutrition Bulletin*, 17(1), 54-64.

Jahan, K., Roy, S. K., Mihrshahi, S., Sultana, N., Khatoon, S., Roy, H., Datta, L. R., Roy, A., Jahan, S., Khatun, W., Nahar, N., & Steele, J. (2014). Short-Term Nutrition Education Reduces Low Birthweight and Improves Pregnancy Outcomes among Urban Poor Women in Bangladesh. *Food and Nutrition Bulletin*, 35(4), 414-421.

Jahanfar, S., Lye, M. S., & Rampal, L. (2009). A randomised controlled trial of peer-adult-led intervention on improvement of knowledge, attitudes and behaviour of university students regarding HIV/AIDS in Malaysia. *Singapore Med J*, 50(2), 173-180.

Jain, M. (2015). India's Struggle Against Malnutrition—Is the ICDS Program the Answer? *World Development*, 67(Supplement C), 72-89.

James, S., Reddy, P. S., Ruiter, R. A., Taylor, M., Jinabhai, C. C., Van Empelen, P., & Van den Borne, B. (2005). The effects of a systematically developed photo-novella on knowledge, attitudes, communication and behavioural intentions with respect to sexually transmitted infections among secondary school learners in South Africa. *Health Promot Int*, 20(2), 157-165.

James, S., Reddy, P., Ruiter, R. A., McCauley, A., & van den Borne, B. (2006). The Impact of an HIV and AIDS Life Skills Program on Secondary School Students in KwaZulu-Natal, South Africa. *AIDS Educ Prev*, 18(4), 281-294.

Jan, S., Ferrari, G., Watts, C. H., Hargreaves, J. R., Kim, J. C., Phetla, G., Morison, L. A., Porter, J. D., Barnett, T., & Pronyk, P. M. (2011). Economic evaluation of a combined microfinance and gender training intervention for the prevention of intimate partner violence in rural South Africa. *Health Policy Plan*, 26(5), 366-372.

Jayaraman, R., & Simroth, D. (2015). The Impact of School Lunches on Primary School Enrollment: Evidence from India's Midday Meal Scheme. *The Scandinavian Journal of Economics*, 117(4), 1176-1203.

Jemmott, J. B., 3rd, Jemmott, L. S., O'Leary, A., Ngwane, Z., Icard, L. D., Bellamy, S. L., Jones, S. F., Landis, J. R., Heeren, G. A., Tyler, J. C., & Makiwane, M. B. (2010). School-based randomized controlled trial of an HIV/STD risk-reduction intervention for South African adolescents. *Arch Pediatr Adolesc Med*, 164(10), 923-929.

Jemmott, J. B., 3rd, Jemmott, L. S., O'Leary, A., Ngwane, Z., Icard, L., Bellamy, S., Jones, S., Landis, J. R., Heeren, G. A., Tyler, J. C., & Makiwane, M. B. (2011). Cognitive-behavioural health-promotion intervention increases fruit and vegetable consumption and physical activity among South African adolescents: a cluster-randomised controlled trial. *Psychol Health*, 26(2), 167-185.

Jennings, L., Ssewamala, F. M., & Nabunya, P. (2016). Effect of savings-led economic empowerment on HIV preventive practices among orphaned adolescents in rural

Uganda: results from the Suubi-Maka randomized experiment. *AIDS Care*, 28(3), 273-282.

Jiang, J., Xia, X., Greiner, T., Wu, G., Lian, G., & Rosenqvist, U. (2007). The effects of a 3-year obesity intervention in schoolchildren in Beijing. *Child Care Health Dev*, 33(5), 641-646.

Jinabhai, C. C., Taylor, M., Coutsoudis, A., Coovadia, H. M., Tomkins, A. M., & Sullivan, K. R. (2001). A randomized controlled trial of the effect of antihelminthic treatment and micronutrient fortification on health status and school performance of rural primary school children. *Ann Trop Paediatr*, 21(4), 319-333.

Jinabhai, C. C., Taylor, M., Coutsoudis, A., Coovadia, H. M., Tomkins, A. M., & Sullivan, K. R. (2001). Epidemiology of helminth infections: implications for parasite control programmes, a South African perspective. *Public Health Nutr*, 4(6), 1211-1219.

Jindal, R., Kerr, J. M., & Carter, S. (2012). Reducing Poverty Through Carbon Forestry? Impacts of the N'hambita Community Carbon Project in Mozambique. *World Development*, 40(100), 2123-2135.

Jones, K. M., & de Brauw, A. (2015). Using Agriculture to Improve Child Health: Promoting Orange Sweet Potatoes Reduces Diarrhea. *World Development*, 74(Supplement C), 15-24.

Jordans, M. J., Komproe, I. H., Tol, W. A., Kohrt, B. A., Luitel, N. P., Macy, R. D., & de Jong, J. T. (2010). Evaluation of a classroom-based psychosocial intervention in conflict-affected Nepal: a cluster randomized controlled trial. *J Child Psychol Psychiatry*, 51(7), 818-826.

Juarez, L. (2009). Crowding out of private support to the elderly: Evidence from a demogrant in Mexico. *Journal of Public Economics*, 93(3), 454-463.

Jukes, M. C. H., Simmons Zuilkowski, S., Parawan, A., & Lee, S. (2014). Evaluating The Cognitive Impacts Of School Health In The Philippines. *International Journal of Child Health and Human Development*, 7(1), 55-66.

Jumbe, C. B. L., & Angelsen, A. (2006). Do the Poor Benefit from Devolution Policies? Evidence from Malawi's Forest Co-Management Program. *Land Economics*, 82(4), 562-581.

Jumoke, A. R. (2012). Impact of Fadama-II project on poverty reduction of rural households in Nigeria. *International Journal of Agricultural Science and Research*, 2(2), 18-38.

Kain, J., Leyton, B., Cerda, R., Vio, F., & Uauy, R. (2009). Two-year controlled effectiveness trial of a school-based intervention to prevent obesity in Chilean children. *Public Health Nutr*, 12(9), 1451-1461.

Kandpal, E. (2011). Beyond average treatment effects: distribution of child nutrition outcomes and program placement in India's ICDS. *World Development*, 39(8), 1410-1421.

Kapadia-Kundu, N., Storey, D., Safi, B., Trivedi, G., Tupe, R., & Narayana, G. (2014). Seeds of prevention: The impact on health behaviors of young adolescent girls in Uttar Pradesh, India, a cluster randomized control trial. *Social Science & Medicine*, 120(Supplement C), 169-179.

Karim, A. M., Williams, T., Patykewich, L., Ali, D., Colvin, C. E., Posner, J., & Rutaremwa, G. (2009). The impact of the African Youth Alliance program on the sexual behavior of young people in Uganda. *Stud Fam Plann*, 40(4), 289-306.

Karimli, L., Ssewamala, F. M., & Neilands, T. B. (2014). Poor Families Striving to Save in Matched Children's Savings Accounts: Findings from a Randomized Experimental Design in Uganda. *The Social Service Review*, 88(4), 658–694.

Karnell, A. P., Cupp, P. K., Zimmerman, R. S., Feist-Price, S., & Bennie, T. (2006). Efficacy of an American alcohol and HIV prevention curriculum adapted for use in South Africa: results of a pilot study in five township schools. *AIDS Educ Prev*, 18(4), 295-310.

Kasturiba, B., Naik, R. K., & Basarkar, P. (2007). Impact of Synthetic Vitamin A and Horticulture Intervention on Vitamin A Status and Iron Status of Rural School Children. *J. Hum. Ecol*, 22(3), 251-254.

Kazianga, H., de Walque, D., & Alderman, H. (2012). Educational and child labour impacts of two Food-for-Education Schemes: evidence from a randomised trial in Rural Burkina Faso. *Journal of African Economies*, 21(5), 723-760.

Kazianga, H., de Walque, D., & Alderman, H. (2014). School feeding programs, intrahousehold allocation and the nutrition of siblings: Evidence from a randomized trial in rural Burkina Faso. *Journal of Development Economics*, 106(Supplement C), 15-34.

Kazianga, H., Levy, D., Linden, L., & Sloan, M. (2013). The Effects of 'Girl-Friendly' Schools: Evidence from the BRIGHT School Construction Program in Burkina Faso. *American Economic Journal: Applied Economics*, 5(3), 41-62.

Kelly, P., Musonda, R., Kafwembe, E., Kaetano, L., Keane, E., & Farthing, M. (1999). Micronutrient supplementation in the AIDS diarrhoea-wasting syndrome in Zambia: a randomized controlled trial. *AIDS*, 13(4), 495-500.

Khoshnevisan, F., Kimiagar, M., Kalantaree, N., Valaee, N., & Shaheedee, N. (2004). Effect of nutrition education and diet modification in iron depleted preschool children in nurseries in Tehran: a pilot study. *Int J Vitam Nutr Res*, 74(4), 264-268.

Kikafunda, J. K., Walker, A. F., Allan, E. F., & Tumwine, J. K. (1998). Effect of zinc supplementation on growth and body composition of Ugandan preschool children: a randomized, controlled, intervention trial. *Am J Clin Nutr*, 68(6), 1261-1266.

Kim, D. A., Hwong, A. R., Stafford, D., Hughes, D. A., O'Malley, A. J., Fowler, J. H., & Christakis, N. A. (2015). Social network targeting to maximise population behaviour change: a cluster randomised controlled trial. *The Lancet*, 386(9989), 145-153.

Kim, J. C., Watts, C. H., Hargreaves, J. R., Ndhlovu, L. X., Phetla, G., Morison, L. A., Busza, J., Porter, J. D., & Pronyk, P. (2007). Understanding the impact of a microfinance-based intervention on women's empowerment and the reduction of intimate partner violence in South Africa. *Am J Public Health*, 97(10), 1794-1802.

Kim, J., Ferrari, G., Abramsky, T., Watts, C., Hargreaves, J., Morison, L., Phetla, G., Porter, J., & Pronyk, P. (2009). Assessing the incremental effects of combining economic and health interventions: the IMAGE study in South Africa. *Bull World Health Organ*, 87(11), 824-832.

Kinsman, J., Nakiyingi, J., Kamali, A., Carpenter, L., Quigley, M., Pool, R., & Whitworth, J. (2001). Evaluation of a comprehensive school-based AIDS education programme in rural Masaka, Uganda. *Health Educ Res*, 16(1), 85-100.

Kinung'hi, S. M., Magnussen, P., Kishamawe, C., Todd, J., & Vennervald, B. J. (2015). The impact of anthelmintic treatment intervention on malaria infection and anaemia in school and preschool children in Magu district, Tanzania: an open label randomised intervention trial. *BMC Infectious Diseases*, 15(1), 136.

Kleiman-Weiner, M., Luo, R., Zhang, L., Shi, Y., Medina, A., & Rozelle, S. (2013). Eggs versus chewable vitamins: Which intervention can increase nutrition and test scores in rural China? *China Economic Review*, 24, 165-176.

Klepp, K. I., Ndeki, S. S., Seha, A. M., Hannan, P., Lyimo, B. A., Msuya, M. H., Irema, M. N., & Schreiner, A. (1994). AIDS education for primary school children in Tanzania: an evaluation study. *AIDS*, 8(8), 1157-1162.

Kohler, H. P., & Thornton, R. (2012). Conditional Cash Transfers and HIV/AIDS Prevention: Unconditionally Promising? *World Bank Econ Rev*, 26(2), 165-190.

Kotb, M., Al-Tehawy, M., El-Setouhy, M., & Hussein, H. (1998). Evaluation of a school-based health education model in schistosomiasis: a randomized community trial. *Eastern Mediterranean Health Journal*, 4(2), 265-275.

Kremer, M., & Miguel, E. (2007). The illusion of sustainability. *The Quarterly Journal of Economics*, 122(3), 1007-1065.

Kumakech, E., Cantor-Graae, E., Maling, S., & Bajunirwe, F. (2009). Peer-group support intervention improves the psychosocial well-being of AIDS orphans: cluster randomized trial. *Soc Sci Med*, 68(6), 1038-1043.

Labonne, J. (2013). The local electoral impacts of conditional cash transfers: Evidence from a field experiment. *Journal of Development Economics*, 104(Supplement C), 73-88.

Lalloo, R., & Solanki, G. S. (1994). An evaluation of a school-based comprehensive public oral health care programme. *Community Dent Health*, 11(3), 152-155.

Lamadrid-Figueroa, H., Ángeles, G., Mroz, T., Urquieta-Salomon, J., Hernandez-Prado, B., Cruz-Valdez, A., & Téllez-Rojo, M. M. (2010). Heterogeneous impact of the social programme Oportunidades on use of contraceptive methods by young adult women living in rural areas. *Journal of Development Effectiveness*, 2(1), 74-86.

Landmann, A., & Frölich, M. (2015). Can health-insurance help prevent child labor? An impact evaluation from Pakistan. *Journal of Health Economics*, 39, 51-59.

Larke, N., Cleophas-Mazige, B., Plummer, M. L., Obasi, A. I., Rwakatare, M., Todd, J., Changalucha, J., Weiss, H. A., Hayes, R. J., & Ross, D. A. (2010). Impact of the MEMA kwa Vijana adolescent sexual and reproductive health interventions on use of health services by young people in rural Mwanza, Tanzania: results of a cluster randomized trial. *J Adolesc Health*, 47(5), 512-522.

Larocque, R., Casapia, M., Gotuzzo, E., MacLean, J. D., Soto, J. C., Rahme, E., & Gyorkos, T. W. (2006). A double-blind randomized controlled trial of antenatal mebendazole to reduce low birthweight in a hookworm-endemic area of Peru. *Trop Med Int Health*, 11(10), 1485-1495.

Larranaga, O., Contreras, D., & Ruiz-Tagle, J. (2012). Impact evaluation of Chile Solidario: lessons and policy recommendations. *Journal of Latin American Studies*, 44(2), 347-372.

Layne, C. M., Saltzman, W. R., Poppleton, L., Burlingame, G. M., Pasalic, A., Durakovic, E., Music, M., Campara, N., Dapo, N., Arslanagic, B., Steinberg, A. M., & Pynoos, R. S. (2008). Effectiveness of a school-based group psychotherapy program for war-exposed adolescents: a randomized controlled trial. *J Am Acad Child Adolesc Psychiatry*, 47(9), 1048-1062.

Le Huong, T., Brouwer, I. D., Nguyen, K. C., Burema, J., & Kok, F. J. (2007). The effect of iron fortification and de-worming on anaemia and iron status of Vietnamese schoolchildren. *Br J Nutr*, 97(5), 955-962.

Leroy, J. L., Gadsden, P., González de Cossío, T., & Gertler, P. (2013). Cash and in-Kind Transfers Lead to Excess Weight Gain in a Population of Women with a High Prevalence of Overweight in Rural Mexico. *The Journal of Nutrition*, 143(3), 378-383.

Leroy, J. L., Gadsden, P., Rodríguez-Ramírez, S., & de Cossío, T. G. (2010). Cash and In-Kind Transfers in Poor Rural Communities in Mexico Increase Household Fruit, Vegetable, and Micronutrient Consumption but Also Lead to Excess Energy Consumption. *The Journal of Nutrition*, 140(3), 612-617.

Leroy, J. L., Garcia-Guerra, A., Garcia, R., Dominguez, C., Rivera, J., & Neufeld, L. M. (2008). The *Oportunidades* program increases the linear growth of children enrolled at young ages in urban Mexico. *J Nutr*, 138(4), 793-798.

Leung, C., Tsang, S., & Heung, K. (2015). The Effectiveness of Healthy Start Home Visit Program. *Research on Social Work Practice*, 25(3), 322-333.

Levely, I. (2014). Measuring Intermediate Outcomes of Liberia's Disarmament, Demobilization, Rehabilitation and Reintegration Program. *Defence and Peace Economics*, 25(2), 139-162.

Leventhal, K. S., DeMaria, L. M., Gillham, J. E., Andrew, G., Peabody, J., & Leventhal, S. M. (2016). A psychosocial resilience curriculum provides the “missing piece” to boost adolescent physical health: A randomized controlled trial of Girls First in India. Social Science & Medicine, 161(Supplement C), 37-46.

Leventhal, K. S., Gillham, J., DeMaria, L., Andrew, G., Peabody, J., & Leventhal, S. (2015). Building psychosocial assets and wellbeing among adolescent girls: A randomized controlled trial. Journal of Adolescence, 45(Supplement C), 284-295.

Levy, D., & Ohls, J. (2010). Evaluation of Jamaica's PATH conditional cash transfer programme. *Journal of Development Effectiveness*, 2(4), 421-441.

Li, S., Huang, H., Cai, Y., Ye, X., Shen, X., Shi, R., & Xu, G. (2010). Evaluation of a school-based HIV/AIDS peer-led prevention programme: the first intervention trial for children of migrant workers in China. *Int J STD AIDS*, 21(2), 82-86.

Li, X., Stanton, B., Wang, B., Mao, R., Zhang, H., Qu, M., Sun, Z., & Wang, J. (2008). Cultural adaptation of the Focus on Kids program for college students in China. *AIDS Educ Prev*, 20(1), 1-14.

Li, X., Zhang, L., Mao, R., Zhao, Q., & Stanton, B. (2011). Effect of social cognitive theory-based HIV education prevention program among high school students in Nanjing, China. *Health Educ Res*, 26(3), 419-431.

Li, X. H., Lin, S., Guo, H., Huang, Y., Wu, L., Zhang, Z., Ma, J., & Wang, H. J. (2014). Effectiveness of a school-based physical activity intervention on obesity in school children: a nonrandomized controlled trial. *BMC Public Health*, 14(1), 1282.

Li, Y. P., Hu, X. Q., Schouten, E. G., Liu, A. L., Du, S. M., Li, L. Z., Cui, Z. H., Wang, D., Kok, F. J., Hu, F. B., & Ma, G. S. (2010). Report on childhood obesity in China (8): effects and sustainability of physical activity intervention on body composition of Chinese youth. *Biomed Environ Sci*, 23(3), 180-187.

Licos Jr, A. R. (2011). Child-to-child school health program (CtCSHP): Its impact on the oral health behavior of grade 1 pupils in the division of La Union, Philippines. *保健医療科学*, 60(1), 44-49.

Linkie, M., Smith, R. J., Zhu, Y., Martyr, D. J., Suedmeyer, B., Pramono, J., & Leader-Williams, N. (2008). Evaluating Biodiversity Conservation around a Large Sumatran Protected Area. *Conservation Biology*, 22, 683–690.

Liu, C., Mullan, K., Liu, H., Zhu, W., & Rong, Q. (2014). The estimation of long term impacts of China's key priority forestry programs on rural household incomes. *Journal of Forest Economics*, 20(3), 267-285.

Liu, N., Mao, L., Sun, X., Liu, L., Yao, P., & Chen, B. (2009). The effect of health and nutrition education intervention on women's postpartum beliefs and practices: a randomized controlled trial. *BMC Public Health*, 9, 45.

Liu, T., Liu, C., Liu, H., Wang, S., Rong, Q., & Zhu, W. (2014). Did the Key Priority Forestry Programs affect income inequality in rural China? *Land Use Policy*, 38(Supplement C), 264-275.

Lobato, L., Miranda, A., Faria, I. M., Bethony, J. M., & Gazzinelli, M. F. (2012). Development of cognitive abilities of children infected with helminths through health education. *Rev Soc Bras Med Trop*, 45(4), 514-519.

Lotrean, L. M., Dijk, F., Mesters, I., Ionut, C., & De Vries, H. (2010). Evaluation of a peer-led smoking prevention programme for Romanian adolescents. *Health Educ Res*, 25(5), 803-814.

Low, J. W., Arimond, M., Osman, N., Cunguara, B., Zano, F., & Tschirley, D. (2007). A food-based approach introducing orange-fleshed sweet potatoes increased vitamin A intake and serum retinol concentrations in young children in rural Mozambique. *J Nutr*, 137(5), 1320-1327.

Lule, J. R., Mermin, J., Ekwaru, J. P., Malamba, S., Downing, R., Ransom, R., Nakanjako, D., Wafula, W., Hughes, P., Bunnell, R., Kaharuza, F., Coutinho, A., Kigozi, A., & Quick, R. (2005). Effect of home-based water chlorination and safe storage on diarrhea among persons with human immunodeficiency virus in Uganda. *Am J Trop Med Hyg*, 73(5), 926-933.

Luo, R., Shi, Y., Zhang, L., Liu, C., Rozelle, S., Sharbono, B., Yue, A., Zhao, Q., & Martorell, R. (2012). Nutrition and educational performance in rural China’s elementary schools: Results of a randomized control trial in Shaanxi Province. *Economic Development and Cultural Change*, 60(4), 735-772.

Lutge, E., Lewin, S., Volmink, J., Friedman, I., & Lombard, C. (2013). Economic support to improve tuberculosis treatment outcomes in South Africa: a pragmatic cluster-randomized controlled trial. *Trials*, 14(1), 154.

Lwegaba, A. (2005). Field trial to test and evaluate primary tobacco prevention methods in clusters of elementary schools in Barbados. *West Indian Med J*, 54(5), 283-291.

Ma, X., Zhou, Z., Yi, H., Pang, X., Shi, Y., Chen, Q., Meltzer, M. E., le Cessie, S., He, M., Rozelle, S., Liu, Y., & Congdon, N. (2014). Effect of providing free glasses on children’s educational outcomes in China: cluster randomized controlled trial. *BMJ : British Medical Journal*, 349.

Macours, K., Schady, N., & Vakis, R. (2012). Cash transfers, behavioral changes, and cognitive development in early childhood: Evidence from a randomized experiment. *American Economic Journal: Applied Economics*, 4(2), 247-273.

Magnani, R. J., Gaffikin, L., de Aquino, E. M., Seiber, E. E., Almeida, M. C., & Lipovsek, V. (2001). Impact of an integrated adolescent reproductive health program in Brazil. *Stud Fam Plann*, 32(3), 230-243.

Magnani, R., Macintyre, K., Karim, A. M., Brown, L., Hutchinson, P., Kaufman, C., Rutenburg, N., Hallman, K., May, J., Dallimore, A., & Transitions Study, T. (2005). The impact of life skills education on adolescent sexual risk behaviors in KwaZulu-Natal, South Africa. *J Adolesc Health*, 36(4), 289-304.

Mahawithanage, S. T., Kannangara, K. K., Wickremasinghe, R., Chandrika, U.G., Jansz, E.R., Karunaweera N. D., & Wickremasinghe, A.R. (2007). Impact Of Vitamin A Supplementation On Health Status And Absenteeism Of School Children In Sri Lanka. *Asia Pacific Journal of Clinical Nutrition*, 16(1), pp. 94-102.

Malcon, M. C., Menezes, A. M. B., Assunção, M. C. F., Neutzling, M. B., & Challal, P. (2011). Effectiveness of an educational intervention on smoking among school adolescents. *Revista Brasileira de Epidemiologia*, 14(1), 63-72.

Mallick, D. (2013). How Effective is a Big Push to the Small? Evidence from a Quasi-Experiment. *World Development*, 41(Supplement C), 168-182.

Maluccio, J. A. (2010). The impact of conditional cash transfers on consumption and investment in Nicaragua. *The Journal of Development Studies*, 46(1), 14-38.

Maluccio, J. A., Murphy, A., & Regalia, F. (2010). Does supply matter? Initial schooling conditions and the effectiveness of conditional cash transfers for grade progression in Nicaragua. *Journal of Development Effectiveness*, 2(1), 87-116.

Maluccio, J. A., Palermo, T., Kadiyala, S., & Rawat, R. (2015). Improving Health-Related Quality of Life among People Living with HIV: Results from an Impact Evaluation of a Food Assistance Program in Uganda. *PLOS ONE*, 10(8), e0135879.

Manacorda, M., Miguel, E., & Vigorito, A. (2011). Government transfers and political support. *American Economic Journal: Applied Economics*, 3, 1-28.

Manger, M. S., McKenzie, J. E., Winichagoon, P., Gray, A., Chavasit, V., Pongcharoen, T., Gowachirapant, S., Ryan, B., Wasantwisut, E., & Gibson, R. S. (2008). A micronutrient-fortified seasoning powder reduces morbidity and improves short-term cognitive function, but has no effect on anthropometric measures in primary school children in northeast Thailand: a randomized controlled trial. *Am J Clin Nutr*, 87(6), 1715-1722.

Mario, A., Rosa, P., & Ariel, G. (2013). Politicas Sociales Y Mercado De Trabajo En Argentina: El Efecto de la AUH En La Informalidad Laboral. *Documentos y Aportes en Administración Pública y Gestión Estatal*, 13(21), 41-64.

Martinez, O. A. (2012). Effects on School Attendance of the Oportunidades Program Scholarships. The Case of the Urban Zones at Northeast Mexico. *Revista Desarrollo y Sociedad*, 69, 99-131.

Martinez-Donate, A. P., Hovell, M. F., Zellner, J., Sipan, C. L., Blumberg, E. J., & Carrizosa, C. (2004). Evaluation of two school-based HIV prevention interventions in the border city of Tijuana, Mexico. *J Sex Res*, 41(3), 267-278.

Martiniuk, A. L., O'Connor, K. S., & King, W. D. (2003). A cluster randomized trial of a sex education programme in Belize, Central America. *Int J Epidemiol*, 32(1), 131-136.

Martins, N., Morris, P., & Kelly, P. M. (2009). Food incentives to improve completion of tuberculosis treatment: randomised controlled trial in Dili, Timor-Leste. *BMJ*, 339, b4248.

Martorano, B., & Sanfilippo, M. (2012). Innovative Features In Poverty Reduction Programmes: An Impact Evaluation Of Chile Solidario On Households And Children. *Journal of International Development*, 24(8), 1030-1041.

Mathews, C., Aaro, L. E., Grimsrud, A., Flisher, A. J., Kaaya, S., Onya, H., Schaalma, H., Wubs, A., Mukoma, W., & Klepp, K. I. (2012). Effects of the SATZ teacher-led school HIV prevention programmes on adolescent sexual behaviour: cluster randomised controlled trials in three sub-Saharan African sites. *Int Health*, 4(2), 111-122.

Maticka-Tyndale, E., Wildish, J., & Gichuru, M. (2007). Quasi-experimental evaluation of a national primary school HIV intervention in Kenya. *Evaluation and Program Planning*, 30(2), 172-186.

Maticka-Tyndale, E., Wildish, J., & Gichuru, M. (2010). Thirty-month quasi-experimental evaluation follow-up of a national primary school HIV intervention in Kenya. *Sex Education*, 10(2), 113-130.

Mattos, E., Maia, S., & Marques, F. (2010). Evidencias da relacao entre oferta de trabalho e programas de transferencia de renda no Brasil: Bolsa Escola versus Renda Minima. *Pesquisa e Planejamento Econômico*, 40(2).

Maurizio, R., & Vázquez, G. (2014). Argentina: impacts of the child allowance programme on the labour-market behaviour of adults. *Cepal Review*, 113.

Mauro, V., Biggeri, M., & Grilli, L. (2015). Does Community-Based Rehabilitation Enhance the Multidimensional Well-Being of Deprived Persons With Disabilities? A Multilevel Impact Evaluation. *World Development*, 76(Supplement C), 190-202.

Mauro, V., Biggeri, M., Deepak, S., & Trani, J. F. (2014). The effectiveness of community-based rehabilitation programmes: an impact evaluation of a quasi-randomised trial. *Journal of Epidemiology and Community Health*, 68(11), 1102-1108.

Mayer, M. P., de Paiva Buischi, Y., de Oliveira, L. B., & Gjermo, O. (2003). Long-term effect of an oral hygiene training program on knowledge and reported behavior. *Oral Health Prev Dent*, 1(1), 37-43.

Mbawalla, H., Masalu, J. R., Masatu, M., & Åstrøm, A. N. (2013). Changes in adolescents' oral health status following oral health promotion activities in Tanzania. *Acta Odontologica Scandinavica*, 71(2), 333-342.

Mbizvo, M. T., Kasule, J., Gupta, V., Rusakaniko, S., Kinoti, S. N., Mpanju-Shumbushu, W., Sebina-Zziwa, A. J., Mwateba, R., & Padayachy, J. (1997). Effects of a randomized health education intervention on aspects of reproductive health knowledge and reported behaviour among adolescents in Zimbabwe. *Soc Sci Med*, 44(5), 573-577.

McEwan, P. J. (2013). The impact of Chile's school feeding program on education outcomes. *Economics of Education Review*, 32, 122-139.

Mehta, S., Mugusi, F. M., Bosch, R. J., Aboud, S., Chatterjee, A., Finkelstein, J. L., Fataki, M., Kisenge, R., & Fawzi, W. W. (2011). A randomized trial of multivitamin supplementation in children with tuberculosis in Tanzania. *Nutrition Journal*, 10, 120.

Melo, R. D. S., & Duarte, G. B. (2010). Impacto do Programa Bolsa Família sobre a frequência escolar: o caso da agricultura familiar no Nordeste do Brasil. *Revista de Economia e Sociologia Rural*, 48(3), 635-657.

Meng, X., & Ryan, J. (2010). Does a food for education program affect school outcomes? The Bangladesh case. *Journal of Population Economics*, 23(2), 415-447.

Menon, P., Ruel, M. T., Loechl, C. U., Arimond, M., Habicht, J. P., Pelto, G., & Michaud, L. (2007). Micronutrient Sprinkles reduce anemia among 9- to 24-mo-old children when delivered through an integrated health and nutrition program in rural Haiti. *J Nutr*, 137(4), 1023-1030.

Michielsen, K., Beauclair, R., Delva, W., Roelens, K., Van Rossem, R., & Temmerman, M. (2012). Effectiveness of a peer-led HIV prevention intervention in secondary schools in Rwanda: results from a non-randomized controlled trial. *BMC Public Health*, 12, 729.

Miguel, E., & Kremer, M. (2004). Worms: identifying impacts on education and health in the presence of treatment externalities. *Econometrica*, 72(1), 159-217.

Miller, G., Luo, R., Zhang, L., Sylvia, S., Shi, Y., Foo, P., Zhao, Q., Martorell, R., Medina, A., & Rozelle, S. (2012). Effectiveness of provider incentives for anaemia reduction in rural China: a cluster randomised trial. *BMJ*, 345, e4809.

Mo, D., Zhang, L., Yi, H., Luo, R., Rozelle, S., & Brinton, C. (2013). School Dropouts and Conditional Cash Transfers: Evidence from a Randomised Controlled Trial in Rural China's Junior High Schools. *The Journal of Development Studies*, 49(2), 190-207.

Morgan-Brown, T., Jacobson, S. K., Wald, K., & Child, B. (2010). Quantitative assessment of a Tanzanian integrated conservation and development project involving butterfly farming. *Conserv Biol*, 24(2), 563-572.

Morris, S. S., Flores, R., Olinto, P., & Medina, J. M. (2004). Monetary incentives in primary health care and effects on use and coverage of preventive health care interventions in rural Honduras: cluster randomised trial. *The Lancet*, 364(9450), 2030-2037.

Morris, S. S., Olinto, P., Flores, R., Nilson, E. A. F., & Figueiró, A. C. (2004). Conditional Cash Transfers Are Associated with a Small Reduction in the Rate of Weight Gain of Preschool Children in Northeast Brazil. *The Journal of Nutrition*, 134(9), 2336-2341.

Mo-suwan, L., Pongprapai, S., Junjana, C., & Puetpaiboon, A. (1998). Effects of a controlled trial of a school-based exercise program on the obesity indexes of preschool children. *Am J Clin Nutr*, 68(5), 1006-1011.

Mullally, C., & Maffioli, A. (2016). Extension and Matching Grants for Improved Management: An Evaluation of the Uruguayan Livestock Program. *American Journal of Agricultural Economics*, 98(1), 333-350.

Mutto, M., Kahn, K., Lett, R., & Lawoko, S. (2009). Piloting an educational response to violence in Uganda: prospects for a new curriculum: short research report. *African Safety Promotion: A Journal of Injury and Violence Prevention*, 7(2), 37-46.

Mwaniki, D., Omondi, B., Muniu, E., Thiong'o, F., Ouma, J., Magnussen, P., Geissler, P. W., Michaelsen, K. F., & Friis, H. (2002). Effects on serum retinol of multi-micronutrient supplementation and multi-helminth chemotherapy: a randomised, controlled trial in Kenyan school children. *European Journal of Clinical Nutrition*, 56(7), 666-673.

Mwidunda, S. A., Carabin, H., Matuja, W. B. M., Winkler, A. S., & Ngowi, H. A. (2015). A School Based Cluster Randomised Health Education Intervention Trial for Improving Knowledge and Attitudes Related to Taenia solium Cysticercosis and Taeniasis in Mbulu District, Northern Tanzania. *PLOS ONE*, 10(2), e0118541.

Nabipour, I., Imami, S. R., Mohammadi, M. M., Heidari, G., Bahramian, F., Azizi, F., Khosravizadegan, Z., Pazoki, R., Soltanian, A. R., Ramazanzadeh, M., Emadi, A., Arab, J., & Larijani, B. (2004). A school-based intervention to teach 3-4 grades children about healthy heart; the Persian Gulf healthy heart project. *Indian J Med Sci*, 58(7), 289-296.

Nabunya, P., Ssewamala, F. M., & Ilic, V. (2014). Family economic strengthening and parenting stress among caregivers of AIDS-orphaned children: Results from a cluster randomized clinical trial in Uganda. *Children and Youth Services Review*, 44(Supplement C), 417-421.

Nabunya, P., Ssewamala, F. M., Mukasa, M. N., Byansi, W., & Nattabi, J. (2015). Peer mentorship program on HIV/AIDS knowledge, beliefs, and prevention attitudes among

orphaned adolescents: an evidence based practice. *Vulnerable Children and Youth Studies*, 10(4), 345-356.

Nahar, B., Hossain, M. I., Hamadani, J. D., Ahmed, T., Huda, S. N., Grantham-McGregor, S. M., & Persson, L. A. (2012). Effects of a community-based approach of food and psychosocial stimulation on growth and development of severely malnourished children in Bangladesh: a randomised trial. *Eur J Clin Nutr*, 66(6), 701-709.

Naidoo, R., & Johnson, K. (2013). Community-based conservation reduces sexual risk factors for HIV among men. *Globalization and Health*, 9(1), 27.

Najimi, A., & Ghaffari, M. (2013). Promoting fruit and vegetable consumption among students: A randomized controlled trial based on social cognitive theory. *Journal of the Pakistan Medical Association*, 63 (10), 1235-1240.

Namisi, F., Aarø, L. E., Kaaya, S., Kajula, L. J., Kilonzo, G. P., Onya, H., Wubs, A., & Mathews, C. (2015). Adolescents' Communication with Parents, Other Adult Family Members and Teachers on Sexuality: Effects of School-Based Interventions in South Africa and Tanzania. *AIDS and Behavior*, 19(12), 2162-2176.

Nandha, B., & Krishnamoorthy, K. (2012). Impact of education campaign on community-based vector control in hastening the process of elimination of lymphatic filariasis in Tamil Nadu, South India. *Health Educ Res*, 27(4), 585-594.

Nascimento, M. O., & de Micheli, D. (2015). Evaluation of different school-based preventive interventions for reducing the use of psychotropic substances among students: a randomized study. *Ciencia & Saude Coletiva*, 20(8), 2499-2510.

Ndebele, M., Kasese-Hara, M., & Greyling, M. (2012). Application of the information, motivation and behavioural skills model for targeting HIV risk behaviour amongst adolescent learners in South Africa. *SAHARA-J*, 9 Suppl 1, S37-47.

Nega, F., Mathijs, E., Deckers, J., Haile, M., Nyssen, J., & Tollens, E. (2010). Rural Poverty Dynamics and Impact of Intervention Programs upon Chronic and Transitory Poverty in Northern Ethiopia*. *African development review*, 22(1), 92-114.

Neumann, C., Bwibo, N., Jiang, L., & Weiss, R. (2013). School snacks decrease morbidity in Kenyan schoolchildren: A cluster randomized, controlled feeding intervention trial. *Public Health Nutrition*, 16(9), 1593-1604.

Ng'ondi, N. B. (2012). Socio-demographic and service provision characteristics associated with primary school attendance among the Most Vulnerable Children in Tanzania. *Children and Youth Services Review*, 34(12), 2255-2262.

Nga, T. T., Winichagoon, P., Dijkhuizen, M. A., Khan, N. C., Wasantwisut, E., & Wieringa, F. T. (2011). Decreased parasite load and improved cognitive outcomes caused by deworming and consumption of multi-micronutrient fortified biscuits in rural Vietnamese schoolchildren. *Am J Trop Med Hyg*, 85(2), 333-340.

Nga, T. T., Winichagoon, P., Dijkhuizen, M. A., Khan, N. C., Wasantwisut, E., Furr, H., & Wieringa, F. T. (2009). Multi-micronutrient-fortified biscuits decreased prevalence of anemia and improved micronutrient status and effectiveness of deworming in rural Vietnamese school children. *J Nutr*, 139(5), 1013-1021.

Ngowi, H., Mlangwa, J. E., Mlozi, M. R., Tolma, E., Kassuku, A., Carabin, H., & Willingham III, A. L. (2009). Implementation and evaluation of a health-promotion strategy for control of *Taenia solium* infections in northern Tanzania. *International Journal of Health Promotion and Education*, 47(1), 24-34.

Nguyen, H. T., Hatt, L., Islam, M., Sloan, N. L., Chowdhury, J., Schmidt, J. O., Hossain, A., & Wang, H. (2012). Encouraging maternal health service utilization: an evaluation of the Bangladesh voucher program. *Soc Sci Med*, 74(7), 989-996.

Nkengfack, G. N., Torimiro, J. N., Ngogang, J., Binting, S., Roll, S., Tinnemann, P., & Englert, H. (2014). Effects of an HIV-Care-Program on immunological parameters in HIV-positive patients in Yaoundé, Cameroon: a cluster-randomized trial. *International Journal of Public Health*, 59(3), 509-517.

Nyamathi, A., Hanson, A. Y., Salem, B. E., Sinha, S., Ganguly, K. K., Leake, B., Yadav, K., & Marfisee, M. (2012). Impact of a rural village women (Asha) intervention on adherence to antiretroviral therapy in southern India. *Nurs Res*, 61(5), 353-362.

O'Leary, A., Jemmott, J. B., Jemmott, L. S., Bellamy, S., Ngwane, Z., Icard, L., & Gueits, L. (2012). Moderation and mediation of an effective HIV risk-reduction intervention for South African adolescents. *Ann Behav Med*, 44(2), 181-191.

Oduor, J., Kamau, A., & Mathenge, E. (2009). Evaluating the impact of microfranchising the distribution of anti-malarial drugs in Kenya on malaria mortality and morbidity. *Journal of Development Effectiveness*, 1(3), 353-377.

Ogunlade, A. O., Kruger, H. S., Jerling, J. C., Smuts, C. M., Covic, N., Hanekom, S. M., Mamabolo, R. L., & Kvalsvig, J. (2011). Point-of-use micronutrient fortification: lessons learned in implementing a preschool-based pilot trial in South Africa. *Int J Food Sci Nutr*, 62(1), 1-16.

Okanlawon, F. A., & Asuzu, M. C. (2012). Secondary School Adolescents' Perception of Risk in Sexual Behaviour in Rural Community Of Oyo State, Nigeria. *Journal of Community Medicine and Primary Health Care*, 24(1).

Okello, F. O., Stuer, F., Kidane, A., & Wube, M. (2013). Saving the sick and improving the socio-economic conditions of people living with HIV in Ethiopia through traditional burial groups. *Health Policy and Planning*, 28(5), 549-557.

Okonofua, F. E., Coplan, P., Collins, S., Oronsaye, F., Ogunsakin, D., Ogonor, J. T., Kaufman, J. A., & Heggenhougen, K. (2003). Impact of an intervention to improve treatment-seeking behavior and prevent sexually transmitted diseases among Nigerian youths. *Int J Infect Dis*, 7(1), 61-73.

Olney, D. K., Pedehombga, A., Ruel, M. T., & Dillon, A. (2015). A 2-Year Integrated Agriculture and Nutrition and Health Behavior Change Communication Program Targeted to Women in Burkina Faso Reduces Anemia, Wasting, and Diarrhea in Children 3–12.9 Months of Age at Baseline: A Cluster-Randomized Controlled Trial. *The Journal of Nutrition*, 145(6), 1317-1324.

Olsen, A., Thiong'o, F. W., Ouma, J. H., Mwaniki, D., Magnussen, P., Fleischer Michaelsen, K., Friis, H., & Wenzel Geissler, P. (2003). Effects of multimicronutrient supplementation on helminth reinfection: a randomized, controlled trial in Kenyan schoolchildren. *Transactions of The Royal Society of Tropical Medicine and Hygiene*, 97(1), 109-114.

Olsen, M. F., Abdissa, A., Kæstel, P., Tesfaye, M., Yilma, D., Girma, T., Wells, J. C. K., Ritz, C., Mølgaard, C., Michaelsen, K. F., Zerfu, D., Brage, S., Andersen, Å. B., & Friis, H. (2014). Effects of nutritional supplementation for HIV patients starting antiretroviral treatment: randomised controlled trial in Ethiopia. *BMJ: British Medical Journal*, 348.

Omwami, E. M., Neumann, C., & Bwibo, N. O. (2011). Effects of a school feeding intervention on school attendance rates among elementary schoolchildren in rural Kenya. *Nutrition*, 27(2), 188-193.

Osei-Fosu, A. K. (2011). Evaluating The Impact Of The Capitation Grant And The School Feeding Programme On Enrollment, Attendance And Retention In Schools: The Case Of Weweso Circuit. *Journal of Science and Technology*, 31(1), 55.

Osendarp, S. J., Baghurst, K. I., Bryan, J., Calvaresi, E., Hughes, D., Hussaini, M., Karyadi, S. J., van Klinken, B. J., van der Knaap, H. C., Lukito, W., Mikarsa, W., Transler, C., Wilson, C., & Group, N. S. (2007). Effect of a 12-mo micronutrient intervention on learning and memory in well-nourished and marginally nourished school-aged children: 2 parallel, randomized, placebo-controlled studies in Australia and Indonesia. *Am J Clin Nutr*, 86(4), 1082-1093.

Ozer, E. J., Fernald, L. C., Manley, J. G., & Gertler, P. J. (2009). Effects of a conditional cash transfer program on children's behavior problems. *Pediatrics*, 123(4), e630-637.

Ozer, E. J., Fernald, L. C., Weber, A., Flynn, E. P., & VanderWeele, T. J. (2011). Does alleviating poverty affect mothers' depressive symptoms? A quasi-experimental investigation of Mexico's Oportunidades programme. *International Journal of Epidemiology*, 40(6), 1565-1576.

Pandejpong, D., Danchaivijitr, S., Vanprapa, N., Pandejpong, T., & Cook, E. F. (2012). Appropriate time-interval application of alcohol hand gel on reducing influenza-like illness among preschool children: a randomized, controlled trial. *Am J Infect Control*, 40(6), 507-511.

Patel, M. K., Harris, J. R., Juliao, P., Nygren, B., Were, V., Kola, S., Sadumah, I., Faith, S. H., Otieno, R., Obure, A., Hoekstra, R. M., & Quick, R. (2012). Impact of a hygiene

curriculum and the installation of simple handwashing and drinking water stations in rural Kenyan primary schools on student health and hygiene practices. *Am J Trop Med Hyg*, 87(4), 594-601.

Patil, S. R., Arnold, B. F., Salvatore, A. L., Briceno, B., Ganguly, S., Colford, J. M., Jr., & Gertler, P. J. (2014). The Effect of India's Total Sanitation Campaign on Defecation Behaviors and Child Health in Rural Madhya Pradesh: A Cluster Randomized Controlled Trial. *PLOS Medicine*, 11(8), e1001709.

Peletz, R., Simunyama, M., Sarenje, K., Baisley, K., Filteau, S., Kelly, P., & Clasen, T. (2012). Assessing water filtration and safe storage in households with young children of HIV-positive mothers: a randomized, controlled trial in Zambia. *PLoS One*, 7(10), e46548.

Peltonen, K., Qouta, S., El Sarraj, E., & Punamäki, R. L. (2012). Effectiveness of school-based intervention in enhancing mental health and social functioning among war-affected children. *Traumatology*, 18(4), 37.

Penny, M. E., Creed-Kanashiro, H. M., Robert, R. C., Narro, M. R., Caulfield, L. E., & Black, R. E. (2005). Effectiveness of an educational intervention delivered through the health services to improve nutrition in young children: a cluster-randomised controlled trial. *The Lancet*, 365(9474), 1863-1872.

Pérez, W., Blandón, E. Z., Persson, L. Å., Peña, R., & Källestål, C. (2012). Progress towards millennium development goal 1 in northern rural Nicaragua: Findings from a health and demographic surveillance site. *International Journal for Equity in Health*, 11(1), 43.

Perova, E., & Vakis, R. (2012). 5 Years in Juntos: New Evidence on the Program's Short and Long-Term Impacts. *Revista Economía*, 35(69).

Perry, C. L., Stigler, M. H., Arora, M., & Reddy, K. S. (2009). Preventing tobacco use among young people in India: Project MYTRI. *American journal of Public Health*, 99(5), 899.

Pick, S., Givaudan, M., Sirkin, J., & Ortega, I. (2007). Communication as a protective factor: evaluation of a life skills HIV/AIDS prevention program for Mexican elementary-school students. *AIDS Education & Prevention*, 19(5), 408-421.

Pinkaew, S., Winichagoon, P., Hurrell, R. F., & Wegmuller, R. (2013). Extruded rice grains fortified with zinc, iron, and vitamin A increase zinc status of Thai school children when incorporated into a school lunch program. *J Nutr*, 143(3), 362-368.

Pollitt, E., Jacoby, E., & Cueto, S. (1996). School breakfast and cognition among nutritionally at-risk children in the Peruvian Andes. *Nutrition Reviews*, 54(4) 22-26.

Ponce, J., & Bedi, A. S. (2010). The impact of a cash transfer program on cognitive achievement: The Bono de Desarrollo Humano of Ecuador. *Economics of Education Review*, 29(1), 116-125.

Posse, M., Tirivayi, N., Saha, U. R., & Baltussen, R (2013). The effect of Food Assistance on Adherence to Antiretroviral Therapy among HIV/AIDS Patients in Sofala Province, in Mozambique: A Retrospective Study. *J AIDS Clin Res*, 4(3), 198.

Powell, C. A., Walker, S. P., Chang, S. M., & Grantham-McGregor, S. M. (1998). Nutrition and education: a randomized trial of the effects of breakfast in rural primary school children. *Am J Clin Nutr*, 68(4), 873-879.

Powell-Jackson, T., & Hanson, K. (2012). Financial incentives for maternal health: impact of a national programme in Nepal. *J Health Econ*, 31(1), 271-284.

Pronyk, P. M., Hargreaves, J. R., Kim, J. C., Morison, L. A., Phetla, G., Watts, C., Busza, J., & Porter, J. D. (2006). Effect of a structural intervention for the prevention of intimate-partner violence and HIV in rural South Africa: a cluster randomised trial. *The Lancet*, 368(9551), 1973-1983.

Pronyk, P. M., Harpham, T., Busza, J., Phetla, G., Morison, L. A., Hargreaves, J. R., Kim, J. C., Watts, C. H., & Porter, J. D. (2008). Can social capital be intentionally generated? A randomized trial from rural South Africa. *Soc Sci Med*, 67(10), 1559-1570.

Pronyk, P. M., Kim, J. C., Abramsky, T., Phetla, G., Hargreaves, J. R., Morison, L. A., Watts, C., Busza, J., & Porter, J. D. (2008). A combined microfinance and training intervention can reduce HIV risk behaviour in young female participants. *AIDS*, 22(13), 1659-1665.

Pronyk, P. M., Muniz, M., Nemser, B., Somers, M. A., McClellan, L., Palm, C. A., Huynh, U. K., Ben Amor, Y., Begashaw, B., McArthur, J. W., Niang, A., Sachs, S. E., Singh, P., Teklehaimanot, A., Sachs, J. D., & Millennium Villages Study, G. (2012). The effect of an integrated multisector model for achieving the Millennium Development Goals and improving child survival in rural sub-Saharan Africa: a non-randomised controlled assessment. *The Lancet*, 379(9832), 2179-2188.

Qouta, S. R., Palosaari, E., Diab, M., & Punamäki, R. L. (2012). Intervention effectiveness among war-affected children: A cluster randomized controlled trial on improving mental health. *Journal of Traumatic Stress*, 25(3), 288-298.

Rahman, A., Mubbashar, M. H., Gater, R., & Goldberg, D. (1998). Randomised trial of impact of school mental-health programme in rural Rawalpindi, Pakistan. *The Lancet*, 352(9133), 1022-1025.

Rahman, M. M. (2012). Estimating the Effects of Social Safety Net Programmes in Bangladesh on Calorie Consumption of Poor Households. *Bangladesh Development Studies*, 35(2), 67-85.

Rahman, M. M. (2014). Estimating the Average Treatment Effect of Social Safety Net Programmes in Bangladesh. *The Journal of Development Studies*, 50(11), 1550-1569.

Rahmani, K., Djazayery, A., Habibi, M. I., Heidari, H., Dorosti-Motlagh, A. R., Pourshahriari, M., & Azadbakht, L. (2011). Effects of daily milk supplementation on improving the physical and mental function as well as school performance among children: results from a school feeding program. *J Res Med Sci*, 16(4), 469-476.

Ramírez-Silva, I., Rivera, J. A., Leroy, J. L., & Neufeld, L. M. (2013). The Oportunidades Program's Fortified Food Supplement, but Not Improvements in the Home Diet, Increased the Intake of Key Micronutrients in Rural Mexican Children Aged 12–59 Months. *The Journal of Nutrition*, 143(5), 656-663.

Rausch Herscovici, C., Kovalskys, I., & De Gregorio, M. J. (2013). Gender differences and a school-based obesity prevention program in Argentina: a randomized trial. *Revista Panamericana de Salud Pública*, 34(2), 75-82.

Raza, W. A., Das, N. C., & Misha, F. A. (2012). Can ultra-poverty be sustainably improved? Evidence from BRAC in Bangladesh. *Journal of Development Effectiveness*, 4(2), 257-276.

Reddy, K. S., Arora, M., Kohli, A., Prabhakaran, D., Perry, C. L., Nair, B., Lytle, L. A., & Stigler, M. (2002). Tobacco and alcohol use outcomes of a school-based intervention in New Delhi. *American Journal of Health Behavior*, 26(3), 173-181.

Rehman, A. M., Woodd, S., PrayGod, G., Chisenga, M., Siame, J., Koethe, J. R., Heimburger, D. C., Kelly, P., Friis, H., & Filteau, S. (2015). Effects on Anthropometry and Appetite of Vitamins and Minerals Given in Lipid Nutritional Supplements for Malnourished HIV-Infected Adults Referred for Antiretroviral Therapy: Results from the NUSTART Randomized Controlled Trial. *Journal of Acquired Immune Deficiency Syndromes*, 68(4), 405-412.

Rerksuppaphol, S., & Rerksuppaphol, L. (2012). Randomized controlled trial of probiotics to reduce common cold in schoolchildren. *Pediatr Int*, 54(5), 682-687.

Resnicow, K., Reddy, S. P., James, S., Gabebodeen Omardien, R., Kambaran, N. S., Langner, H. G., Vaughan, R. D., Cross, D., Hamilton, G., & Nichols, T. (2008). Comparison of two school-based smoking prevention programs among South African high school students: results of a randomized trial. *Ann Behav Med*, 36(3), 231-243.

Ribeiro, R., & Alves, L. (2014). Comparison of two school-based programmes for health behaviour change: The Belo Horizonte Heart Study randomized trial. *Public Health Nutrition*, 17(6), 1195-1204.

Riehl, B., Zerriffi, H., & Naidoo, R. (2015). Effects of Community-Based Natural Resource Management on Household Welfare in Namibia. *PLOS ONE*, 10(5), e0125531.

Rivera, J. A., Sotres-Alvarez, D., & Habicht, J. P. (2004). The Progresa programme improves anaemia and the growth rates of young children in low-income rural Mexico. *Evidence-based Healthcare and Public Health*, 8(6), 381-382.

Rivera, J. A., Sotres-Alvarez, D., Habicht, J. P., Shamah, T., & Villalpando, S. (2004). Impact of the Mexican program for education, health, and nutrition (Progresa) on rates of growth and anemia in infants and young children: a randomized effectiveness study. *JAMA*, 291(21), 2563-2570.

Robertson, L., Mushati, P., Eaton, J. W., Dumba, L., Mavise, G., Makoni, J., Schumacher, C., Crea, T., Monasch, R., Sherr, L., Garnett, G. P., Nyamukapa, C., & Gregson, S. (2013). Effects of unconditional and conditional cash transfers on child health and development in Zimbabwe: a cluster-randomised trial. *The Lancet*, 381(9874), 1283–1292.

Rodriguez, D. G. P., Rejesus, R. M., & Aragon, C. T. (2007). Impacts of an agricultural development program for poor coconut producers in the Philippines: An approach using panel data and propensity score matching techniques. *Journal of Agricultural and Resource Economics*, 534-557.

Rohner, F., Zimmermann, M. B., Amon, R. J., Vounatsou, P., Tschannen, A. B., N'Goran, E. K., Nindjin, C., Cacou, M. C., Té-Bonlé, M. D., Aka, H., Sess, D. E., Utzinger, J., & Hurrell, R. F. (2010). In a Randomized Controlled Trial of Iron Fortification, Anthelmintic Treatment, and Intermittent Preventive Treatment of Malaria for Anemia Control in Ivorian Children, only Anthelmintic Treatment Shows Modest Benefit. *The Journal of Nutrition*, 140(3), 635-641.

Romero, A., Pick, S., Coria, A. P. & Givaudan, A. (2010). Evaluación del Impacto de un Programa de Prevención de Violencia en Adolescentes. *Interamerican Journal of Psychology*, 44(2), 203-212.

Rong, W. S., Bian, J. Y., Wang, W. J., & Wang, J. D. (2003). Effectiveness of an oral health education and caries prevention program in kindergartens in China. *Community Dent Oral Epidemiol*, 31(6), 412-416.

Rosa, G., Majorin, F., Boisson, S., Barstow, C., Johnson, M., Kirby, M., Ngabo, F., Thomas, E., & Clasen, T. (2014). Assessing the Impact of Water Filters and Improved Cook Stoves on Drinking Water Quality and Household Air Pollution: A Randomised Controlled Trial in Rwanda. *PLOS ONE*, 9(3), e91011.

Roschnik, N., Parawan, A., Baylon, M. A., Chua, T., & Hall, A. (2004). Weekly iron supplements given by teachers sustain the haemoglobin concentration of schoolchildren in the Philippines. *Trop Med Int Health*, 9(8), 904-909.

Ross, D. A., Changalucha, J., Obasi, A. I., Todd, J., Plummer, M. L., Cleophas-Mazige, B., Anemona, A., Everett, D., Weiss, H. A., Mabey, D. C., Grosskurth, H., & Hayes, R. J.

(2007). Biological and behavioural impact of an adolescent sexual health intervention in Tanzania: a community-randomized trial. *AIDS*, 21(14), 1943-1955.

Rotheram-Borus, M. J., Lightfoot, M., Kasirye, R., & Desmond, K. (2012). Vocational training with HIV prevention for Ugandan youth. *AIDS Behav*, 16(5), 1133-1137.

Roy, S., Ara, J., Das, N., & Quisumbing, A. R. (2015). “Flypaper effects” in transfers targeted to women: Evidence from BRAC's “Targeting the Ultra Poor” program in Bangladesh. *Journal of Development Economics*, 117(Supplement C), 1-19.

Ruel, M. T., Menon, P., Habicht, J. P., Loechl, C., Bergeron, G., Pelto, G., Arimond, M., Maluccio, J., Michaud, L., & Hankebo, B. (2008). Age-based preventive targeting of food assistance and behaviour change and communication for reduction of childhood undernutrition in Haiti: a cluster randomised trial. *The Lancet*, 371(9612), 588-595.

Ruiz-Arranz, M., Davis, B., Handa, S., Stampini, M., & Winters, P. (2006). Program conditionality and food security: The impact of PROGRESA and PROCAMPO transfers in rural Mexico. *Revista Economía*, 7(2), 249-278.

Rusakaniko, S., Mbizvo, M. T., Kasule, J., Gupta, V., Kinoti, S. N., Mpanju-Shumbushu, W., Sebina-Zziwa, J., Mwateba, R., & Padayachy, J. (1997). Trends in reproductive health knowledge following a health education intervention among adolescents in Zimbabwe. *Cent Afr J Med*, 43(1), 1-6.

Saad, A., Lekhraj, R., Sabitu, K., AbdulRahman, H., Awaisu, A., AbuSamah, B., & Ibrahim, A. (2012). An HIV-STI risk reduction program among undergraduate students at a northern Nigerian university: a randomized controlled field trial. *Journal of Public Health*, 20(5), 549-559.

Saboori, S., Greene, L. E., Moe, C. L., Freeman, M. C., Caruso, B. A., Akoko, D., & Rheingans, R. D. (2013). Impact of Regular Soap Provision to Primary Schools on Hand Washing and E. coli Hand Contamination among Pupils in Nyanza Province, Kenya: A Cluster-Randomized Trial. *The American Journal of Tropical Medicine and Hygiene*, 89(4), 698–708.

Safdie, M., Jennings-Aburto, N., Lévesque, L., Janssen, I., Campirano-Núñez, F., López-Olmedo, N., Aburto, T., & Rivera, J. A. (2013). Impact of a school-based intervention program on obesity risk factors in Mexican children. *Salud Pública de México*, 55(S3), 374-387.

Safdie, M., Lévesque, L., González-Casanova, I., Salvo, D., Islas, A., Hernández-Cordero, S., Bonvecchio, A., & Rivera, J. A. (2013). Promoting healthful diet and physical activity in the Mexican school system for the prevention of obesity in children. *Salud Pública de México*, 55(S3), 357-373.

Saied-Moallemi, Z., Virtanen, J. I., Vehkalahti, M. M., Tehranchi, A., & Murtomaa, H. (2009). School-based intervention to promote preadolescents' gingival health: a community trial. *Community Dent Oral Epidemiol*, 37(6), 518-526.

Sakuma, K. L., Sun, P., Unger, J. B., & Johnson, C. A. (2010). Evaluating depressive symptom interactions on adolescent smoking prevention program mediators: a mediated moderation analysis. *Nicotine Tob Res*, 12(11), 1099-1107.

Samy, M., Khairy, S., Ibrahim, S., Matter, M., & Hassan, H. (2012). Management of Overweight and Obesity in Egyptian School Children-An Intervention Study. *Journal of American Science*, 8(11).

Sanaeinasab, H., Saffari, M., Pakpour, A. H., Nazeri, M., & Piper, C. N. (2012). A model-based educational intervention to increase physical activity among Iranian adolescents. *J Pediatr (Rio J)*, 88(5), 430-438.

Santos, F., Fletschner, D., Savath, V., & Peterman, A. (2014). Can Government-Allocated Land Contribute to Food Security? Intrahousehold Analysis of West Bengal's Microplot Allocation Program. *World Development*, 64(Supplement C), 860-872.

Saraf, D. S., Gupta, S. K., Pandav, C. S., Nongkinrih, B., Kapoor, S. K., Pradhan, S. K., & Krishnan, A. (2015). Effectiveness of a School Based Intervention for Prevention of Non-communicable Diseases in Middle School Children of Rural North India: A Randomized Controlled Trial. *The Indian Journal of Pediatrics*, 82(4), 354-362.

Scales, P. C., Benson, P. L., Dershem, L., Fraher, K., Makonnen, R., Nazneen, S., Syvertsen, A. K., & Titus, S. (2013). Building Developmental Assets to Empower Adolescent Girls in Rural Bangladesh: Evaluation of Project Kishoree Kontha. *Journal of Research on Adolescence*, 23(1), 171-184.

Scarlato, M., d'Agostino, G., & Capparucci, F. (2016) Evaluating CCTs from a Gender Perspective: The Impact of Chile Solidario on Women's Employment Prospect. *Journal of International Development*, 28(2), 177–197.

Schady, N., Araujo, M. C., Peña, X., & López-Calva, L. F. (2008). Cash Transfers, Conditions, and School Enrollment in Ecuador [with Comments]. *Economía*, 43-77.

Schultz, T. P. (2004). School subsidies for the poor: evaluating the Mexican Progresa poverty program. *Journal of Development Economics*, 74(1), 199-250.

Schumann, K., Romero-Abal, M. E., Maurer, A., Luck, T., Beard, J., Murray-Kolb, L., Bulux, J., Mena, I., & Solomons, N. W. (2005). Haematological response to haem iron or ferrous sulphate mixed with refried black beans in moderately anaemic Guatemalan pre-school children. *Public Health Nutr*, 8(6), 572-581.

Scullion, J., Thomas, C. W., Vogt, K. A., Perez-Maqueo, O., & Logsdon, M. G. (2011). Evaluating the environmental impact of payments for ecosystem services in Coatepec (Mexico) using remote sensing and on-site interviews. *Environmental Conservation*, 38(04), 426-434.

Seal, N. (2006). Preventing tobacco and drug use among Thai high school students through life skills training. *Nursing & health sciences*, 8(3), 164-168.

Sedlmayr, R., Fink, G., Miller, J. M., Earle, D., & Steketee, R. W. (2013). Health impact and cost-effectiveness of a private sector bed net distribution: experimental evidence from Zambia. *Malar J*, 12, 102.

Seiber, E. E., & Robinson, A. L. (2007). Microfinance investments in quality at private clinics in Uganda: a case-control study. *BMC Health Services Research*, 7(1), 168.

Serbescu, C., Flora, D., Hantiu, I., Greene, D., Laurent Benhamou, C., & Courteix, D. (2006). Effect of a six-month training programme on the physical capacities of Romanian schoolchildren. *Acta Paediatr*, 95(10), 1258-1265.

Shamah Levy, T., Morales Ruan, C., Amaya Castellanos, C., Salazar Coronel, A., Jimenez Aguilar, A., & Mendez Gomez Humaran, I. (2012). Effectiveness of a diet and physical activity promotion strategy on the prevention of obesity in Mexican school children. *BMC Public Health*, 12, 152.

Sharieff, W., Yin, S. A., Wu, M., Yang, Q., Schauer, C., Tomlinson, G., & Zlotkin, S. (2006). Short-term daily or weekly administration of micronutrient Sprinkles has high compliance and does not cause iron overload in Chinese schoolchildren: a cluster-randomised trial. *Public Health Nutr*, 9(3), 336-344.

Shei, A., Costa, F., Reis, M. G., & Ko, A. I. (2014). The impact of Brazil’s Bolsa Família conditional cash transfer program on children’s health care utilization and health outcomes. *BMC International Health and Human Rights*, 14, 10.

Sherman, S. G., Srikrishnan, A. K., Rivett, K. A., Liu, S. H., Solomon, S., & Celentano, D. D. (2010). Acceptability of a microenterprise intervention among female sex workers in Chennai, India. *AIDS Behav*, 14(3), 649-657.

Shi, L., Zhang, J., Wang, Y., Caulfield, L. E., & Guyer, B. (2010). Effectiveness of an educational intervention on complementary feeding practices and growth in rural China: a cluster randomised controlled trial. *Public Health Nutr*, 13(4), 556-565.

Sichieri, R., Paula Trotte, A., de Souza, R. A., & Veiga, G. V. (2009). School randomised trial on prevention of excessive weight gain by discouraging students from drinking sodas. *Public Health Nutr*, 12(2), 197-202.

Sigman, M., Whaley, S. E., Neumann, C. G., Bwibo, N., Guthrie, D., Weiss, R. E., Liang, L. J., & Murphy, S. P. (2005). Diet Quality Affects the Playground Activities of Kenyan Children. *Food and Nutrition Bulletin*, 26(S2), S202-S212.

Sillsa, E. O., & Caviglia-Harris, J. L. (2015). Evaluating the long-term impacts of promoting 'green' agriculture in the Amazon. *Agricultural Economics*, 46(S1), 83-102.

Simeon, D. T., Grantham-McGregor, S. M., Callender, J. E., & Wong, M. S. (1995). Treatment of Trichuris trichiura infections improves growth, spelling scores and school attendance in some children. *Journal of Nutrition*, 125(7), 1875-1883.

Simwaka, B. N., Simwaka, K., & Bello, G. (2009). Retrospective analysis of a school-based malaria treatment programme demonstrates a positive impact on health and education outcomes in Mangochi district, Malawi. *Journal of Development Effectiveness*, 1(4), 492-506.

Singh, A. S., Kang, G., Ramachandran, A., Sarkar, R., Peter, P., & Bose, A. (2010). Locally made ready-to-use therapeutic food for treatment of malnutrition: A randomized controlled trial. *Indian Pediatrics*, 47(8), 679-686.

Skoufias, E. (2009). PROGRESA and Its Impact on the Welfare of Rural Households in Mexico. *Social Development Issues*, 31(2), 93-97.

Skoufias, E., & Di Maro, V. (2008). Conditional cash transfers, adult work incentives, and poverty. *The Journal of Development Studies*, 44(7), 935-960.

Skoufias, E., Parker, S. W., Behrman, J. R., & Pessino, C. (2001). Conditional cash transfers and their impact on child work and schooling: Evidence from the PROGRESA program in Mexico. *Economía*, 45-96.

Smith, E. A., Palen, L. A., Caldwell, L. L., Flisher, A. J., Graham, J. W., Mathews, C., Wegner, L., & Vergnani, T. (2008). Substance use and sexual risk prevention in Cape Town, South Africa: an evaluation of the HealthWise program. *Prevention Science*, 9(4), 311-321.

Smith, L. C., Khan, F., Frankenberger, T. R., & Wadud, A. (2013). Admissible evidence in the court of development evaluation? The impact of CARE's SHOUHARDO Project on child stunting in Bangladesh. *World Development*, 41, 1-43.

Soares, F. V., Perez Ribas, R., & Issamu Hirata, G. (2010). Impact evaluation of a rural conditional cash transfer programme on outcomes beyond health and education. *Journal of Development Effectiveness*, 2(1), 138-157.

Soofi, S. B., Hussain, I., Mehboob, N., Hussain, M., Bhatti, Z., Khan, S., Hasan, S., & Bhutta, Z. A. (2013). Impoverished Rural Districts of Pakistan: An Independent Evaluation of Impact on Educational and Cognitive Outcomes in Sindh Province, Pakistan. *IDS Bulletin*, 44(3), 48–56.

Sorensen, G., Gupta, P. C., Nagler, E., & Viswanath, K. (2012). Promoting life skills and preventing tobacco use among low-income Mumbai youth: effects of Salaam Bombay Foundation intervention. *PLoS One*, 7(4), e34982.

Sripaipan, T., Schroeder, D. G., Marsh, D. R., Pachon, H., Dearden, K. A., Ha, T. T., & Lang, T. T. (2002). Effect of an integrated nutrition program on child morbidity due to respiratory infection and diarrhea in northern Viet Nam. *Food Nutr Bull*, 23(4 Suppl), 70-77.

Ssewamala, F. M., Alicea, S., Bannon, W. M., & Ismayilova, L. (2008). A Novel Economic Intervention to Reduce HIV Risks Among School-Going AIDS Orphans in

Rural Uganda. *The Journal of Adolescent Health: Official Publication of the Society for Adolescent Medicine*, 42(1), 102–104. http://doi.org/10.1016/j.jadohealth.2007.08.011

Ssewamala, F. M., Han, C. K., & Neilands, T. B. (2009). Asset ownership and health and mental health functioning among AIDS-orphaned adolescents: findings from a randomized clinical trial in rural Uganda. *Soc Sci Med*, 69(2), 191-198.

Ssewamala, F. M., & Ismayilova, L. (2009). Integrating Children's Savings Accounts in the Care and Support of Orphaned Adolescents in Rural Uganda. *The Social Service Review*, 83(3), 453–472.

Ssewamala, F. M., Ismayilova, L., McKay, M., Sperber, E., Bannon, W., Jr., & Alicea, S. (2010). Gender and the effects of an economic empowerment program on attitudes toward sexual risk-taking among AIDS-orphaned adolescent youth in Uganda. *J Adolesc Health*, 46(4), 372-378.

Ssewamala, F. M., Karimli, L., Torsten, N., Wang, J. S. H., Han, C. K., Ilic, V., & Nabunya, P. (2016). Applying a Family-Level Economic Strengthening Intervention to Improve Education and Health-Related Outcomes of School-Going AIDS-Orphaned Children: Lessons from a Randomized Experiment in Southern Uganda. *Prevention Science*, 17(1), 134-143.

Ssewamala, F. M., Neilands, T. B., Waldfogel, J., & Ismayilova, L. The Impact of a Comprehensive Microfinance Intervention on Depression Levels of AIDS-Orphaned Children in Uganda. *Journal of Adolescent Health*, 50(4), 346-352.

Stanton, B. F., Li, X., Kahihuata, J., Fitzgerald, A. M., Neumbo, S., Kanduuombe, G., Ricardo, I. B., Galbraith, J. S., Terreri, N., & Guevara, I. (1998). Increased protected sex and abstinence among Namibian youth following a HIV risk-reduction intervention: a randomized, longitudinal study. *AIDS*, 12(18), 2473-2480.

Stecklov, G., Winters, P., Stampini, M., & Davis, B. (2005). Do conditional cash transfers influence migration? A study using experimental data from the Mexican PROGRESA program. *Demography*, 42(4), 769-790.

Steyn, N. P., de Villiers, A., Gwebushe, N., Draper, C. E., Hill, J., de Waal, M., Dalais, L., Abrahams, Z., Lombard, C., & Lambert, E. V. (2015). Did HealthKick, a randomised controlled trial primary school nutrition intervention improve dietary quality of children in low-income settings in South Africa? *BMC Public Health*, 15(1), 948.

Stigler, M. H., Kugler, K. C., Komro, K. A., Leshabari, M. T., & Klepp, K. I. (2006). AIDS education for Tanzanian youth: a mediation analysis. *Health Educ Res*, 21(4), 441-451.

Stigler, M. H., Perry, C. L., Smolenski, D., Arora, M., & Reddy, K. S. (2011). A mediation analysis of a tobacco prevention program for adolescents in India: how did project MYTRI work? *Health Educ Behav*, 38(3), 231-240.

Stoller, N. E., Gebre, T., Ayele, B., Zerihun, M., Assefa, Y., Habte, D., Zhou, Z., Porco, T. C., Keenan, J. D., House, J. I., Gaynor, B. D., Lietman, T. M., & Emerson, P. M. (2011). Efficacy of latrine promotion on emergence of infection with ocular Chlamydia trachomatis after mass antibiotic treatment: a cluster-randomized trial. *Int Health*, 3(2), 75-84.

Stoltzfus, R. J., Albonico, M., Chwaya, H. M., Tielsch, J. M., Schulze, K. J., & Savioli, L. (1998). Effects of the Zanzibar school-based deworming program on iron status of children. *Am J Clin Nutr*, 68(1), 179-186.

Stoltzfus, R. J., Albonico, M., Tielsch, J. M., Chwaya, H. M., & Savioli, L. (1997). School-based deworming program yields small improvement in growth of Zanzibari school children after one year. *J Nutr*, 127(11), 2187-2193.

Subba Rao, G. M., Rao, D. R., Venkaiah, K., Dube, A. K., & Sarma, K. V. (2006). Evaluation of the Food and Agriculture Organization's global school-based nutrition education initiative, Feeding Minds, Fighting Hunger (FMFH), in schools of Hyderabad, India. *Public Health Nutr*, 9(8), 991-995.

Sulaiman, M. (2015). Does wealth increase affect school enrolment in ultra-poor households: evidence from an experiment in Bangladesh. *Enterprise Development and Microfinance*, 26(2).

Sun, J., Wang, Y., Chen, X., Chen, Y., Feng, Y., Zhang, X., Pan, Y., Hu, T., Xu, J., Du, L., Zhou, W., Zhao, H., Riley, R. E., & Mustad, V. A. (2008). An integrated intervention program to control diabetes in overweight Chinese women and men with type 2 diabetes. *Asia Pac J Clin Nutr*, 17(3), 514-524.

Sun, P., Unger, J. B., Guo, Q., Gong, J., Ma, H., Palmer, P. H., Chou, C. P., Li, Y., Sussman, S., Ritt-Olson, A., Xiao, L., & Johnson, C. A. (2007). Comorbidity between depression and smoking moderates the effect of a smoking prevention program among boys in China. *Nicotine Tob Res*, 9 Suppl 4, S599-609.

Swendeman, D., Basu, I., Das, S., Jana, S., & Rotheram-Borus, M. J. (2009). Empowering sex workers in India to reduce vulnerability to HIV and sexually transmitted diseases. *Soc Sci Med*, 69(8), 1157-1166.

Sylvia, S., Luo, R., Zhang, L., Shi, Y., Medina, A., & Rozelle, S. (2013). Do you get what you pay for with school-based health programs? Evidence from a child nutrition experiment in rural China. *Economics of Education Review*, 37, 1-12.

Tahlil, T., Woodman, R. J., Coveney, J., & Ward, P. R. (2015). Six-months follow-up of a cluster randomized trial of school-based smoking prevention education programs in Aceh, Indonesia. *BMC Public Health*, 15(1), 1088.

Tai, B. J., Jiang, H., Du, M. Q., & Peng, B. (2009). Assessing the effectiveness of a school-based oral health promotion programme in Yichang City, China. *Community Dent Oral Epidemiol*, 37(5), 391-398.

Talaat, M., Afifi, S., Dueger, E., El-Ashry, N., Marfin, A., Kandeel, A., Mohareb, E., & El-Sayed, N. (2011). Effects of hand hygiene campaigns on incidence of laboratory-confirmed influenza and absenteeism in schoolchildren, Cairo, Egypt. *Emerg Infect Dis*, 17(4), 619-625.

Tan, J. P., Lane, J., & Lassibille, G. (1999). Student outcomes in Philippine elementary schools: An evaluation of four experiments. *World Bank Econ Rev*, 13(3), 493-508.

Taneja, S., Bahl, S., Mazumder, S., Martines, J., Bhandari, N., & Kishan Bhan, M. (2015). Impact on inequities in health indicators: effect of implementing the Integrated Management of Neonatal and Childhood Illness programme in Haryana, India. *Journal of Global Health*, 5(1), 010401.

Tavengwa, N. V., Piwoz, E. G., Iliff, P. J., Moulton, L. H., Zunguza, C. D., Nathoo, K. J., Hargrove, J. W., Group, Z. S., & Humphrey, J. H. (2007). Adoption of safer infant feeding and postpartum sexual practices and their relationship to maternal HIV status and risk of acquiring HIV in Zimbabwe. *Trop Med Int Health*, 12(1), 97-106.

Taymoori, P., Niknami, S., Berry, T., Lubans, D., Ghofranipour, F., & Kazemnejad, A. (2008). A school-based randomized controlled trial to improve physical activity among Iranian high school girls. *Int J Behav Nutr Phys Act*, 5, 18.

Thabet, A. A., Vostanis, P., & Karim, K. (2005). Group crisis intervention for children during ongoing war conflict. *Eur Child Adolesc Psychiatry*, 14(5), 262-269.

Thériault, F. L., Maheu-Giroux, M., Blouin, B., Casapía, M., & Gyorkos, T. W. (2014). Effects of a Post-Deworming Health Hygiene Education Intervention on Absenteeism in School-Age Children of the Peruvian Amazon. *PLOS Neglected Tropical Diseases*, 8(8), e3007.

Thi Le, H., Brouwer, I. D., Burema, J., Nguyen, K. C., & Kok, F. J. (2006). Efficacy of iron fortification compared to iron supplementation among Vietnamese schoolchildren. *Nutr J*, 5, 32.

Thirumurthy, H., Masters, S. H., Rao, S., Bronson, M. A., Lanham, M., Omanga, E., Evens, E., & Agot, K. (2014). Effect of providing conditional economic compensation on uptake of voluntary medical male circumcision in Kenya: a randomized clinical trial. *JAMA*, 312(7), 703-711.

Tibbits, M. K., Smith, E. A., Caldwell, L. L., & Flisher, A. J. (2011). Impact of HealthWise South Africa on polydrug use and high-risk sexual behavior. *Health Educ Res*, 26(4), 653-663.

Tijani, A. A., Masuku, M. B., & Raufu, M. O. (2014). The impact of the Fadama II intervention on rural households in Kogi and Kwara States, Nigeria. *American Journal of Experimental Agriculture*, 4(12), 1996-2010.

Tirivayi, N., Koethe, J. R., & Groot, W. (2012). Clinic-Based Food Assistance is Associated with Increased Medication Adherence among HIV-Infected Adults on Long-Term Antiretroviral Therapy in Zambia. *Journal of AIDS & Clinical Research*, 3(7), 171.

Todd, J. E., & Winters, P. (2011). The effect of early interventions in health and nutrition on on-time school enrollment: evidence from the Oportunidades Program in rural Mexico. *Econ Dev Cult Change*, 59(3), 549-581.

Todd, J. E., Winters, P., & Hertz, T. (2010). Conditional Cash Transfers and Agricultural Production: Lessons from the Oportunidades Experience in Mexico. *The Journal of Development Studies*, 46(1), 39-67.

Todd, P. E., & Wolpin, K. I. (2006). Assessing the impact of a school subsidy program in Mexico: Using a social experiment to validate a dynamic behavioral model of child schooling and fertility. *Am Econ Rev*, 1384-1417.

Todo, Y., & Takahashi, R. (2013), Impact of farmer field schools on agricultural income and skills: evidence from an aid-funded project in rural Ethiopia. *Journal of International Development*, 25, 362–381.

Tol, W. A., Komproe, I. H., Jordans, M. J., Gross, A. L., Susanty, D., Macy, R. D., & de Jong, J. T. (2010). Mediators and moderators of a psychosocial intervention for children affected by political violence. *J Consult Clin Psychol*, 78(6), 818-828.

Tol, W. A., Komproe, I. H., Jordans, M. J., Ndayisaba, A., Ntamutumba, P., Sipsma, H., Smallegange, E. S., Macy, R. D., & de Jong, J. T. (2014). School-based mental health intervention for children in war-affected Burundi: a cluster randomized trial. *BMC Med*, 12, 56.

Tol, W. A., Komproe, I. H., Jordans, M. J., Vallipuram, A., Sipsma, H., Sivayokan, S., Macy, R. D., & JT, D. E. J. (2012). Outcomes and moderators of a preventive school-based mental health intervention for children affected by war in Sri Lanka: a cluster randomized trial. *World Psychiatry*, 11(2), 114-122.

Tol, W. A., Komproe, I. H., Susanty, D., Jordans, M. J., Macy, R. D., & De Jong, J. T. (2008). School-based mental health intervention for children affected by political violence in Indonesia: a cluster randomized trial. *JAMA*, 300(6), 655-662.

Toral, N., & Slater, B. (2012). Intervention based exclusively on stage-matched printed educational materials regarding healthy eating does not result in changes to adolescents' dietary behavior. *ScientificWorldJournal*, 174640.

Toruner, E. K., Ayaz, S., Altay, N., Citak, E. A., & Sahin, S. (2015). Efficacy of a School-Based Healthy Life Program in Turkey. *Children's Health Care*, 44(1), 69-86.

Tucunduva Philippi, S., & Barco Leme, A. C. (2015). Dietary intake and meal frequency of Brazilian girls attending a school-based randomized controlled trial. *Nutrition & Food Science*, 45(6), 954-968.

Tutor, M. V. (2014). The impact of Philippines' conditional cash transfer program on consumption. The Philippine Review of Economics, 51(1), 117-161.

Üçkardeş, Y., Özmert, E. N., Ünal, F., & Yurdakök, K. (2009). Effects of zinc supplementation on parent and teacher behaviour rating scores in low socioeconomic level Turkish primary school children. *Acta Pædiatrica*, 98(4), 731-736.

Urquieta, J., Angeles, G., Mroz, T., Lamadrid-Figueroa, H., & Hernandez, B. (2009). Impact of Oportunidades on skilled attendance at delivery in rural areas. *Economic Development and Cultural Change*, 57(3), 539-558.

van den Briel, T., West, C. E., Bleichrodt, N., van de Vijver, F. J., Ategbo, E. A., & Hautvast, J. G. (2000). Improved iodine status is associated with improved mental performance of schoolchildren in Benin. *Am J Clin Nutr*, 72(5), 1179-1185.

van der Hoeven, M., Faber, M., Osei, J., Kruger, A., & Smuts, C. (2016). Effect of African leafy vegetables on the micronutrient status of mildly deficient farm-school children in South Africa: A randomized controlled study. *Public Health Nutrition*, 19(5), 935-945.

van Stuijvenberg, M. E., Dhansay, M. A., Lombard, C. J., Faber, M., & Benade, A. J. (2001). The effect of a biscuit with red palm oil as a source of beta-carotene on the vitamin A status of primary school children: a comparison with beta-carotene from a synthetic source in a randomised controlled trial. *Eur J Clin Nutr*, 55(8), 657-662.

van Stuijvenberg, M. E., Kvalsvig, J. D., Faber, M., Kruger, M., Kenoyer, D. G., & Benade, A. J. (1999). Effect of iron-, iodine-, and beta-carotene-fortified biscuits on the micronutrient status of primary school children: a randomized controlled trial. *Am J Clin Nutr*, 69(3), 497-503.

van Stuijvenberg, M., Faber, M., Dhansay, M., Lombard, C., Vorster, N., & Benadé, A. (2000). Red palm oil as a source of β-carotene in a school biscuit used to address vitamin A deficiency in primary school children. *Int J Food Sci Nutr*, 51(s1), s43-s50.

Vanlerberghe, V., Toledo, M. E., Rodriguez, M., Gomez, D., Baly, A., Benitez, J. R., & Van der Stuyft, P. (2009). Community involvement in dengue vector control: cluster randomised trial. *BMJ*, 338, b1959.

Vazir, S., Engle, P., Balakrishna, N., Griffiths, P. L., Johnson, S. L., Creed-Kanashiro, H., Fernandez Rao, S., Shroff, M. R., & Bentley, M. E. (2013). Cluster-randomized trial on complementary and responsive feeding education to caregivers found improved dietary intake, growth and development among rural Indian toddlers. *Matern Child Nutr*, 9(1), 99-117.

Verhofstadt, E., & Maertens, M. (2015). Can Agricultural Cooperatives Reduce Poverty? Heterogeneous Impact of Cooperative Membership on Farmers' Welfare in Rwanda. *Applied Economic Perspectives and Policy*, 37(1), 86-106.

Walker, D., Gutierrez, J. P., Torres, P., & Bertozzi, S. M. (2006). HIV prevention in Mexican schools: prospective randomised evaluation of intervention. *BMJ*, 332(7551), 1189-1194.

Walker, S. P., Chang, S. M., Powell, C. A., Simonoff, E., & Grantham-McGregor, S. M. (2006). Effects of psychosocial stimulation and dietary supplementation in early childhood on psychosocial functioning in late adolescence: follow-up of randomised controlled trial. *British Medical Journal*, 333(7566), 472.

Wang, Y., Huang, Z., Yang, M., Wang, F., & Xiao, S. (2015). Reducing Environmental Tobacco Smoke Exposure of Preschool Children: A Randomized Controlled Trial of Class-Based Health Education and Smoking Cessation Counseling for Caregivers. *International Journal of Environmental Research and Public Health*, 12(1), 692.

Wanjala, B. M., & Muradian, R. (2013). Can big push interventions take small-scale farmers out of poverty? Insights from the Sauri Millennium Village in Kenya. *World Development*, 45, 147-160.

Watkins, W. E., Cruz, J. R., & Pollitt, E. (1996). The effects of deworming on indicators of school performance in Guatemala. *Transactions of The Royal Society of Tropical Medicine and Hygiene*, 90(2), 156-161.

Weber, J. G., Sills, E. O., Bauch, S., & Pattanayak, S. K. (2011). Do ICDPs work? An empirical evaluation of forest-based microenterprises in the Brazilian Amazon. *Land Economics*, 87(4), 661-681.

Weldegebriel, Z. B., & Prowse, M. (2013). Climate-Change Adaptation in Ethiopia: To What Extent Does Social Protection Influence Livelihood Diversification? *Development Policy Review*, 31(s2), o35-o56.

Wen, X., Chen, W., Gans, K. M., Colby, S. M., Lu, C., Liang, C., & Ling, W. (2010). Two-year effects of a school-based prevention programme on adolescent cigarette smoking in Guangzhou, China: a cluster randomized trial. *Int J Epidemiol*, 39(3), 860-876.

West, S., Muñoz, B., Lynch, M., Kayongoya, A., Chilangwa, Z., Mmbaga, B., & Taylor, H. R. (1995). Impact of face-washing on trachoma in Kongwa, Tanzania. *The Lancet*, 345(8943), 155-158.

WHO/CHD Immunization-Linked Vitamin A Supplementation Study Group. Randomised trial to assess benefits and safety of vitamin A supplementation linked to immunisation in early infancy. (1998). *The Lancet*, 352(9136), 1257-1263.

Widen, E. M., Bentley, M. E., Chasela, C. S., Kayira, D., Flax, V. L., Kourtis, A. P., Ellington, S. R., Kacheche, Z., Tegha, G., Jamieson, D. J., van der Horst, C. M., Allen, L. H., Shahab-Ferdows, S., Adair, L. S., & Team, f. t. B. S. (2015). Antiretroviral Treatment Is Associated With Iron Deficiency in HIV-Infected Malawian Women That Is Mitigated With Supplementation, but Is Not Associated With Infant Iron Deficiency During 24

Weeks of Exclusive Breastfeeding. *JAIDS Journal of Acquired Immune Deficiency Syndromes*, 69(3), 319-328.

Winters, P., Stecklov, G., & Todd, J. (2009). Household Structure and Short-Run Economic Change in Nicaragua. *Journal of Marriage and Family*, 71(3), 708-726.

Witte, S. S., Aira, T., Tsai, L. C., Riedel, M., Offringa, R., Chang, M., El-Bassel, N., & Ssewamala, F. (2015). Efficacy of a Savings-Led Microfinance Intervention to Reduce Sexual Risk for HIV Among Women Engaged in Sex Work: A Randomized Clinical Trial. *American Journal of Public Health*, 105(3), e95-e102.

Wong, H. L., Luo, R., Zhang, L., & Rozelle, S. (2013). The impact of vouchers on preschool attendance and elementary school readiness: A randomized controlled trial in rural China. *Economics of Education Review*, 35, 53-65.

Wong, H. L., Shi, Y., Luo, R., Zhang, L., & Rozelle, S. (2014). Improving the Health and Education of Elementary Schoolchildren in Rural China: Iron Supplementation Versus Nutritional Training for Parents. *The Journal of Development Studies*, 50(4), 502-519.

Wydick, B., Glewwe, P., & Rutledge, L. (2013). Does international child sponsorship work? A six-country study of impacts on adult life outcomes. *Journal of Political Economy*, 121(2), 393-436.

Xu, F., Ware, R. S., Leslie, E., Tse, L. A., Wang, Z., Li, J., & Wang, Y. (2015). Effectiveness of a Randomized Controlled Lifestyle Intervention to Prevent Obesity among Chinese Primary School Students: CLICK-Obesity Study. *PLOS ONE*, 10(10), e0141421.

Yazdani, R., Vehkalahti, M. M., Nouri, M., & Murtomaa, H. (2009). School-based education to improve oral cleanliness and gingival health in adolescents in Tehran, Iran. *Int J Paediatr Dent*, 19(4), 274-281.

Yekaninejad, M. S., Eshraghian, M. R., Nourijelyani, K., Mohammad, K., Foroushani, A. R., Zayeri, F., Pakpour, A. H., Moscowchi, A., & Tarashi, M. (2012). Effect of a school-based oral health-education program on Iranian children: results from a group randomized trial. *Eur J Oral Sci*, 120(5), 429-437.

Zeba, A. N., Prével, Y. M., Somé, I. T., & Delisle, H. F. (2006). The positive impact of red palm oil in school meals on vitamin A status: study in Burkina Faso. *Nutrition Journal*, 5(1), 17.

Zeng, Y., Keay, L., He, M., Mai, J., Munoz, B., Brady, C., & Friedman, D. S. (2009). A randomized, clinical trial evaluating ready-made and custom spectacles delivered via a school-based screening program in China. *Ophthalmology*, 116(10), 1839-1845.

Zhang, L., Kleiman-Weiner, M., Luo, R., Shi, Y., Martorell, R., Medina, A., & Rozelle, S. (2013). Multiple Micronutrient Supplementation Reduces Anemia and Anxiety in Rural China’s Elementary School Children. *The Journal of Nutrition*, 143(5), 640-647.

Zhu, K., Du, X., Cowell, C. T., Greenfield, H., Blades, B., Dobbins, T. A., Zhang, Q., & Fraser, D. R. (2005). Effects of school milk intervention on cortical bone accretion and indicators relevant to bone metabolism in Chinese girls aged 10-12 y in Beijing. *Am J Clin Nutr*, 81(5), 1168-1175.

Zlotkin, S., Newton, S., Aimone, A. M., & et al. (2013). Effect of iron fortification on malaria incidence in infants and young children in ghana: A randomized trial. *JAMA*, 310(9), 938-947.
