# Supplementary material for: Evaluating the impact of integrated development: are we asking the right questions? A systematic review
Source: Gates Open Res. 2018 May 29;1:6. Originally published 2017 Nov 6. [Version 2] doi: 10.12688/gatesopenres.12755.2 (PMC6034098; doi:10.12688/gatesopenres.12755.2)
Supplement: Peer review history

# Peer review: Referee response for version 2

**DOI:** 10.21956/gatesopenres.13898.r26472

**Published:** 2018-07-30

**Authors:** Thomas de Hoop

**Affiliations:** 1 American Institutes for Research, Washington, DC, USA

This systematic review aims to identify and provide an overview of studies that measure the synergistic effects of integrated development approaches. To achieve this goal, the study screened and reviewed articles included in The International Initiative for Impact Evaluation's (3ie) Impact Evaluation Repository. The review addresses an important research question. However, I still see a number of limitations that are not fully addressed in the current version of the paper.

 As discussed in my previous review, the authors exclude studies that were not published in peer-reviewed journals in the screening and review of the articles. I do not think this was an appropriate methodological choice. In addition, the authors do not take into consideration trade-offs between internal validity and construct validity in their conclusions.

 In their new version the authors acknowledge that it would be valuable to include grey literature in future reviews. They mention that grey literature was not reviewed to manage the high volume of eligible papers. The authors also acknowledge that their exclusion of grey literature could have led to systematic biases in their review. However, they do not discuss specific reasons on why grey literature could have led to different results. The authors only report that “Although our judgement is that it would not be significant, we do acknowledge that reviewing to include grey literature could conceivable shift some of the trends we highlight here.” I would like to ask the authors to explain that judgement in more detail. Studies that measure synergistic effects are often underpowered to detect small but meaningful effects. As a result, publication bias may be larger among studies that focus on the synergistic effects of integrated development approaches. This important limitation is not discussed in detail in the current version of the paper, and I am not convinced that publication bias has not resulted in systematic biases in the review. Many systematic reviews have shown that excluding grey literature can lead to systematic biases.   

 The authors also acknowledge that the external validity of RCTs that study synergistic effects is likely limited. They, however, do not acknowledge that the construct validity of RCTs that aim to measure synergistic effects is likely smaller than for RCTs with only one treatment arm. The reason is that researchers often drive the implementation of programs that are evaluated to measure synergistic effects; as a result most studies that measure synergistic effects estimate the efficacy and not the effectiveness of development programs. I would have liked to see a discussion of how many studies that measure synergistic effects focused on implementation of government programs. My expectation is that this percentage is very small, which further reduces the external and construct validity of RCTs that aim to measure synergistic effects. I would like to ask the authors to discuss these limitation in somewhat more detail.

 This paper already provides an important contribution to the literature, but I think the paper can be further strengthened by discussing these two limitations in more detail.

I have read this submission. I believe that I have an appropriate level of expertise to confirm that it is of an acceptable scientific standard, however I have significant reservations, as outlined above.

# Peer review: Referee response for version 2

**DOI:** 10.21956/gatesopenres.13898.r26474

**Published:** 2018-07-06

**Authors:** Donald C. Cole, Erica Di Ruggiero, Leah Shipton

**Affiliations:** 1 Dalla Lana School of Public Health, University of Toronto, Toronto, ON, Canada

**Overall Comment**

 - This is an easily readable and transparent/clear article that makes new and useful contributions to the area of integrated development interventions.

 - For the most part the authors addressed reviewer comments from the first peer review report.

**Reviewer Comments Addressed by Authors** 

**Rationale and Objectives**

 - The concerns raised about the rationale and objectives in the first peer review form were adequately addressed by the authors. The objectives of the study now more directly match the rationale and the results. In particular, the authors have clarified that they are interested in the study designs of evaluations on integrated development interventions.

**Methods**

 - The authors have done well to clarify the search strategy/sampling of the International Initiative for Impact Evaluation (3ie) Impact Evaluation Repository, which was less clear in the original manuscript. They justify their use of the Impact Evaluation Repository as the only source of articles.

 - The authors do well to clarify their definition of integrated development intervention and how they used the definition to inform the assessment of potential studies to include in their review.

**Results and Discussion**

 - Appreciate the addition of two tables expanding on commonly integrated sectors and proportion of outcomes by sector intervention. Very clearly presented.

 - Authors expanded well on limitations and future directions of the research.

**Remaining Comments for Address**

 - Can the authors add a qualifier about why they limited it to English-only articles?

 - Can the authors clarify what they mean by context. The authors simply discuss integrated development interventions as having positive impacts in different contexts, but do not define what they mean by context. Do the authors mean geographical location?

We have read this submission. We believe that we have an appropriate level of expertise to confirm that it is of an acceptable scientific standard.

# Peer review: Referee response for version 2

**DOI:** 10.21956/gatesopenres.13898.r26473

**Published:** 2018-06-12

**Authors:** Reshma Trasi

**Affiliations:** 1 Pathfinder International, Washington, DC, USA

I am glad that my review was helpful to the authors and they have addressed many of my suggestions. I am happy to recommend that this paper be published in this esteemed journal.

I have read this submission. I believe that I have an appropriate level of expertise to confirm that it is of an acceptable scientific standard.

# Peer review: Referee response for version 1

**DOI:** 10.21956/gatesopenres.13815.r26082

**Published:** 2017-12-20

**Authors:** Thomas de Hoop

**Affiliations:** 1 American Institutes for Research, Washington, DC, USA

This systematic review aims to identify and provide an overview of studies that measure the synergistic effects of integrated development approaches. To achieve this goal, the study screened and reviewed articles included in The International Initiative for Impact Evaluation's (3ie) Impact Evaluation Repository. The review addresses an important research question. The approach is rigorous and the objectives of the study are clear. However, the authors also exclude studies that were not published in peer-reviewed journals in the screening and review of the articles. I do not think this was an appropriate methodological choice. In addition, the authors do not take into consideration trade-offs between internal validity and construct validity in their conclusions. I highlight these issues in more detail below in addition to some other smaller comments. The authors make an important contribution to the literature but I would appreciate it if they would take these comments into consideration when they resubmit the paper. 

**Excluding studies that are not published in peer-reviewed journals:**

 The authors explain that they exclude studies that were not included in peer-reviewed journals. It is unclear why they make this choice. It is well known that the findings of peer-reviewed papers can systematically differ from the findings of articles that are not published in peer-reviewed journals. This may be either because of quality differences or publication bias. Risk of bias assessments can mitigate concerns regarding the former. Therefore, it is unclear why the authors exclude the studies that did not appear in peer-reviewed journals. The authors highlight that of the 26 full factorial papers, seven demonstrated that the full factorial arm was most effective. It is very well possible that this percentage may be systematically different in articles that were not published in peer-reviewed journals because of publication bias. So excluding non-peer reviewed studies may well have led to a bias in the results. It may have been more appropriate to conduct a risk of bias assessment. 

**Internal validity versus construct validity**

 The authors argue in favor of designing and implementing impact evaluations with full factorial designs. I do not disagree with this view. It is important to conduct studies with full factorial designs. However, the reality is that such impact evaluations are very hard to implement in practice in settings without full control of researchers. It is much more challenging to design and implement impact evaluations with a full factorial design when working within government systems or when the intervention is implemented at scale. In contrast, it may be relatively easy (with an emphasis on relatively) to implement a full factorial design when the intervention is designed and implemented by researchers.

 The impact evaluation with full control of the intervention may have a high interval validity but the construct validity of the latter design may be much more limited. Estimated effect sizes from full factorial designs can probably not be credibly extrapolated to settings in which the government has full control over the program. So we need to be careful when we recommend impact evaluations with full factorial designs. Their construct validity may be limited if the government or another large implementing partner is unlikely to implement the program with a full factorial design in practice. Or the take-up of the program is much more limited. For example, Bold et al. (2013) ^1^ demonstrate that the effects of remedial education programs can differ significantly depending on whether they are implemented by an NGO or by the government.  

 Similarly, full factorial designs may sometimes not be relevant. For example, we conducted a study on the effects of a teacher training program that aimed to promote positive gender socialization in Karamoja, Uganda ^2^. This cluster-randomized controlled trial included two treatment arms and a control group. The first treatment arm only included the teacher training. The second treatment arm included the teacher training + an intervention that included text messages related to the contents of the training ^2^. In practice, it would not have been useful to design and implement the latter intervention without the teacher training. But as a result the evaluation does not qualify as a full factorial design according to the definition of this study. I do not think this is a limitation of the study by Chinen et al. (2017). Instead, it shows that full factorial designs are not always the best solution to measure synergistic effects of development programs.  

**Other Comments**

 The researchers also highlight the option of analyzing synergistic effects using qualitative research. It would be helpful to learn a bit more about the specific qualitative research the authors would have in mind to measure synergistic effects.  

 The 3ie Impact Evaluation repository gives a comprehensive overview of impact evaluations in international development. However, it is very well possible that including studies from other databases would have changed the results. It would be good to acknowledge this. 

 The authors do not assess the conditions under which integrated interventions are more effective. However, the authors highlight this as one of the goals of the study. I would remove this objective.

I have read this submission. I believe that I have an appropriate level of expertise to confirm that it is of an acceptable scientific standard, however I have significant reservations, as outlined above.

## References

[1] BoldTKimenyiMMwabuGAlice Ng'ang'aASandefurJ: Scaling Up What Works: Experimental Evidence on External Validity in Kenyan Education. *SSRN Electronic Journal*.2013; 10.2139/ssrn.2241240 10.2139/ssrn.2241240

[2] ChinenMCoombesADe HoopTCastro-ZarzurRElmeskiM: Can Teacher Training Programs Influence Gender Norms? Mixed-MethodsExperimental Evidence from Northern Uganda. *Journal on Education in Emergencies*.2017;3(1) :44-78

# Peer review: Referee response for version 1

**DOI:** 10.21956/gatesopenres.13815.r26084

**Published:** 2017-12-04

**Authors:** Reshma Trasi

**Affiliations:** 1 Pathfinder International, Washington, DC, USA

The paper and its subject is extremely timely and relevant given the integrated development focus of the SDGs. I found the methodology, rationale for the sampling frame, phases of the literature review to be well reasoned and laid out. The subject matter of this paper, especially the 38 studies, could prove to be extremely helpful for implementation practitioners, researchers, and policy makers as sector-specific communities grapple with the complexity of designing and delivering integrated development programs in support of the SDGs.

 I submit, below, a few constructive suggestions for the authors’ consideration to tighten and focus (in some places) or expand (in others) the paper to make it even more useful for the broader development community.

- The authors may want to consider adding the word “impact” into the title itself. “Evaluating the Impact of Integrated Development: are we asking the right questions? A Systematic Review”, perhaps?
- In the **Introduction** section, the authors correctly note, “To effectively advocate for integrated, multi-disciplinary approaches to development, it behooves us to understand **under which circumstances** integrating two or more development sectors enhances impacts in amplified or synergistic ways.” (emphasis added). I did not find this addressed in the Findings section. I strongly recommend that the Findings section be bolstered to focus on the findings from the 38 studies to discuss this critical element. Or, consider deleting this as a rationale for the paper to focus it on the objectives mentioned.
- The authors also state, “In this paper we present the results of a systematic review designed to identify if and how synergies and interaction effects between sectors in integrated interventions are being measured.” It will help to clarify that “how” refers to the methods used (experimental vs quasi experimental; # of sectors for which outcomes are measured, etc.) rather than the specific outcomes being measured and evaluated in the integrated programs. [That said, ‘diversity of outcome measures used in integrated programs’ is a paper that I would urge the authors to consider writing next.]
- **Definitional clarity**:
  - I recommend including a little more discussion around the definition that the authors choose for” integrated development” – what other definitions did you consider? Why did you find them lacking? How did you arrive at this definition? How did you define “delivery” of programs? How did you assess, in your review of the papers, “ intentional linkage”? How did you assess, in your review, if the interventions were linked by “design” (which I would consider being important because it demonstrates a planned approach)?
  - I struggled with semi-equating integrated development with “multi-disciplinary”. Multi-sectoral is probably the term the authors may want to stick closer to. Multi-disciplinary is more of a research term with definitions of its own.
  - I would imagine that there is wide diversity in the outcomes that each sector uses. It may help to give an example for what counted as an outcome for each sector. It will help ground the paper a bit more for the reader.
- In the **Methods** and **Limitations** section, there are a few steps and limitations missing – language, focus on LMICs, etc. This has been well covered by another reviewer, so I will not repeat these here.
- In the **Discussions** section, I recommend two things for the authors’ consideration:
  - An emphasis on the 38 studies. I would have liked to see a separate table that digs into these studies a little more. – what did these show? Where and on what type of integrated development do these focus vis-à-vis the 601 studies? What are they measuring? What synergistic outcomes did you see? Why do you think this is the case?
  - A discussion on gaps - what gaps did you see?
- In the **Conclusions** section, consider adding a paragraph on the utility value of this important endeavor for various audiences. What are the key takeaways for this audience? Or, perhaps, consider adding an impact evaluation agenda? What are the implications for reaching the SDGs from your viewpoint?

I trust these are helpful. Thank you for the opportunity to review this paper.

I have read this submission. I believe that I have an appropriate level of expertise to confirm that it is of an acceptable scientific standard, however I have significant reservations, as outlined above.

# Peer review: Referee response for version 1

**DOI:** 10.21956/gatesopenres.13815.r26081

**Published:** 2017-11-22

**Authors:** Donald C. Cole, Erica Di Ruggiero, Leah Shipton

**Affiliations:** 1 Dalla Lana School of Public Health, University of Toronto, Toronto, ON, Canada

The authors present an interesting review of integrated development approaches and are right to highlight this as an important area of research. The structure of the review is clear and easy to follow the argument and ideas of the authors.

**Introduction - Rationale**

- It would be helpful to have “integrated development intervention” defined earlier in the manuscript, especially because authors mention that there has yet to be consensus on a definition in the literature. Providing an example of an integrated development intervention (including a brief description of which features make it an integrated development intervention) would be helpful in explaining why they are so important in the context of the SDGs.
- The statement below was part of the rationale for conducting this study, but the objectives do not reflect this rationale and neither do the results or discussion sections of the study. Specifically, the authors do not analyze the circumstances when an integrated intervention works. I recommend that this sentence be removed or rephrased to reflect the objectives and results/discussion of the study.
  - "To effectively advocate for integrated, multi-disciplinary approaches to development, it behooves us to understand under which circumstances integrating two or more development sectors enhances impacts in amplified or synergistic ways."
  - The authors might consider circumstances of evaluation in terms of implementation, commitment by participating sectors, funding available, or context (sociocultural, political, institutional, geographical, economic, etc – and this context needs to be defined). For example, how did the institutional structure of sectors involved in an integrated intervention influence the interventions success or failure? How does the geographic context of the integrated intervention influence its success or failure? Etc.

**Introduction - Objectives **

- The secondary objective of the review seems unnecessary because the authors should state the characteristics of the included studies in the results section as part of the systematic review, therefore this does not need to be an objective.
- Regarding the first objective, it would be helpful to have clarification of what the authors mean by “how.” By the end of the article it was clear that the authors mean “how” in terms of evaluation study design (e.g. experimental, factorial) rather than “how” in terms of type of evaluation (e.g. process, outcomes, impact). However, it would be helpful to have this clarified in the objective statement. Specifically, the authors should clarify that they mean to summarize how impact evaluations have been conducted on integrated development programs.

**Methods **

- Selecting the International Initiative for Impact Evaluation (3ie) Impact Evaluation Repository, which only has impact evaluations, as the sole source of studies for this review is another reason why the authors should consider clarifying that the review aims to summarize how impact evaluations have been conducted on integrated development programs.
- In response to criticism that the MDGs only focused on LMICs, the SDGs were supposed to apply to all countries, irrespective of “development’” status. Based on the definition of integrated development interventions/approaches provided by the authors, these approaches are not dependent on location, i.e. an integrated development approach can take place in any country as long as it “intentionally links the design and delivery of programs across more than one core sector.” 3ie search strategy limited the scope to LMICs and LMIC regions. If the SDGs – which are meant to apply to all countries – are invoked as rationale for this systematic review, then it might be helpful to have a comment from the authors for excluding integrated approaches in LMIC contexts.
- The 3ie repository search strategy limited inclusion criteria to English language articles. The authors make no comment on how this could have excluded potentially relevant evaluations for their review, especially if evaluation reports were published by governments and especially considering that these evaluations were conducted in LMIC contexts. 
- This is not a typical systematic review because the authors did not design the search protocol or conduct the literature search stages of the systematic review. The authors provide sound justification for their decision to use the 3ie repository to cover these aspects of the systematic review process. However, the authors might consider repositioning this article as a modified approach to a systematic review. The authors provide a link to the inclusion criteria and review methodology. However, it may be helpful if the authors provided brief details of the search protocol and search strategy, for example: language criteria, key terms, databases chosen, inclusion of grey literature. These are aspects of the systematic review search that helps the reader assess the appropriateness of the search and the articles included for analysis.
- It would be helpful for the following sentence on page 4 to be rephrased: “The 3ie review process has no restriction on publication date; however, the systematic review upon which it is based was completed in July, 2016.” I needed to review the 3ie repository to understand what this sentence means – that a search of 45 databases was conducted in August 2016, which is where the authors sourced their articles for this review.
  - The authors should also comment on the limitations of this strategy in their limitations section of the manuscript.
- The authors consulted a library information science specialist to audit the methodology. The specialist affirmed that the 3ie repository search included the most relevant databases, however, there were lesser known databases excluded from the search. The authors do not search these databases separately. It would be beneficial if the authors commented on/justified their decision to exclude these databases, despite feedback from a specialist that they may contain relevant articles for the review. The search in these databases could have used the same terms as the 3ie repository search strategy.
- The authors state that there is no universal agreement on an integrated development approach definition and then they present their definition (presumably FHI 360 because of author affiliations, although this should be stated more clearly), which is used for the review. If space is available, it would be helpful to see other definitions of integrated development approaches and justification by the authors as to why their definition was chosen for the review. It would be beneficial to comment on why the authors support a definition that excludes interventions that integrate subsectors of a core sector or interventions that measure multiple sector outcomes for interventions that do not have a multi-sector component. Arguably, these two intervention approaches could also have amplified impacts worth understanding. This discussion around definition may be most appropriate in the introduction section of the manuscript.
- The authors should be more explicit on how the definition of integrated development approaches influenced the review process and inclusion/exclusion criteria.
- The authors should clarify how they decided on the list of sectors for their review such that another set of researchers would come up with the same list of sectors if they replicated the study.
- The authors excluded grey literature articles, and based on the results, setting this criterion excluded 1,163 potentially relevant articles. The authors should justify their decision to exclude this grey literature, especially considering that many impact evaluations may not be published in peer reviewed literature.
  - This is another point that the authors should comment on in the limitations section.

**Results**

- It would be interesting to know who conducted the evaluations included in the review (e.g. NGOs, government, UN agencies) and for whom the evaluations were conducted (e.g. consultants hired by government). This information would provide additional insight on the state of integrated development approaches and provide an understanding of who is interested in these evaluations and who is applying the findings of these evaluations.
- It would be interesting to know if there were any sectors that were commonly integrated (e.g. health and nutrition sectors). The commonality of pairings may provide interesting insight on the current state of integrated approaches to development in terms of which sectors are most often paired.
- In the table, the authors present “sectors included in a study intervention” (e.g. 238/601 (40%) of studies included the economic development sector in the intervention) and “sector with outcomes measured” (e.g. 102/601 (17%) of studies had economic development outcomes measured). It may be interesting to have the authors present the numerator as “sector with outcomes measured” over the denominator as “sectors included in a study intervention.” This would illustrate, of the articles that had X development sector involved, what proportion measured X sector outcomes. In the case of the economic development sector, 43% of studies (102/238) that included the economic development sector also had economic development outcomes measured.
- It seems unnecessary for the authors to comment on whether the included articles had a qualitative component considering that the eligibility criteria for the review excluded qualitative evaluation design methods.

**Discussion and Conclusion**

- The results show that of the 26 full factorial evaluations, seven reported the integrated arm as more effective, eight had mixed findings, and 11 reported no added value of the integrated arm. This finding relates to the author’s objective to “document if synergies are detected.” The authors should comment on this finding in the discussion section, rather than only commenting on the number of full factorial evaluations conducted and the feasibility considerations associated with this evaluation study design.
  - Specifically, what synergies were detected/how are those synergies defined? How does this finding relate back to the authors definition of integrated development approaches?
- The authors comment that cost efficiency evaluations and qualitative assessments of synergies are also valuable methods to understand the impact of integrated interventions, yet these methods to evaluation were excluded from the review. The authors might consider expanding on why they excluded studies that used these methods and/or comment on whether future research should include evaluation of integrated approaches using these methods.
- It might be interesting for the authors to comment on future directions for research on integrated development interventions.
- The authors state that “Our systematic review is not intended to determine whether or not integrated development approaches work. We know from the high number of randomized evaluations included here that in many contexts integrated, multi-sector interventions have produced positive impacts.” However, the authors did not provide readers with any results on the effectiveness of integrated approaches using RCT evaluations in the results section of the manuscript.  
  - Also, what do the authors mean by context? Location?

We have read this submission. We believe that we have an appropriate level of expertise to confirm that it is of an acceptable scientific standard, however we have significant reservations, as outlined above.
